# Scaffold Hopping from Dehydrozingerone: Design, Synthesis, and Antifungal Activity of Phenoxyltrifluoromethylpyridines

**DOI:** 10.3390/ijms26115345

**Published:** 2025-06-02

**Authors:** Xiaohui Nan, Kaifeng Wang, Xinru Sun, Zhan Hu, Ranfeng Sun

**Affiliations:** Key Laboratory of Green Prevention and Control of Tropical Plant Diseases and Pests, Ministry of Education, School of Tropical Agriculture and Forestry, Hainan University, Danzhou 571737, China

**Keywords:** scaffold hopping, trifluoromethyl dehydrozingerone, phenoxytrifluoromethylpyridine, antifungal activity, molecular docking

## Abstract

In response to the urgent need for innovative fungicides to ensure food security and safety, a series of twenty-three novel trifluoromethylpyridine compounds were designed and synthesized using a scaffold hopping strategy derived from dehydrozingerone. This approach involved converting the α, β-unsaturated ketone moiety into a pyridine ring. Bioassay results indicated that the majority of these compounds exhibited promising in vitro antifungal activity, particularly against *Rhizoctonia solani* and *Colletotrichum musae*. Notably, compound **17** showed the highest efficacy and broad-spectrum activity, with median effective concentrations (EC_50_) ranging from 2.88 to 9.09 μg/mL. Phenoxytrifluoromethylpyridine derivatives, including compound **17**, exhibited superior antifungal activity compared to benzyloxytrifluoromethylpyridine derivatives. In vivo tests revealed that both compounds **17** and **23** exhibited moderate control effects against *C. musae*. The degradation half-lives of compounds **17** and **23** in bananas were determined to be 176.9 h and 94.8 h, respectively, indicating the stability of their structures in the environment. Molecular docking studies indicated that compound **23** interacts with succinate dehydrogenase, offering valuable insights for the structural optimization of compound **23**.

## 1. Introduction

Fungicides represent a critical component in the management of plant diseases, which pose a significant challenge to the sustainable development of agriculture [1,2]. However, the emergence and spread of new crop pathogens [3], the development of fungicide resistance [4], and the increasing public demand for food safety and environmental protection have collectively rendered the development of new fungicides an urgent priority [2,5]. Natural products have consistently served as a vital resource for the discovery of new pesticides, and the structural modification of natural compounds is a widely adopted strategy in the pursuit of environmentally friendly pesticides [6,7].

**Figure 1:**
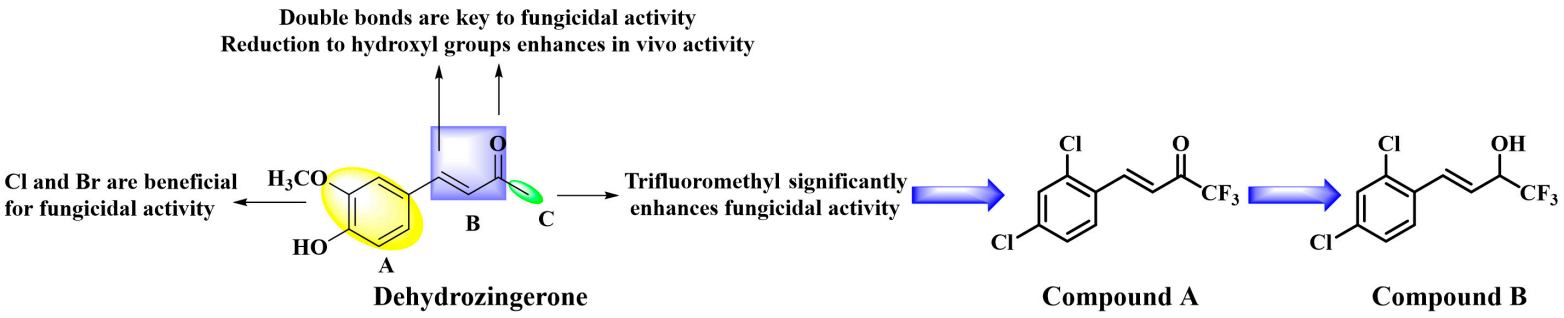
Structure-activity relationship of dehydrozingerone derivatives in antifungal activity.

Dehydrozingerone, derived from the ginger plant *Aframomum giganteum* [8], has garnered significant interest for its diverse pharmacological activities, such as its antioxidant, antimutagenic, anticancer, anti-inflammatory, antidepressant, antimalarial, antiplatelet, Alzheimer’s-disease-preventive, antimicrobial, and β-adrenoceptor antagonist properties [9,10]. In contrast, the application of dehydrozingerone in agriculture is less documented. Notably, it exhibits moderate insect growth-regulatory effects and antifeeding activity against *Spodoptera litura*, as well as potent antifungal action against *Rhizoctonia solani* [11]. Moreover, gingerone derivatives have been shown to attract *Bactrocera jarvisi*, indicating their potential as male chemical lures for integrated pest management [12]. These derivatives also suppress the growth of *Aspergillus flavus* and *Fusarium graminearum* and hinder the biosynthesis of their mycotoxins, underscoring their potential as natural food preservatives [13]. In our recent endeavors to develop novel pesticides derived from natural products, we have successfully designed and synthesized a series of derivatives based on dehydrozingerone as the primary scaffold [14,15] and studied their antifungal structure-activity relationships. Notably, the incorporation of electron-withdrawing groups, such as chlorine, on the benzene ring (Part A, Figure 1) significantly contributes to the antifungal properties [14]. Furthermore, the presence of double bonds and ketone carbonyls (Part B, Figure 1) is imperative for the preservation of antifungal activity [11]. Additionally, the introduction of a trifluoromethyl substituent (Part C, Figure 1) is found to be advantageous in augmenting the antifungal potential of these derivatives [14]. We discovered that compound **A** (Figure 1) exhibits remarkable in vitro antifungal efficacy [14]. However, similar to numerous dehydrozingerone derivatives [15], compound **A** is unstable and exhibits poor in vivo antifungal activity [12]. To address this, we converted the carbonyl group in compound **A** to a hydroxyl group, forming compound **B** (Figure 1), which significantly improved its in vivo activity [15].

**Figure 2:**
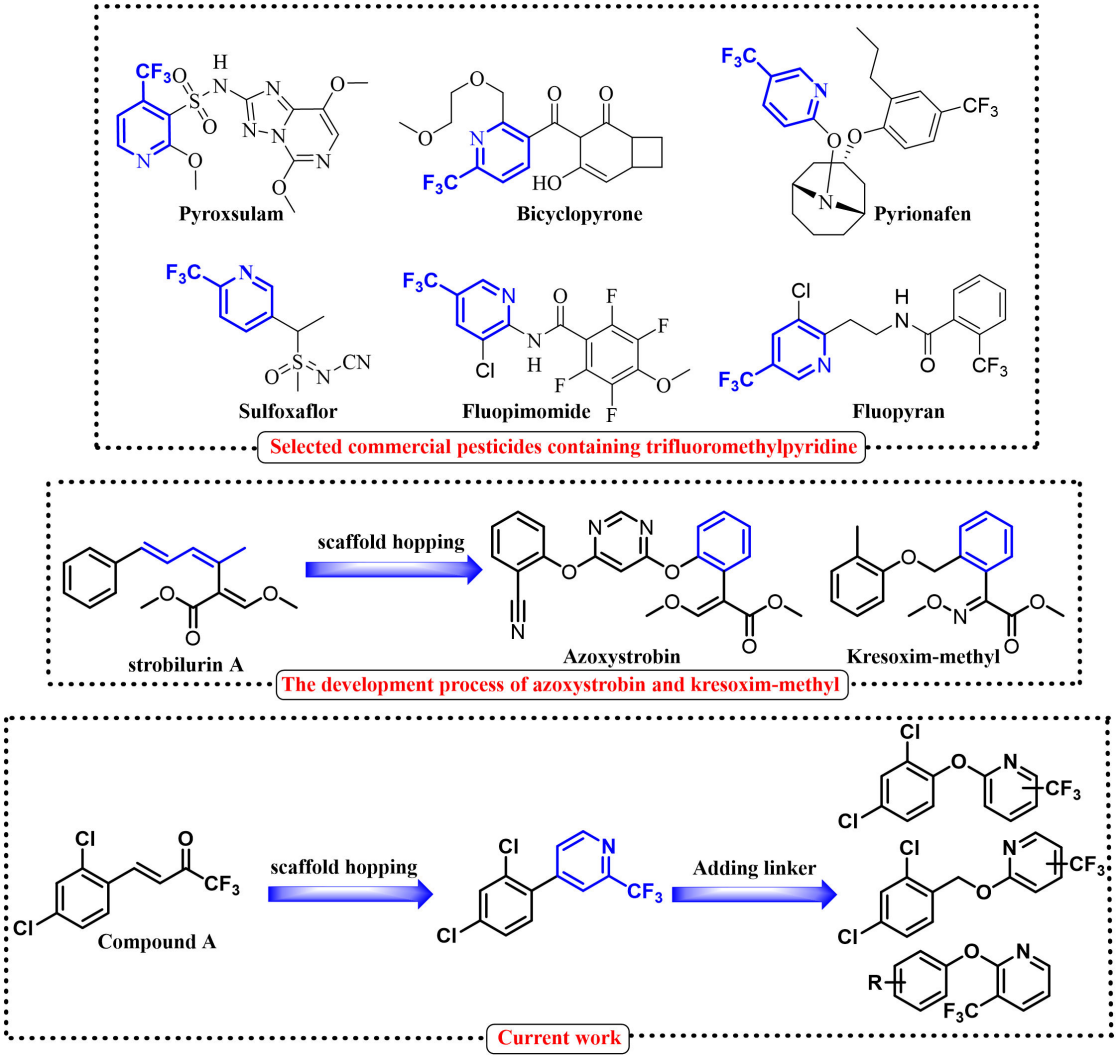
Design strategies for target dehydrozingerone derivatives.

Introducing heterocycles into molecules is a crucial method for optimizing pesticide molecules, and trifluoromethylpyridine is a significant heterocycle used extensively in pesticide chemistry, including in the development of herbicides (pyroxsulam), insecticides (pyrionafen), and fungicides (fluopimomide) (Figure 2) [16,17,18]. Succinate dehydrogenase inhibitors (SDHIs) are an important class of fungicides, with the evolution of three generations to date [19,20]. The development of SDHIs has consistently featured a carboxamide core, with the pyrazole-4-carboxamide moiety emerging as a critical and widespread scaffold, and this structural element has been instrumental in enhancing the antifungal efficacy of the developed compounds (isopyrazam, flubeneteram, inpyrfluxam, Figure 2) against plant pathogens [21,22,23,24,25]. In current research, we are advancing the development of novel pesticides based on dehydrozingerone. Inspired by the development process of azoxystrobin and kresoxim-methyl from strobilurin A (Figure 2) [26], we have employed a scaffold hopping strategy [27,28] to transform the α, β-unsaturated ketones of dehydrozingerone into pyridine rings (Figure 2). To facilitate the synthesis, we have incorporated linking arms into both the benzene and pyridine rings (Figure 2). Following this, we synthesized a series of trifluoromethylpyridine derivatives and evaluated their antifungal potencies. The compounds **1**~**10** were employed to elucidate the impact of the linker and the position of the trifluoromethyl substituent on the pyridine ring on biological activity. Compounds **11**~**17** were utilized to examine the effects of various substituents on the benzene ring on antifungal efficacy, and compounds **18**~**23** were developed for further derivatization. Furthermore, we assessed their stability and probed putative targets of compound **23** using molecular docking.

## 2. Results and Discussion

### 2.1. Chemistry

**Scheme 1:**
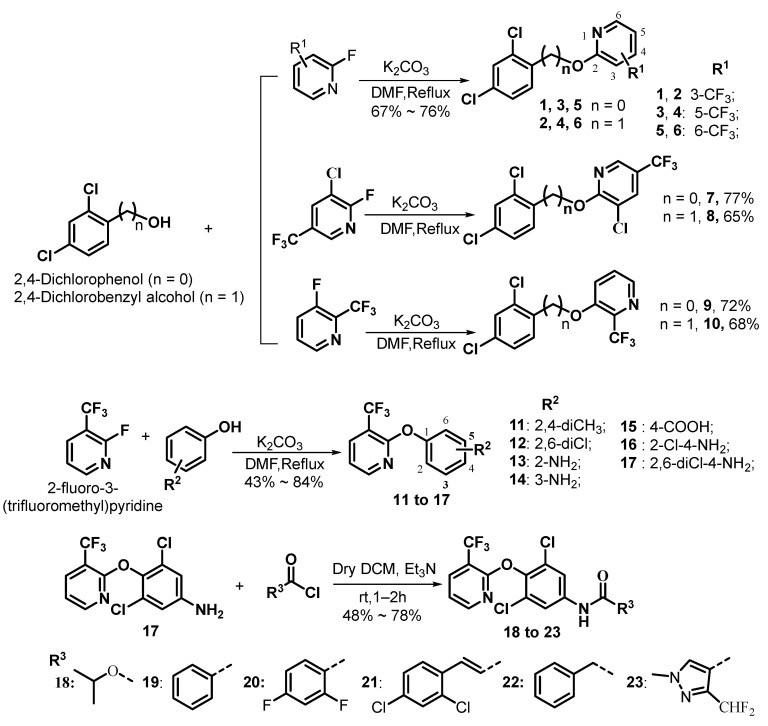
General synthetic route for target compounds.

Previous findings [14] indicated that the 2,4-dichlorophenyl substituent plays a pivotal role in conferring antifungal activity. In our current work, we first designed the molecule that only changed the position of the trifluoromethyl group in the pyridine ring, as well as the position and length of the linker. We successfully synthesized compounds **1** to **10** via nucleophilic aromatic substitution (SNAr) reactions (Scheme 1) [29]. The synthesis utilized 2,4-dichlorophenol and 2,4-dichlorobenzyl alcohol as starting materials, which were reacted with the appropriate fluoropyridine derivatives. Considering the substantial structural deviations of our designed compounds from dehydrozingerone, we also introduced variations in the substituents on the benzene rings. Employing 2-fluoro-3-(trifluoromethyl)pyridine in conjunction with seven diverse substituted phenolic precursors, we prepared an additional series of compounds **11**~**17**, through SNAr reactions (Scheme 1). Furthermore, compound **17** served as a precursor for the synthesis of a subsequent series, **18**~**23**, by reacting it with a range of acyl chlorides (Scheme 1). The chemical structures of the twenty-three compounds were confirmed by nuclear magnetic resonance (NMR) spectroscopy and high-resolution mass spectra, and the corresponding spectra are provided in the Supplementary Information (Figures S1–S69).

### 2.2. In Vitro and In Vivo Antifungal Activity

The in vitro antifungal activity of the target compound was tested against six common plant pathogens (*Rhizoctonia solani*, *Pyricularia oryzae*, *Colletotrichum musae*, *Fusarium graminearum*, *Botrytis cinerea*, and *Colletotrichum siamense*). Evaluation was performed at concentrations of 10 and 50 µg/mL, which were selected to accommodate diverse solubility profiles. The results are presented in Table 1. Subsequent evaluations of compounds with superior activity determined their median effective concentrations (EC_50_), as detailed in Table 2. The data indicate a notable enhancement in antifungal activity for compounds **1**, **3**, **5**, **7,** and **9** compared to compounds **2**, **4**, **6**, **8,** and **10**, suggesting that the presence of a connecting arm with a single oxygen atom positively influences antifungal efficacy. The antifungal activity of compounds **1** and **9** is comparable, and both are more effective than compounds **3**, **5,** and **7**, indicating that the concurrent presence of ether bonds and trifluoromethyl groups at the second and third positions of the pyridine ring in the compound is beneficial for enhancing its antifungal activity. Additionally, the activity of **1** and **12** surpasses that of **11**, and compound **17** shows superior activity over **16**, further confirming that chlorine atom substitution on the benzene ring is beneficial for augmenting antifungal activity. As indicated in Table 2, compound **17** (EC_50_ 2.88~9.09 µg/mL) outperforms both **1** (EC_50_ 8.08~21.96 µg/mL) and **16** (EC_50_ 4.17~52.19 µg/mL) in terms of antifungal activity. The aromatic amino groups in **17** were subjected to amidation, leading to the synthesis of compounds **18**~**23**. Unfortunately, those compounds largely lost their antifungal properties, with only compound **23** maintaining activity comparable to **17**.

To further elucidate the antifungal activity of the compounds, we evaluated the in vivo protective capacity of compounds **17** and **23** against *Colletotrichum musae*. The data are listed in Table 3. At a concentration of 200 μg/mL, the protective effects of compounds **17** and **23** against *C. musae* were nearly comparable, with disease control rates of 46.24% and 48.45%, respectively. These rates are inferior to the in vivo activity exhibited by the positive control compound, azoxystrobin, suggesting that there is scope for enhancing the antifungal potency of these compounds.

### 2.3. Degradation of Compounds ***17*** and ***23*** in Bananas

**Figure 3:**
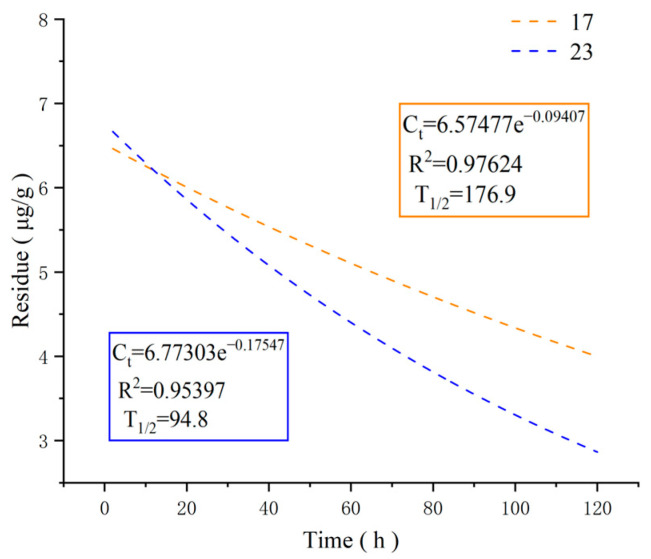
Degradation dynamics of compounds **17** and **23** in banana peels.

In our preliminary studies, we found that compound **A** is photochemically unstable owing to the presence of an α, β-unsaturated ketone, which undergoes complete degradation upon 32 h of light exposure [15]. Despite its potent in vitro antifungal activity, the compound’s efficacy in vivo is significantly reduced. To address this instability, we utilized a scaffold hopping strategy to convert the unsaturated ketone into a pyridine ring, thereby generating a more stable compound. The half-lives of compounds **17** and **23** in bananas were quantified. During the experiment, compounds **17** and **23** were detected exclusively in the peel of the bananas, with no detection in the fruit flesh. The results are presented in Figure 3 (detailed data in Table S2). Compound **17** exhibits a higher degradation rate within the initial 24 h, after which the degradation rate levels off, yielding a half-life of 176.9 h. In contrast, compound **23** degrades more rapidly during the first three days, followed by a plateau in degradation rate, with a half-life of 94.8 h. These results demonstrate that the modification has markedly enhanced the stability of the compounds.

### 2.4. Molecular Docking

**Figure 4:**
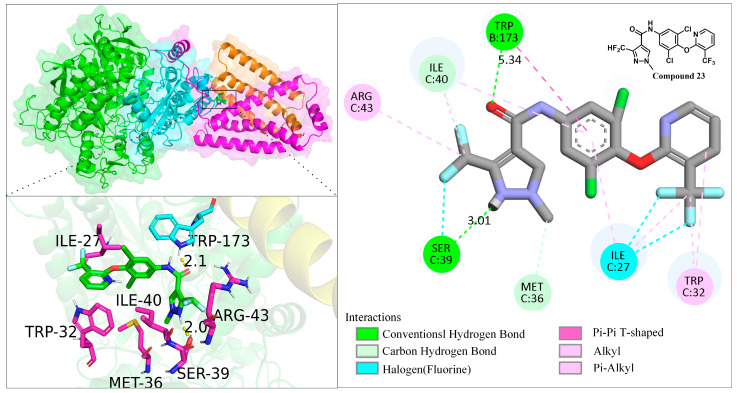
Molecular docking of compound **23**.

**Figure 5:**
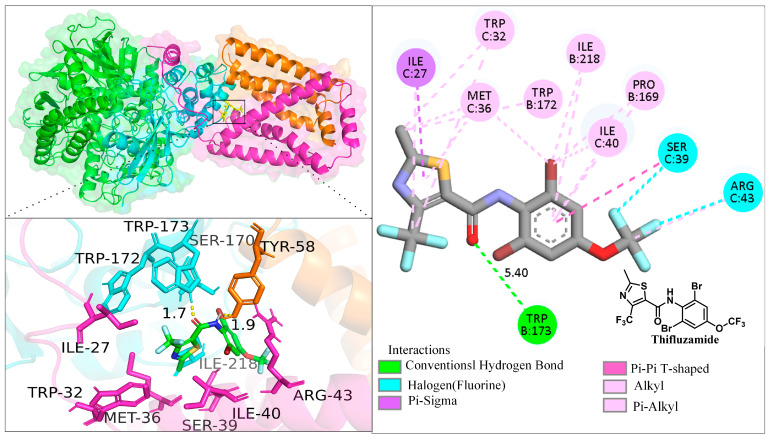
Molecular docking of positive control thifluzamide.

**Figure 6:**
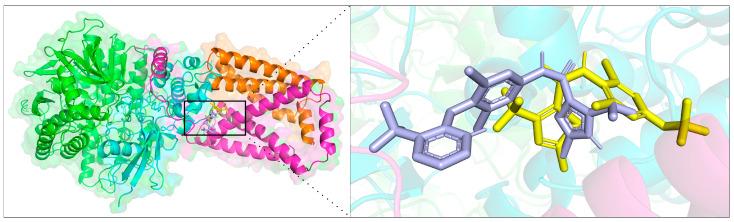
Docking overlap of compound **23** (gray) and thifluzamide (yellow).

Compound structure dictates properties, and compounds with similar structures tend to exhibit similar biological activities and target the same potential sites of action [30,31]. Pyrazole-4-carboxamide is a key functional group known to inhibit succinate dehydrogenase (Figure S70) [20,25], and compound **23** incorporates this group, implying a potential for targeting succinate dehydrogenase. To explore this possibility, we conducted molecular docking experiments to assess the binding interaction between compound **23** and succinate dehydrogenase. We selected thifluzamide, a fungicide known to inhibit succinate dehydrogenase, as the positive control because its structure is similar to that of compound **23**. The molecular docking results are presented in Figure 4 and Figure 5. The succinate dehydrogenase A chain is shown in green, the B chain in cyan, the C chain in light magenta, and the D chain in orange. Compound **23** binds to succinate dehydrogenase with a binding energy of −7.03 kcal/mol, forming hydrogen bonds with the 39th SER amino acid residue of the C chain and the 173rd TRP amino acid residue of the B chain (Figure 4). Thifluzamide binds to succinate dehydrogenase with a binding energy of −6.98 kcal/mol, also forming a hydrogen bond with the 173rd TRP amino acid residue of the B chain (Figure 5). The binding energies of both compounds are comparable. As observed, both compounds share similar binding sites, and Figure 6 provides an overlaid view to facilitate comparison. These results indicate that the target of compound **23** may also be succinate dehydrogenase. Although the above results generated by virtual molecular docking may not perfectly reflect real-world situations, they still offer valuable insights for the molecular optimization of potential inhibitors.

## 3. Materials and Methods

### 3.1. General

Reagents were purchased from commercial sources and used as received. All anhydrous solvents used for synthesis were dried and purified using standard techniques prior to use. The melting points of the synthesized compounds were tested using an X-4 binocular microscope (Beijing Tech Instruments Co., Ltd., Beijing, China). ^1^H NMR and ^13^C NMR spectra were obtained using a Bruker AV 400 spectrometer (Bruker Corp., Fallanden, Switzerland) in CDCl_3_ or DMSO-*d_6_* solutions with tetramethylsilane (TMS) as the internal standard. The progress of the reaction was monitored by thin-layer chromatography on silica gel GF-254 and detected by UV. Column chromatography was performed using 200–300 mesh silica gel (Qingdao Marine Chemical Co., Ltd., Qingdao, China). High-resolution mass spectra were obtained with an Agilent 6210 ESI/TOF MS (Agilent Technologies, Inc., Santa Clara, CA, USA.). High-performance liquid chromatography (HPLC) analyses were conducted using an Agilent 1260 Infinity III instrument (Agilent Technologies, Tokyo, Japan).

### 3.2. Synthesis

General synthesis procedures for title compounds **1**~**17** [32].

A mixture of *N*,*N*-dimethylformamide (DMF, 8 mL) and 2,4-dichlorophenol (10 mmol), 3-trifluoromethyl-2-fluoropyridine (10 mmol), and anhydrous K_2_CO_3_ (15 mmol) was stirred and refluxed for 12 h. The reaction progress was monitored by TLC until completion. After completion of the reaction, the reaction mixture was diluted with water (60 mL) and extracted with ethyl acetate (30 mL × 3). The organic layer was collected and dried over anhydrous sodium sulfate, and the solvent was removed under reduced pressure. The resulting residue was purified by column chromatography on silica gel using a petroleum ether (PE)/ethyl acetate (EA) gradient to obtain the target compounds.

2-(2,4-Dichlorophenoxy)-3-(trifluoromethyl) pyridine (**1**): Rf 0.5 (PE/EA 2:1), White solid, yield 76%, mp 45–46 °C. ^1^H NMR (400 MHz, CDCl_3_) *δ* 8.25 (ddd, *J* = 4.9, 1.9, 0.8 Hz, 1H, 4-H-Py), 8.02 (ddd, *J* = 7.6, 1.8, 0.8 Hz, 1H, 6-H-Py), 7.49 (d, *J* = 2.5 Hz, 1H, 3-H-Ph), 7.32 (dd, *J* = 8.6, 2.5 Hz, 1H, 5-H-Ph), 7.19 (d, *J* = 8.6 Hz, 1H, 6-H-Ph), 7.12 (ddd, *J* = 7.6, 5.0, 0.9 Hz, 1H, 5-H-Py). ^13^C NMR (100 MHz, CDCl_3_) *δ* 159.3 (q, *J* = 1.8 Hz), 150.7, 147.7, 137.3 (q, *J* = 4.7 Hz), 131.6, 130.4, 128.4, 128.1, 125.0, 122.6 (q, *J* = 272.1 Hz), 118.3, 113.8 (q, *J* = 34.0 Hz). HRMS (ESI) *m*/*z* calculated for C_12_H_7_NOF_3_Cl_2_^+^ [M + H]^+^, 307.9851; found, 307.9855.

2-((2,4-Dichlorobenzyl)oxy)-3-(trifluoromethyl) pyridine (**2**): Rf 0.45 (PE/EA 2:1), White solid, yield 71%, mp 74–75 °C. ^1^H NMR (400 MHz, CDCl_3_) *δ* 8.33 (dd, *J* = 5.1, 1.8 Hz, 1H, 4-H-Py), 7.90 (dd, *J* = 7.6, 1.9 Hz, 1H, 6-H-Py), 7.52 (d, *J* = 8.3 Hz, 1H, 6-H-Ph), 7.40 (d, *J* = 2.1 Hz, 1H, 3-H-Ph), 7.26 (dd, *J* = 8.2, 2.0 Hz, 1H, 5-H-Ph), 7.01 (dd, *J* = 7.5, 5.0 Hz, 1H, 5-H-Py), 5.55 (s, 2H, CH_2_). ^13^C NMR (100 MHz, CDCl_3_) *δ* 159.7 (q, *J* = 1.7 Hz), 150.5, 136.6 (q, *J* = 4.8 Hz), 133.9, 133.2, 129.3, 129.1, 127.3, 123.0 (q, *J* = 271.8 Hz), 116.6, 113.4 (q, *J* = 33.2 Hz), 64.72. HRMS (ESI) *m*/*z* calculated for C_13_H_9_NOF_3_Cl_2_^+^ [M + H]^+^, 322.0008; found, 322.0014.

2-(2,4-Dichlorophenoxy)-5-(trifluoromethyl)pyridine (**3**): Rf 0.45 (PE/EA 2:1), Light yellow liquid, yield 68%. ^1^H NMR (400 MHz, CDCl_3_) *δ* 8.41–8.36 (m, 1H, 6-H-Py), 7.94 (dd, *J* = 8.6, 2.5 Hz, 1H, 4-H-Py), 7.50 (d, *J* = 2.5 Hz, 1H, 3-H-Ph), 7.32 (dd, *J* = 8.7, 2.5 Hz, 1H, 5-H-Ph), 7.17 (d, *J* = 8.6 Hz, 1H, 6-H-Ph), 7.12 (dt, *J* = 8.7, 0.7 Hz, 1H, 3-H-Py). ^13^C NMR (100 MHz, CDCl_3_) *δ* 164.6, 147.8, 145.3 (q, *J* = 4.4 Hz), 137.0 (q, *J* = 3.3 Hz), 131.7, 130.5, 128.3, 128.2, 124.8, 123.6 (q, *J* = 270.0 Hz), 122.1 (q, *J* = 33.0 Hz), 111.2. HRMS (ESI) *m*/*z* calculated for C_12_H_7_NOF_3_Cl_2_^+^ [M + H]^+^, 307.9851; found, 307.9860.

2-((2,4-Dichlorobenzyl)oxy)-5-(trifluoromethyl) pyridine (**4**)**:** Rf 0.45 (PE/EA 2:1), White solid, yield 67%, mp 63–64 °C. ^1^H NMR (400 MHz, CDCl_3_) *δ* 8.46 (dt, *J* = 2.8, 1.0 Hz, 1H, 6-H-Py), 7.81 (dd, *J* = 8.7, 2.5 Hz, 1H, 4-H-Py), 7.46 (d, *J* = 8.3 Hz, 1H, 6-H-Ph), 7.43 (d, *J* = 2.1 Hz, 1H, 3-H-Ph), 7.26 (dd, *J* = 8.3, 2.1 Hz, 1H, 5-H-Ph), 6.91 (dt, *J* = 8.6, 0.8 Hz, 1H, 3-H-Py), 5.49 (s, 2H, CH_2_). ^13^C NMR (100 MHz, CDCl_3_) *δ* 165.1, 145.0 (q, *J* = 4.4 Hz), 136.0 (q, *J* = 3.2 Hz), 134.4, 134.1, 133.0, 130.4, 129.4, 127.2, 123.9 (q, *J* = 270.8 Hz), 120.6 (q, *J* = 33.1 Hz), 111.3, 65.0. HRMS (ESI) *m*/*z* calculated for C_13_H_9_NOF_3_Cl_2_^+^ [M + H]^+^, 322.0008; found, 322.0016.

2-(2,4-Dichlorophenoxy)-6-(trifluoromethyl) pyridine (**5**): Rf 0.5 (PE/EA 2:1), colorless liquid, yield 68%. ^1^H NMR (400 MHz, CDCl_3_) *δ* 7.91–7.83 (m, 1H, 4-H-Py), 7.48 (d, *J* = 2.4 Hz, 1H, 3-H-Ph), 7.40 (d, *J* = 7.4 Hz, 1H, 3-H-Py), 7.29 (dd, *J* = 8.7, 2.5 Hz, 1H, 5-H-Ph), 7.20 (d, *J* = 8.7 Hz, 1H, 6-H-Ph), 7.15 (d, *J* = 8.3 Hz, 1H, 5-H-Py). ^13^C NMR (100 MHz, CDCl_3_) *δ* 162.4, 147.9, 146.1 (q, *J* = 35.5 Hz), 140.8, 131.2, 130.4, 128.1, 128.1, 124.7, 120.9 (q, *J* = 274.0 Hz), 115.4 (q, *J* = 3.1 Hz), 114.4 (q, *J* = 1.2 Hz). HRMS (ESI) *m*/*z* calculated for C_12_H_7_NOF_3_Cl_2_^+^ [M + H]^+^, 307.9851; found, 307.9858.

2-((2,4-Dichlorobenzyl)oxy)-6-(trifluoromethyl) pyridine (**6**): Rf 0.55 (PE/EA 2:1), White solid, yield 74%, mp 54–55 °C. ^1^H NMR (400 MHz, CDCl_3_) *δ* 7.73 (t, *J* = 7.9 Hz, 1H, 4-H-Py), 7.53 (d, *J* = 8.3 Hz, 1H, 6-H-Ph), 7.42 (d, *J* = 2.1 Hz, 1H, 3-H-Ph), 7.33–7.22 (m, 2H, 5-H-Ph and 3-H-Py), 6.98 (d, *J* = 8.4 Hz, 1H, 5-H-Py), 5.49 (s, 2H, CH_2_). ^13^C NMR (100 MHz, CDCl_3_) *δ* 163.1, 145.5 (q, *J* = 34.7 Hz), 139.7, 134.8, 134.6, 133.0, 131.5, 129.4, 127.1, 121.3 (q, *J* = 274.8 Hz), 114.8 (q, *J* = 1.2 Hz), 113.8 (q, *J* = 3.2 Hz), 64.9. HRMS (ESI) *m*/*z* calculated for C_13_H_9_NOF_3_Cl_2_^+^ [M + H]^+^, 322.0008; found, 322.0014.

3-Chloro-2-(2,4-dichlorophenoxy)-5-(trifluoromethyl) pyridine (**7**): Rf 0.5 (PE/EA 2:1), colorless liquid, yield 77%. ^1^H NMR (400 MHz, CDCl_3_) *δ* 8.24 (dt, *J* = 2.2, 1.0 Hz, 1H, 4-H-Py), 8.01 (d, *J* = 2.2 Hz, 1H, 6-H-Py), 7.51 (d, *J* = 2.5 Hz, 1H, 3-H-Ph), 7.34 (dd, *J* = 8.7, 2.5 Hz, 1H, 5-H-Ph), 7.19 (d, *J* = 8.7 Hz, 1H, 6-H-Ph). ^13^C NMR (100 MHz, CDCl_3_) *δ* 160.1, 147.6, 142.5 (q, *J* = 4.4 Hz), 136.7 (q, *J* = 3.4 Hz), 132.1, 130.5, 128.3, 128.2, 124.8, 123.1 (q, *J* = 33.8 Hz), 122.7 (q, *J* = 272.3 Hz), 119.0. HRMS (ESI) *m*/*z* calculated for C_12_H_6_NOF_3_Cl_3_^+^ [M + H]^+^, 341.9462; found, 341.9470.

3-Chloro-2-((2,4-dichlorobenzyl)oxy)-5-(trifluoromethyl) pyridine (**8**): Rf 0.5 (PE/EA 2:1), White solid, yield 65%, mp 138–139 °C. ^1^H NMR (400 MHz, CDCl_3_) *δ* 7.80 (dq, *J* = 2.6, 1.3 Hz, 1H, 4-H-Py), 7.67 (d, *J* = 2.5 Hz, 1H, 3-H-Ph), 7.50–7.44 (m, 2H, 5-H-Ph and 6-H-Py), 7.29–7.26 (m, 1H, 6-H-Ph), 5.26 (s, 2H, CH_2_). ^13^C NMR (100 MHz, CDCl_3_) *δ* 158.5, 135.7, 135.5 (q, *J* = 5.4 Hz), 134.5, 133.3 (q, *J* = 2.5 Hz), 132.7, 130.4, 129.8, 127.9, 127.8, 122.6 (q, *J* = 269.7 Hz), 109.5 (q, *J* = 35.8 Hz), 51.4. HRMS (ESI) *m*/*z* calculated for C_13_H_7_NOF_3_Cl_3_Na^+^ [M + Na]^+^, 377.9438; found, 377.9442.

3-(2,4-Dichlorophenoxy)-2-(trifluoromethyl) pyridine (**9**): Rf 0.5 (PE/EA 2:1), Dark yellow liquid, yield 72%. ^1^H NMR (400 MHz, CDCl_3_) *δ* 8.43 (dd, *J* = 4.7, 1.2 Hz, 1H, 6-H-Py), 7.53 (d, *J* = 2.5 Hz, 1H, 3-H-Ph), 7.43 (dd, *J* = 8.5, 4.6 Hz, 1H, 5-H-Py), 7.30 (dd, *J* = 8.7, 2.5 Hz, 1H, 5-H-Ph), 7.11 (dd, *J* = 8.5, 1.2 Hz, 1H, 4-H-Py), 7.04 (d, *J* = 8.7 Hz, 1H, 6-H-Ph). ^13^C NMR (100 MHz, CDCl_3_) *δ* 151.9, 149.0, 143.2, 137.8 (q, *J* = 34.7 Hz), 131.6, 131.0, 128. 7, 127.7, 127.6, 124.7, 122.9, 121.4 (q, *J* = 274.8 Hz). HRMS (ESI) *m*/*z* calculated for C_12_H_7_NOF_3_Cl_2_^+^ [M + H]^+^, 307.9851; found, 307.9858.

3-((2,4-Dichlorobenzyl)oxy)-2-(trifluoromethyl) pyridine (**10**): Rf 0.5 (PE/EA 2:1), White solid, yield 68%, mp 117–118 °C. ^1^H NMR (400 MHz, CDCl_3_) *δ* 8.32 (dd, *J* = 4.5, 1.3 Hz, 1H, 6-H-Py), 7.55 (dd, *J* = 8.4, 1.0 Hz, 1H, 4-H-Py), 7.48 (dd, *J* = 8.5, 4.5 Hz, 1H, 5-H-Py), 7.45–7.40 (m, 2H, 3-H-Ph and 6-H-Ph), 7.33 (dd, *J* = 8.4, 2.1 Hz, 1H, 5-H-Ph), 5.25 (s, 2H, CH_2_). ^13^C NMR (100 MHz, CDCl_3_) *δ* 152.8, 141.0, 137.1 (q, *J* = 34.09 Hz), 134.6, 132.6, 131.8, 129.3, 129.1, 127.8, 127.7, 121.7 (q, *J* = 274.5 Hz), 121.2, 67.0. HRMS (ESI) *m*/*z* calculated for C_13_H_9_NOF_3_Cl_2_^+^ [M + H]^+^, 322.0008; found, 322.0015.

2-(2,4-Dimethylphenoxy)-3-(trifluoromethyl) pyridine (**11**): Rf 0.5 (PE/EA 2:1), Pale yellow solid, yield 70%, mp 62–63 °C. ^1^H NMR (400 MHz, CDCl_3_) *δ* 8.26 (ddd, *J* = 5.0, 1.8, 0.8 Hz, 1H, 4-H-Py), 7.97 (ddd, *J* = 7.5, 1.9, 0.8 Hz, 1H, 6-H-Py), 7.11–7.08 (m, 1H, 5-H-Py), 7.07–7.01 (m, 2H, 3-H-Ph and 5-H-Ph), 6.98 (d, *J* = 8.1 Hz, 1H, 6-H-Ph), 2.34 (s, 3H, CH_3_), 2.11 (s, 3H, CH_3_). ^13^C NMR (100 MHz, CDCl_3_) *δ* 160.4, 151.0, 149.0, 137.0 (q, *J* = 4.7 Hz), 135.3, 132.0, 130.3, 127.6, 123.0 (q, *J* = 271.9 Hz), 121.9, 117.1, 113.6 (q, *J* = 33.4 Hz), 20.9, 16.2. HRMS (ESI) *m*/*z* calculated for C_14_H_13_NOF_3_^+^ [M + H]^+^, 268.0944, found; 268.0945.

2-(2,6-Dichlorophenoxy)-3-(trifluoromethyl) pyridine (**12**): Rf 0.5 (PE/EA 2:1), White solid, yield 51%, mp 96–97 °C. ^1^H NMR (400 MHz, CDCl_3_) *δ* 8.23 (ddd, *J* = 5.0, 1.8, 0.8 Hz, 1H, 4-H-Py), 8.03 (ddd, *J* = 7.5, 1.8, 0.8 Hz, 1H, 6-H-Py), 7.40 (d, *J* = 8.1 Hz, 2H, 3-H-Ph and 5-H-Ph), 7.18 (dd, *J* = 8.5, 7.8 Hz, 1H, 4-H-Ph), 7.13 (ddt, *J* = 7.5, 5.0, 0.8 Hz, 1H, 5-H-Py). ^13^C NMR (100 MHz, CDCl_3_) *δ* 158.40 (q, *J* = 1.7 Hz), 150.7, 145.6, 137.31 (q, *J* = 4.7 Hz), 129.6, 128.8, 126.9, 122.7 (q, *J* = 272.2 Hz), 118.3, 113.5 (q, *J* = 34.2 Hz). HRMS (ESI) *m*/*z* calculated for C_12_H_7_NOF_3_Cl_2_^+^ [M + H]^+^, 307.9851; found, 307.9854.

2-((3-(Trifluoromethyl)pyridin-2-yl)oxy)aniline (**13**): Rf 0.5 (PE/EA 1:1), Light pink solid, yield 84%, mp 73–74 °C. ^1^H NMR (400 MHz, CDCl_3_) *δ* (dd, *J* = 5.1, 1.8 Hz, 1H, 4-H-Pyr), 7.99 (dd, *J* = 7.5, 1.9 Hz, 1H, 6-H-Py), 7.10 (d, *J* = 1.5 Hz, 1H, Ph), 7.09–7.06 (m, 2H, Ph), 6.87 (dd, *J* = 8.3, 1.6 Hz, 1H, Ph), 6.85–6.79 (m, 1H, 5-H-Py), 3.37 (br, 2H, NH_2_). ^13^C NMR (100 MHz, CDCl_3_) *δ* 159.9 (q, *J* = 1.9 Hz), 151.2, 140.1, 138.8, 137.0 (q, *J* = 4.7 Hz), 126.5, 123.0 (q, *J* = 272.0 Hz), 122.82, 118.9, 117.8, 117.0, 113.9 (q, *J* = 33.1 Hz). HRMS (ESI) *m*/*z* calculated for C_12_H_10_N_2_OF_3_^+^ [M + H]^+^, 255.0740; found, 255.0743.

3-((3-(Trifluoromethyl)pyridin-2-yl)oxy)aniline (**14**): Rf 0.5 (PE/EA 1:1), White solid, yield 79%, mp 75–76 °C. ^1^H NMR (400 MHz, CDCl_3_) *δ* 8.31 (ddd, *J* = 5.0, 1.9, 0.8 Hz, 1H, 4-H-Py), 7.97 (ddd, *J* = 7.5, 1.9, 0.8 Hz, 1H, 6-H-Py), 7.18 (t, *J* = 8.0 Hz, 1H, Ph), 7.07 (ddd, *J* = 7.6, 4.9, 0.8 Hz, 1H, 5-H-Py), 6.58–6.51 (m, 2H, Ph), 6.49 (t, *J* = 2.2 Hz, 1H, Ph), 3.37 (s, 2H, NH_2_). ^13^C NMR (100 MHz, CDCl_3_) *δ* 160.5, 154.2, 151.0, 147.8, 137.0 (q, *J* = 4.7 Hz), 130.2, 122.8 (q, *J* = 271.9 Hz), 117.6, 114.4 (q, *J* = 33.4 Hz), 112.3, 111.6, 108.4. HRMS (ESI) *m*/*z* calculated for C_12_H_10_N_2_OF_3_^+^ [M + H]^+^, 255.0740; found, 255.0740.

4-((3-(Trifluoromethyl)pyridin-2-yl)oxy)benzoic acid (**15**): Rf 0.45 (PE/EA 1:1), White solid, yield 43%, mp 157–158 °C. ^1^H NMR (400 MHz, DMSO-*d_6_*) *δ* 8.43 (dd, *J* = 5.0, 1.8 Hz, 1H, 4-H-Py), 8.31 (dd, *J* = 7.7, 1.8 Hz, 1H, 6-H-Py), 8.01 (d, *J* = 7.8 Hz, 2H, 2-H-Ph and 6-H-Ph), 7.39 (dd, *J* = 7.7, 4.9 Hz, 1H, 5-H-Py), 7.27 (d, *J* = 7.0 Hz, 2H, 3-H-Ph and 5-H-Ph). ^13^C NMR (100 MHz, DMSO-*d_6_*) *δ* 159.4, 1568, 152.2, 138.6 (q, *J* = 4.6 Hz), 131.5, 124.7, 122.0, 121.6, 120.0, 113.5 (q, *J* = 32.9 Hz). HRMS (ESI) *m*/*z* calculated for C_13_H_7_NO_3_F_3_^−^ [M-H]^−^, 282.0384; found, 282.0389.

3-Chloro-4-((3-(trifluoromethyl)pyridin-2-yl)oxy)aniline (**16**): Rf 0.5 (PE/EA 2:1),Brick red solid, yield 63%, mp 113–114 °C. ^1^H NMR (400 MHz, CDCl_3_) *δ* 8.26 (ddd, *J* = 5.0, 1.9, 0.8 Hz, 1H, 4-H-Py), 7.98 (ddd, *J* = 7.6, 1.9, 0.8 Hz, 1H, 6-H-Py), 7.06 (ddd, *J* = 7.6, 5.0, 0.8 Hz, 1H, 5-H-Py), 7.02 (d, *J* = 8.6 Hz, 1H, 6- H-Ph), 6.78 (d, *J* = 2.7 Hz, 1H, 3- H-Ph), 6.61 (dd, *J* = 8.6, 2.7 Hz, 1H, 5- H-Ph), 3.5 (br, 2H, NH_2_). ^13^C NMR (100 MHz, CDCl_3_) *δ* 160.2, 150.8, 145.1, 140.7, 137.0 (q, *J* = 4.7 Hz), 127.7, 124.5, 122.8 (q, *J* = 271.9 Hz), 117.6, 116.4, 114.4, 113.6 (q, *J* = 33.8 Hz). HRMS (ESI) *m*/*z* calculated for C_12_H_9_N_2_OF_3_Cl^+^ [M + H]^+^, 289.0350; found, 289.0351.

3,5-Dichloro-4-((3-(trifluoromethyl)pyridin-2-yl)oxy)aniline (**17**): Rf 0.5 (PE/EA 2:1), White solid, yield 56%, mp 142–143 °C. ^1^H NMR (400 MHz, CDCl_3_) *δ* 8.25 (ddd, *J* = 5.0, 1.9, 0.8 Hz, 1H, 4-H-Py), 8.00 (ddd, *J* = 7.6, 1.9, 0.8 Hz, 1H, 6-H-Py), 7.10 (ddd, *J* = 7.6, 5.0, 0.8 Hz, 1H, 5-H-Py), 6.67 (s, 2H, Ph), 3.52 (s, 2H, NH_2_) ^13^C NMR (100 MHz, CDCl_3_) *δ* 159.0 (q, *J* = 1.6 Hz), 150.7, 145.1, 137.2 (q, *J* = 4.7 Hz), 137.0, 129.5, 122.7 (q, *J* = 272.2 Hz), 118.1, 114.8, 113.4 (q, *J* = 34.1 Hz). HRMS (ESI) *m*/*z* calculated for C_12_H_8_N_2_OF_3_Cl_2_^+^ [M + H]^+^, 322.9960; found, 322.9965.

General Synthesis Procedures for Title Compounds **18**~**23**.

Compound **17** (1 mmol) and Et_3_N (4 mmol) were added to dry DCM (10 mL) and stirred, followed by the dropwise addition of an excess of acyl chloride (5 mmol) via a dropping funnel. The mixture was stirred at room temperature for 2 h, then diluted with additional DCM (20 mL) and washed with a saturated NaHCO_3_ solution (5 mL). The organic layer was then evaporated under reduced pressure. The residue was purified by flash column chromatography on silica gel using a PE/EA gradient, affording compounds **18**~**23**.

Isopropyl(3,5-dichloro-4-((3-(trifluoromethyl)pyridin-2-yl)oxy)phenyl)carbamate (**18**): Rf 0.5 (PE/EA 2:1),Yellow solid, yield 73%, mp 127–128 °C. ^1^H NMR (400 MHz, DMSO-*d*_6_) *δ* 10.03 (s, 1H, NH), 8.37 (dd, *J* = 5.0, 1.8 Hz, 1H, 4-H-Py), 8.33 (dd, *J* = 7.7, 1.8 Hz, 1H, 6-H-Py), 7.68 (s, 2H, Ph), 7.39 (dd, *J* = 7.6, 5.0 Hz, 1H, 5-H-Py), 4.92 (hept, *J* = 6.2 Hz, 1H, CH), 1.28 (d, *J* = 6.2 Hz, 6H, CH_3_). ^13^C NMR (100 MHz, DMSO-*d*_6_) *δ* 158.1, 153.5, 151.9, 139.5, 134.0, 138.8 (q, *J* = 4.8 Hz), 128.7, 123.3 (q, *J* = 272.0 Hz), 120.0, 118.27, 112.0 (q, *J* = 33.7 Hz), 68.9, 22.3. HRMS (ESI) m/z calculated for C_16_H_12_N_2_O_3_F_3_Cl_2_^−^ [M-H]^−^, 407.0183; found, 407.0191.

*N*-(3,5-dichloro-4-((3-(trifluoromethyl)pyridin-2-yl)oxy)phenyl)benzamide (**19**): Rf 0.45 (PE/EA 2:1), White solid, yield 78%, mp 175–176 °C. ^1^H NMR (400 MHz, CDCl_3_) *δ* 8.27–8.20 (m, 1H, 4-H-Py), 8.07–7.99 (m, 1H, 6-H-Py), 7.90 (s, 1H, NH), 7.88–7.83 (m, 2H, Ph), 7.80 (s, 2H, Ph), 7.63–7.55 (m, 1H, Ph), 7.55–7.46 (m, 2H, Ph), 7.18–7.10 (m, 1H, 5-H-Py). ^13^C NMR (100 MHz, CDCl_3_) *δ* 165.8, 158.5, 150.7, 141.9, 137.3 (q, *J* = 4.6 Hz), 136.4, 134.1, 132.4, 129.7, 129.0, 127.1, 122.7 (q, *J* = 272.1 Hz), 120.1, 118.4, 113.5 (q, *J* = 34.3 Hz). HRMS (ESI) *m*/*z* calculated for C_19_H_11_N_2_O_2_F_3_Cl_2_Na^+^ [M + Na]^+^, 449.0042; found, 449.0043.

*N*-(3,5-dichloro-4-((3-(trifluoromethyl)pyridin-2-yl)oxy)phenyl)-2,4-difluorobenzamide (**20**): Rf 0.45 (PE/EA 2:1), Beige solid, yield 72%, mp 176–177 °C. ^1^H NMR (400 MHz, CDCl_3_) *δ* 8.37 (d, *J* = 16.0 Hz, 1H, Ph), 8.27–8.17 (m, 2H, Ph and NH), 8.06–8.02 (m, 1H, Py), 7.80 (s, 2H, Ph), 7.17–7.12 (m, 1H, Py), 7.12–7.06 (m, 1H, Py), 6.97 (ddd, *J* = 12.3, 8.3, 2.4 Hz, 1H, Ph). ^13^C NMR (100 MHz, CDCl_3_) *δ* 165.4 (dd, *J* = 257.3, 13.2 Hz), 160.8 (dd, *J* = 248.6, 12.4 Hz), 160.4 (d, *J* = 4.0 Hz), 158.5, 150.7, 142.2, 137.3 (q, *J* = 4.7 Hz), 135.9, 134.2 (dd, *J* = 10.4, 3.5 Hz), 129.8, 122.6 (q, *J* = 272.2 Hz) 120.5, 118.4, 117.1 (dd, *J* = 11.4, 3.8 Hz), 113.5 (q, *J* = 34.2 Hz), 113.1 (dd, *J* = 21.4, 3.2 Hz), 104.6 (dd, *J* = 29.1, 26.0 Hz). HRMS (ESI) *m*/*z* calculated for C_19_H_9_N_2_O_2_F_5_Cl_2_Na^+^ [M + Na]^+^, 484.9853; found, 484.9861.

*N*-(3,5-dichloro-4-((3-(trifluoromethyl)pyridin-2-yl)oxy)phenyl)-3-(2,4-dichlorophenyl)acrylamide (**21**): Rf 0.45 (PE/EA 2:1), White solid, yield 61%, mp 250–251 °C. ^1^H NMR (400 MHz, DMSO-*d*_6_) *δ* 10.75 (s, 1H, NH), 8.39 (dd, *J* = 5.0, 1.7 Hz, 1H, 4-H-Py), 8.35 (dd, *J* = 7.7, 1.8 Hz, 1H, 6-H-Py), 7.93 (s, 2H, Ph), 7.90–7.81 (m, 2H, CH and 6-H-Ph), 7.78 (d, *J* = 2.2 Hz, 1H, 3-H-Ph), 7.56 (dd, *J* = 8.5, 2.2 Hz, 1H, 5-H-Ph), 7.40 (dd, *J* = 7.6, 5.0 Hz, 1H, 5-H-Py), 6.85 (d, *J* = 15.6 Hz, 1H, CH). ^13^C NMR (100 MHz, DMSO-*d*_6_) *δ* 163.8, 158.1, 151.9, 140.6, 138.9, 138.4, 135.8, 135.6, 134.9, 131.7, 130.1, 129.6, 128.8, 128.6, 125.5, 123.2 (q, *J* = 271.9 Hz), 120.1, 119.7, 112.1 (q, *J* = 33.9 Hz). HRMS (ESI) *m*/*z* calculated for C_21_H_11_N_2_O_2_F_3_Cl_4_Na^+^ [M + Na]^+^, 542.9419; found, 542.9423.

*N*-(3,5-dichloro-4-((3-(trifluoromethyl)pyridin-2-yl)oxy)phenyl)-2-phenylacetamide (**22**): Rf 0.45 (PE/EA 2:1), Pale yellow solid, yield 48%, mp over 300 °C. ^1^H NMR (400 MHz, CDCl_3_) *δ* 8.19 (dd, *J* = 5.1, 1.8 Hz, 1H, 4-H-Py), 8.01 (ddd, *J* = 7.5, 1.8, 0.8 Hz, 1H, 6-H-Py), 7.55 (s, 2H, Ph), 7.46–7.40 (m, 2H, Ph), 7.40–7.34 (m, 1H, Ph), 7.34–7.30 (m, 2H, Ph), 7.12 (dd, *J* = 7.6, 5.0 Hz, 1H, 5-H-Py), 7.08 (s, 1H, NH), 3.75 (s, 2H, CH_2_). ^13^C NMR (100 MHz, CDCl_3_) *δ* 169.2, 158.5, 150.6, 141.8, 137.3 (q, *J* = 4.6 Hz), 136.1, 133.8, 129.6, 129.5, 129.4, 128.0, 122.6 (q, *J* = 272.2 Hz), 119.6, 118.4, 113.5 (q, *J* = 34.3 Hz), 44.8. HRMS (ESI) *m*/*z* calculated for C_20_H_12_N_2_O_2_F_3_Cl_2_^−^ [M-H]^−^, 439.0233; found, 439.0217.

*N*-(3,5-dichloro-4-((3-(trifluoromethyl)pyridin-2-yl)oxy)phenyl)-3-(difluoromethyl)-1-methyl-1H-pyrazole-4-carboxamide (**23**): Rf 0.5 (PE/EA 2:1), Pale yellow solid, yield 76%, mp 158–159 °C. ^1^H NMR (400 MHz, CDCl_3_) *δ* 8.23 (dd, *J* = 5.1, 1.8 Hz, 1H, 4-H-Py), 8.18 (t, *J* = 5.8 Hz, 1H, H-Pyr), 8.05 (s, 1H, NH), 8.03 (dd, *J* = 7.4, 1.8 Hz, 1H, 6-H-Py), 7.74 (s, 2H, Ph), 7.13 (dd, *J* = 7.6, 5.0 Hz, 1H, 5-H-Py), 6.88 (t, *J* = 54.2 Hz, 1H, CH), 3.97 (s, 3H, CH_3_). ^13^C NMR (100 MHz, CDCl_3_) *δ* 159.1, 158.5 (q, *J* = 1.6 Hz), 150.7, 142.4 (t, *J* = 29.3 Hz), 141.9, 137.3 (q, *J* = 4.8 Hz), 136.3, 136.2, 129.7, 122.7 (q, *J* = 272.0 Hz), 112.0, 118.4, 116.5, 113.5 (q, *J* = 34.3 Hz), 112.2 (t, *J* = 232.8 Hz), 39.7. HRMS (ESI) *m*/*z* calculated for C_18_H_10_N_4_O_2_F_5_Cl_2_^−^ [M-H]^−^, 479.0106; found, 479.0105.

### 3.3. Biological Assays

Antifungal effects of title compounds in vitro [33,34].

The antifungal activity of a series of target compounds was evaluated in vitro against six plant pathogenic fungi, *Rhizoctonia solani*, *Pyricularia oryzae*, *Colletotrichum musae*, *Fusarium graminearum*, *Botrytis cinerea*, and *Colletotrichum siamense*, using the mycelial growth rate method. The plant pathogens were collected from the field and identified with morphology and molecular phylogenetic analysis by Professor Changping Xie from the School of Tropical Agriculture and Forestry at Hainan University and stored in the Key Laboratory of Green Prevention and Control of Tropical Plant Diseases and Pests, Ministry of Education. All six of the plant pathogenic fungi had good infecting ability. The target compounds were prepared as a solution in DMF and then incorporated into the potato dextrose agar (PDA) medium, maintaining a consistent concentration of 10 or 50 μg/mL in the medium. Subsequently, 5 mm mycelial disks were placed at the center of the PDA plates (with three plates per treatment) and maintained at a temperature of 27 ± 1 °C for a period ranging from 4 to 7 days. The experimental setup was conducted in triplicate for each compound. DMF without compounds served as the negative control, while pyrimethanil and azoxystrobin were applied as the reference standards. Subsequently, the toxic regression equations and median effective concentrations (EC_50_) of some active compounds were determined. The test compounds were diluted to five distinct concentrations, and the inhibition percentage for each concentration was quantified. The logarithmic transformation of these concentrations and their associated inhibition percentages were then employed to conduct a linear regression, which yielded the EC_50_ value. The inhibition of the title compound against these fungi was calculated by the following formula:

$$I=\frac{C-T}{C-0.5}\;\times \;100$$

I represents the inhibition rate, C represents the diameter of fungal growth on untreated PDA, and T represents the diameter of fungi on treated PDA.

In vivo protective activity against *C. musae* on bananas [35].

The freshly purchased bananas were washed with water and disinfected with 75% ethanol, then rinsed with distilled water for later use. The compound was dissolved in 0.2 mL of dimethyl sulfoxide (DMSO) and diluted with 0.2% Triton-100 in water to a final concentration of 200 µg/mL test solution. This solution was evenly sprayed onto the bananas. After 24 h, a spore suspension of *C. musae* at a concentration of 5–6 × 10^8^ CFU/mL was applied to the bananas. Solvents were used as the negative control, and azoxystrobin served as the positive control. The treated bananas were then incubated at 25 °C and 80% relative humidity, and the incidence of disease was assessed 5–7 days later. The grading criteria are as follows: Grade 0: No disease spots. Level 1: The lesion area is less than 5% of the fruit area. Level 3: Disease spot area accounts for 6% to 10% of the fruit area. Level 5: Disease spot area accounts for 11% to 25% of the fruit area. Level 7: Disease spot area accounts for 26% to 50% of the fruit area. Level 9: The lesion area accounts for more than 50% of the fruit area.

$$\mathrm{Disease}\;\mathrm{index}=\frac{\sum \left(\mathrm{incidence}\;\mathrm{number}\;\mathrm{of}\;\mathrm{all}\;\mathrm{levels}\;\text{×}\;\mathrm{representative}\;\mathrm{value}\;\mathrm{of}\;\mathrm{this}\;\mathrm{level}\right)}{\mathrm{total}\;\mathrm{number}\;\mathrm{of}\;\mathrm{investigated}\;\mathrm{leaves}\;\text{×}\;\mathrm{highest}\;\mathrm{value}}\times 100$$

$$\mathrm{Control}\;\mathrm{effect}=\frac{\mathrm{control}\;\mathrm{disease}\;\mathrm{index}\;-\;\mathrm{treatment}\;\mathrm{disease}\;\mathrm{index}}{\mathrm{control}\;\mathrm{disease}\;\mathrm{index}}\times 100\%$$

### 3.4. Degradation of Compounds ***17*** and ***23*** in Bananas [36]

Bananas of unblemished and uniform size, procured from the local market, were immersed in a solution containing 200 μg/mL of the test compound for a period of 30 min. The test compound was dissolved with DMSO and configured into 1000 μg/mL mother liquor, which was diluted to the required concentration with 0.5% Tween-80 water. The evaluation of each compound was conducted in triplicate. Following immersion, the peels and pulps of the bananas were collected at specified time intervals: 2 h, 4 h, 24 h, 72 h, and 120 h after exposure to the compound solution. A 10 g portion of the collected sample was homogenized using a tissue grinder. Subsequently, 50 mL of dichloromethane was added to the homogenate, followed by sonication and shaking for a duration of 30 min. The suspension was then filtered, and the filtrate was evaporated under reduced pressure. The residue was redissolved in chromatographic-grade methanol and diluted to a final volume of 10 mL. The solution filtered through a 0.22 μm organic phase filter membrane and was transferred into a sample vial for analysis.

Chromatographic conditions. The analysis was performed on a Cosmosil 5C18-MS-II column (5 μm, 4.6 × 250 mm) with the column temperature maintained at 25 °C. A sample volume of 10 μL was injected for analysis. The mobile phase comprised a mixture of 30% water and 70% methanol, and the flow rate was set to 1 mL/min.

Linear regression equation. Five distinct concentrations of standard solutions were prepared by diluting the original standard solution with methanol. The test compound injection concentration (x, μg/mL) was as the abscissa and the corresponding chromatographic peak area (y) as the ordinate for the linear regression calculation. The linear equation for compound **17** was y = 15.31x − 359.99 (R^2^, 0.997) (Figure S72), and the linear equation for compound **23** was y = 14.922x + 160.09 (R^2^, 0.9999) (Figure S74).

Data analysis. The dissipation of the test compound in bananas over time was evaluated using a first-order kinetic equation, and the dissipation dynamic equation and half-life were calculated using the following formula:

C_t_
*=* C_0_e^−kt^

t_1/2_ = ln2/k = 0.693/k

where C_t_ represents the residue concentration (mg/kg) at time t, C_0_ is the initial concentration (mg/kg), k is the dissipation coefficient, and t_1/2_ is the time required for test compound residue level to decrease to half of the initial residue concentration. Statistical analysis was performed using OriginPro 2024 SR1 10.1.0.178.

### 3.5. Molecular Docking Study

The crystal structure of the target protein, succinate dehydrogenase (SDH) (PDB code: 2FBW) [37], retrieved from the RCSB Protein Data Bank (https://www.rcsb.org, accessed on 15 February 2025), had its non-protein components removed using Discovery Studio Visualizer (version 24.1.0, Build 23298, developed by Dassault Systèmes, Vélizy-Villacoublay, France). Molecular docking experiments were conducted using AutoDockTools (version 1.5.7, The Scripps Research Institute, San Diego, CA, USA), adhering to the methodology outlined by Olson [38]. The docking procedures were carried out via the AutoDock algorithm. For global docking, a grid was used that encompassed the binding region between the B chain and the C and D chains of succinate dehydrogenase, with detailed information depicted in Figure S75 of the Supporting Information. The default settings were maintained for the majority of the docking parameters, with the exception of the following adjustment: the ‘Number of GA Runs’ was increased to 100. Visualization of the docking outcomes was achieved with PyMOL (version 3.1.3, provided by Schrödinger, New York, NY, USA), and the 2D diagrams illustrating ligand interactions were created using Discovery Studio Visualizer.

## 4. Conclusions

In this study, we employed a scaffold hopping approach to transform the α, β-unsaturated ketones of the antifungal lead compound dehydrozingerone into pyridine derivatives, resulting in the design and synthesis of a series of structurally novel trifluoromethylpyridine compounds. Our evaluation of their antifungal properties revealed that most compounds have good in vitro antifungal activity, especially in inhibiting *Rhizoctonia solani* and *Colletotrichum musae*. Compound **17** exhibited the most efficient and broad-spectrum in vitro antifungal activity, with EC_50_ values ranging from 2.88 to 9.09 µg/mL. These phenoxytrifluoromethylpyridine derivatives exhibit superior antifungal efficacy compared to the benzyloxytrifluoromethylpyridine analogs. The antifungal activity is notably enhanced when the trifluoromethyl and ether groups are positioned on the same side of the nitrogen atom in the pyridine ring. Furthermore, the introduction of chlorine substitutions on the phenyl ring contributes to increased activity. Compound **17** in particular demonstrates broad-spectrum antifungal activity and is a promising candidate for further development as a lead compound. Both compounds **17** and **23** exhibited moderate in vivo control effects against *C. musae*. A degradation study of compounds **17** and **23** in bananas was conducted, and it was found that their half-lives were 176.9 and 94.8 h, respectively, indicating the stability of their structures in the environment. Molecular docking studies revealed that compound **23** interacts with succinate dehydrogenase with a binding energy of −7.03 kcal/mol, forming hydrogen bonds with the 39th SER residue of chain C and the 173rd TRP residue of chain B. These strong interactions provide valuable insights for the structural optimization of compound **23**.

## Figures and Tables

**Table 1:** Inhibitory rate of target compounds on plant pathogen mycelium growth.

Con. ^a^	Compound	Inhibition Rate ± SD (%)					
		*R. s* ^b^	*P. o*	*C. m*	*F. g*	*B. c*	*C. s*
50	**1**	93.5 ± 0.7	79.1 ± 2.4	86.1 ± 0.7	76.8 ± 1.5	90.7 ± 1.8	55.7 ± 3.5
50	**2**	67.3 ± 2.4	28.6 ± 3.1	47.1 ± 4.1	10.7 ± 7.7	33.2 ± 3.5	23.8 ± 0.7
50	**3**	86.2 ± 1.1	33.6 ± 3.7	68.4 ± 7.9	58.4 ± 10.6	53.1 ± 14.2	42.9 ± 2.1
50	**4**	79.1 ± 0.0	59.7 ± 1.4	81.8 ± 0.6	39.1 ± 1.2	15.6 ± 2.1	46.3 ± 4.1
50	**5**	86.4 ± 0.6	48.1 ± 2.0	84.9 ± 2.8	44.7 ± 3.4	48.3 ± 6.7	51.4 ± 0.7
50	**6**	74.0 ± 1.0	25.7 ± 5.5	74.3 ± 2.8	28.1 ± 1.4	39.6 ± 1.4	32.4 ± 2.2
50	**7**	78.8 ± 0.7	30.3 ± 1.8	72.5 ± 7.0	53.7 ± 6.1	17.6 ± 4.5	37.3 ± 4.2
50	**8**	76.8 ± 1.5	21.1 ± 3.3	65.4 ± 3.6	35.5 ± 3.4	37.9 ± 10.2	26.4 ± 3.0
50	**9**	91.6 ± 0.0	73.8 ± 0.7	87.0 ± 0.0	67.9 ± 2.5	95.8 ± 1.2	66.7 ± 0.7
50	**10**	27.2 ± 1.7	8.4 ± 4.6	13.3 ± 2.3	26.1 ± 3.5	13.25 ± 1.0	6.21 ± 2.1
50	**11**	80.2 ± 1.7	27.5 ± 5.2	70.7 ± 4.5	37.8 ± 23.8	43.0 ± 1.5	29.4 ± 7.1
50	**12**	86.5 ± 3.7	84.2 ± 6.1	79.8 ± 6.9	78.6 ± 1.5	87.3 ± 3.5	52.8 ± 3.3
10	**13**	40.1 ± 1.7	25.3 ± 0.7	6.5 ± 2.4	6.3 ± 1.1	14.0 ± 4.0	−0.5 ± 0.7
10	**14**	59.0 ± 1.2	33.9 ± 3.5	10.2 ± 0.7	15.1 ± 3.4	5.8 ± 6.6	9.7 ± 1.4
10	**15**	15.0 ± 0.7	5.6 ± 2.6	13.4 ± 3.5	−2.2 ± 1.7	30.3 ± 0.7	4.2 ± 2.7
10	**16**	60.8 ± 1.3	57.4 ± 4.6	59.6 ± 6.2	31.0 ± 1.8	49.2 ± 6.8	16.2 ± 1.4
10	**17**	76.8 ± 1.1	60.3 ± 0.6	75.1 ± 0.7	63.0 ± 1.3	79.6 ± 1.4	48.5 ± 1.4
10	**18**	31.3 ± 1.1	0.0 ± 0.5	20.7 ± 1.5	23.0 ± 9.0	8.5 ± 1.9	12.5 ± 1.5
10	**19**	1.8 ± 3.4	11.5 ± 0.0	19.7 ± 2.5	7.1 ± 1.2	−9.7 ± 3.3	9.8 ± 5.9
10	**20**	8.0 ± 3.5	14.7 ± 0.7	7.9 ± 4.7	5.4 ± 3.5	−13.5 ± 2.6	9.4 ± 2.2
10	**21**	7.8 ± 4.7	2.7 ± 1.6	13.1 ± 5.0	4.8 ± 1.8	−18.9 ± 10.1	5.8 ± 1.4
10	**22**	37.1 ± 2.4	25.8 ± 2.1	40.3 ± 1.1	26.4 ± 2.2	14.5 ± 2.2	25.0 ± 3.4
10	**23**	70.5 ± 1.3	54.1 ± 2.7	81.1 ± 3.1	58.6 ± 3.1	36.8 ± 11.3	42.4 ± 2.5
50	pyrimethanil	84.0 ± 1.3	65.9 ± 1.1	75.2 ± 14.3	39.9 ± 2.8	100.0 ± 0.0	26.1 ± 1.6
10	azoxystrobin	58.4 ± 0.6	46.0 ± 1.3	54.2 ± 2.1	47.6 ± 3.4	- ^c^	39.0 ± 0.8

Note: ^a^: concentration. ^b^: *R. s*, *Rhizoctonia solani*; *P. o*, *Pyricularia oryzae*; *C. m*, *Colletotrichum musae*; *F. g*, *Fusarium graminearum*; *B. c*, *Botrytis cinerea*; and *C. s*, *Colletotrichum siamense*. ^c^: Not tested.

**Table 2:** EC_50_ ^a^ values of some target compounds against six pathogenic fungi (µg/mL).

Compound	*R s* ^b^	*P. o*	*C. m*	*F. g*	*B. c*	*C. s*
**1**	8.08	24.06	17.27	31.70	21.96	- ^c^
**16**	7.28	14.98	12.08	22.14	4.17	52.19
**17**	2.88	5.96	4.32	6.23	2.99	9.09
**23**	4.24	8.92	3.20	44.40	18.43	14.33
azoxystrobin	<0.01	12.21	5.09	16.54	-	31.20

Note: ^a^: The detailed toxicity equation is presented in Table S1. ^b^: *R. s*, *Rhizoctonia solani*; *P. o*, *Pyricularia oryzae*; *C. m*, *Colletotrichum musae*; *F. g*, *Fusarium graminearum*; *B. c*, *Botrytis cinerea*; and *C. s*, *Colletotrichum siamense*. ^c^: Not tested.

**Table 3:** In vivo protective effect of compounds against *Colletotrichum musae* (%).

Compound	Concentration (μg/mL)	Protective Effect	
		Disease Index	Control Efficacy (%)
**17**	200	46.67 ± 1.56 ^b^	46.24 ± 2.34 ^b^
**23**	200	42.22 ± 0.91 ^b^	48.45 ± 1.46 ^b^
azoxystrobin	200	24.44 ± 0.32 ^c^	70.27 ± 0.67 ^a^
CK	-	82.22 ± 0.54 ^a^	-

Note: Lowercase letters represent one-way ANOVA results between different groups at the same time point (Duncan analysis *p* ≤ 0.05). a, b and c indicate significant differences between different groups, and a, b, and c are labeled in descending order of inhibition rate.

## Data Availability

Data have been stored in the library repository of Hainan University, Haikou, China.

## Supplementary Materials

The following supporting information can be downloaded at: https://www.mdpi.com/article/10.3390/ijms26115345/s1.

## References

[1] Stukenbrock E., Gurr S. (2023). Address the Growing Urgency of Fungal Disease in Crops. Nature.

[2] Song R., Zhang Y., Lu P., Wu J., Li Q.X., Song B. (2024). Status and Perspective on Green Pesticide Utilizations and Food Security. Annu. Rev. Food Sci. Technol..

[3] Fones H.N., Bebber D.P., Chaloner T.M., Kay W.T., Steinberg G., Gurr S.J. (2020). Threats to Global Food Security from Emerging Fungal and Oomycete Crop Pathogens. Nat. Food.

[4] Fisher M.C., Hawkins N.J., Sanglard D., Gurr S.J. (2018). Worldwide Emergence of Resistance to Antifungal Drugs Challenges Human Health and Food Security. Science.

[5] Case N.T., Gurr S.J., Fisher M.C., Blehert D.S., Boone C., Casadevall A., Chowdhary A., Cuomo C.A., Currie C.R., Denning D.W. (2025). Fungal Impacts on Earth’s Ecosystems. Nature.

[6] Sparks T.C., Sparks J.M., Duke S.O. (2023). Natural Product-Based Crop Protection Compounds─Origins and Future Prospects. J. Agric. Food Chem..

[7] Späth G., Loiseleur O. (2024). Chemical Case Studies from Natural Products of Recent Interest in the Crop Protection Industry. Nat. Prod. Rep..

[8] De Bernardi M., Vidari G., Vita-Finzi P. (1976). Dehydrozingerone from *Aframomum Giganteum*. Phytochemistry.

[9] Hampannavar G.A., Karpoormath R., Palkar M.B., Shaikh M.S. (2016). An Appraisal on Recent Medicinal Perspective of Curcumin Degradant: Dehydrozingerone (DZG). Bioorganic Med. Chem..

[10] Kumar V., Bala R., Dhawan S., Singh P., Karpoormath R. (2022). The Multi-Biological Targeted Role of Dehydrozingerone and Its Analogues. ChemistrySelect.

[11] Agarwal M., Walia S., Dhingra S., Khambay B.P.S. (2001). Insect Growth Inhibition, Antifeedant and Antifungal Activity of Compounds Isolated/Derived from *Zingiber officinale* Roscoe (Ginger) Rhizomes. Pest Manag. Sci..

[12] Hanssen B.L., Park S.J., Royer J.E., Jamie J.F., Taylor P.W., Jamie I.M. (2019). Systematic Modification of Zingerone Reveals Structural Requirements for Attraction of Jarvis’s Fruit Fly. Sci. Rep..

[13] Jung K.-S., Lee Y., Ganbat D., Park S.J., Lee S.-E. (2025). Antifungal and Antimycotoxigenic Activities of a Synthetic Zingerone-Derivative 4-(4-Hydroxy-3-Nitrophenyl)-2-Butanone against *Aspergillus flavus* and *Fusarium graminearum*. Appl. Food Res..

[14] Song X., Zhu X., Li T., Liang C., Zhang M., Shao Y., Tao J., Sun R. (2019). Dehydrozingerone Inspired Discovery of Potential Broad-Spectrum Fungicidal Agents as Ergosterol Biosynthesis Inhibitors. J. Agric. Food Chem..

[15] Zhang M., Dou M., Xia Y., Hu Z., Zhang B., Bai Y., Xie J., Liu Q., Xie C., Lu D. (2021). Photostable 1-Trifluoromethyl Cinnamyl Alcohol Derivatives Designed as Potential Fungicides and Bactericides. J. Agric. Food Chem..

[16] Burriss A., Edmunds A.J.F., Emery D., Hall R.G., Jacob O., Schaetzer J. (2018). The Importance of Trifluoromethyl Pyridines in Crop Protection. Pest Manag. Sci..

[17] Zheng Z., Dai A., Jin Z., Chi Y.R., Wu J. (2022). Trifluoromethylpyridine: An Important Active Fragment for the Discovery of New Pesticides. J. Agric. Food Chem..

[18] Tsukamoto M., Nakamura T., Kimura H., Nakayama H. (2021). Synthesis and Application of Trifluoromethylpyridines as a Key Structural Motif in Active Agrochemical and Pharmaceutical Ingredients. J. Pestic. Sci..

[19] Luo B., Ning Y. (2022). Comprehensive Overview of Carboxamide Derivatives as Succinate Dehydrogenase Inhibitors. J. Agric. Food Chem..

[20] Bao A.-L., Xie X.-S., Wang D.-Y., Deng Z.-Q., Chen Y., Liu D., Li W.-Y., Tang X.-R., Cheng W., Yan Y.-K. (2025). Design, Synthesis and Antifungal Activity of Novel Pyrazole-Amide-Isothiazole Derivatives as Succinate Dehydrogenase Inhibitors. Food Chem..

[21] Walter H. (2012). Pyrazole Carboxamide Fungicides Inhibiting Succinate Dehydrogenase. Bioactive Heterocyclic Compound Classes.

[22] Huang Y.H., Wei G., Wang W.J., Liu Z., Yin M.X., Guo W.M., Zhu X.L., Yang G.F. (2023). Structure-Based Discovery of New Succinate Dehydrogenase Inhibitors Via Scaffold Hopping Strategy. J. Agric. Food Chem..

[23] Su Y., Zhang T., An X., Ma H., Wang M. (2024). Design, Synthesis, Antifungal Activity and Molecular Docking of Novel Pyrazole-4-Carboxamides Containing Tertiary Alcohol and Difluoromethyl Moiety as Potential Succinate Dehydrogenase Inhibitors. Pest Manag. Sci..

[24] Sun N.-B., Min L.-J., Sun X.-P., Zhai Z.-W., Bajsa-Hirschel J., Wei Z.-C., Hua X.-W., Cantrell C.L., Xu H., Duke S.O. (2024). Novel Pyrazole Acyl(thio)urea Derivatives Containing a Biphenyl Scaffold as Potential Succinate Dehydrogenase Inhibitors: Design, Synthesis, Fungicidal Activity and SAR. J. Agric. Food Chem..

[25] Wang X., Wang A., Qiu L., Chen M., Lu A., Li G., Yang C., Xue W. (2020). Expedient Discovery for Novel Antifungal Leads Targeting Succinate Dehydrogenase: Pyrazole-4-Formylhydrazide Derivatives Bearing a Diphenyl Ether Fragment. J. Agric. Food Chem..

[26] Sauter H., Steglich W., Anke T. (1999). Strobilurins: Evolution of a New Class of Active Substances. Angew. Chem. Int. Ed..

[27] Lamberth C. (2023). Ring Closure and Ring Opening as Useful Scaffold Hopping Tools in Agrochemistry. J. Agric. Food Chem..

[28] Maienfisch P., Lamberth C. (2023). Introduction to Recent Highlights in Bioisosteric Replacements and Scaffold Hopping in Crop Protection Research. J. Agric. Food Chem..

[29] Kwan E.E., Zeng Y., Besser H.A., Jacobsen E.N. (2018). Concerted Nucleophilic Aromatic Substitutions. Nat. Chem..

[30] Frederique B., Dragos H. (2004). Molecular Similarity and Property Similarity. Curr. Top. Med. Chem..

[31] López-Pérez K., Avellaneda-Tamayo J.F., Chen L., López-López E., Juárez-Mercado K.E., Medina-Franco J.L., Miranda-Quintana R.A. (2024). Molecular Similarity: Theory, Applications, and Perspectives. Artif. Intell. Chem..

[32] Gijsen H.J., Berthelot D., Zaja M., Brône B., Geuens I., Mercken M. (2010). Analogues of Morphanthridine and the Tear Gas Dibenz[b,f][1,4]Oxazepine (CR) as Extremely Potent Activators of the Human Transient Receptor Potential Ankyrin 1 (TRPA1) Channel. J. Med. Chem..

[33] Hu Z., Yang B., Zheng S., Zhao K., Wang K., Sun R. (2024). Design, Synthesis, and Nematocidal Evaluation of Waltherione a Derivatives: Leveraging a Structural Simplification Strategy. Int. J. Mol. Sci..

[34] Li J.-L., Liu X.-Y., Xie J.-T., Di Y.-L., Zhu F.-X. (2015). A Comparison of Different Estimation Methods for Fungicide EC_50_ and EC_95_ Values. J. Phytopathol..

[35] Yu X., Zhu X., Zhou Y., Li Q., Hu Z., Li T., Tao J., Dou M., Zhang M., Shao Y. (2019). Discovery of N-Aryl-Pyridine-4-Ones as Novel Potential Agrochemical Fungicides and Bactericides. J. Agric. Food Chem..

[36] Cheng D., Lin S., Liu G., Zhou Y., Zhang Z. (2021). Residue and Distribution of Fosthiazate in Cucumber (*Cucumis sativus* L.) and Dietary Intake Risk Assessment for Various Populations. Int. J. Environ. Anal. Chem..

[37] Huang L.S., Sun G., Cobessi D., Wang A.C., Shen J.T., Tung E.Y., Anderson V.E., Berry E.A. (2006). 3-Nitropropionic Acid Is a Suicide Inhibitor of Mitochondrial Respiration That, upon Oxidation by Complex II, Forms a Covalent Adduct with a Catalytic Base Arginine in the Active Site of the Enzyme. J. Biol. Chem..

[38] Forli S., Huey R., Pique M.E., Sanner M.F., Goodsell D.S., Olson A.J. (2016). Computational Protein–Ligand Docking and Virtual Drug Screening with the Autodock Suite. Nat. Protoc..

