# Supplementary material for: Scaffold Hopping from Dehydrozingerone: Design, Synthesis, and Antifungal Activity of Phenoxyltrifluoromethylpyridines
Source: Int J Mol Sci. 2025 Jun 2;26(11):5345. doi: 10.3390/ijms26115345 (PMC12155161; doi:10.3390/ijms26115345)
Supplement: Supplementary file 1 [file ijms-26-05345-s001.zip › ijms-3636165-supplementary.pdf]

Scaffold Hopping from Dehydrozingerone: Design, Synthesis, and Antifungal Activity of Phenoxyltrifluoromethylpyridines

Xiaohui Nan†, Kaifeng Wang†, Xinru Sun, Zhan Hu* and Ranfeng Sun *
Key Laboratory of Green Prevention and Control of Tropical Plant Diseases and Pests, Ministry of Education, School of Tropical Agriculture and Forestry, Hainan University, Danzhou 571737, China
† These authors contributed equally to this work.
* Correspondence: huzhan@hainanu.edu.cn (Z.H.); srf18@hainanu.edu.cn (R.S.); Tel.: +86-898-66256373 (R.S.)

(Supporting Information)

Table S1 EC_{50} Value of Compounds against Plant Pathogens

Compound	Phytopathogen	EC_{50} (μg/mL)	Toxic Regression Equation	R^2
**1**	*R. s* [a]	8.082	y= -2.807+3.094x	0.987
	P. o	24.062	y= -5.805+4.202x	0.998
	C.m	17.269	y= -8.247+6.665x	0.969
	F.g	31.699	y= -6.856+4.567x	0.996
	B.c	21.963	y= -2.910+2.169x	0.998
**16**	*R.s*	7.277	y= -2.203+2.556x	0.983
	P. o	14.984	y= -4.040+3.437x	0.943
	C. m	12.084	y= -3.247+3.000x	0.978
	F. g	22.136	y= -3.331+2.467x	0.978
	B. c	4.174	y= -2.184+3.519x	0.968
	C. s	52.189	y= -4.892+2.848x	0.901
**17**	*R.s*	2.876	y= -1.002+2.185x	0.992
	P.o	5.955	y= -1.604+2.070x	0.980
	C.m	4.323	y= -1.830+2.878x	0.993
	F.g	6.229	y= -1.617+2.036x	0.993
	B.c	2.994	y= -1.149+2.412x	0.975
	C.s	9.093	y= -2.052+2.140x	0.992
**23**	*R. s*	4.242	y= -1.433+2.284x	0.986
	P. o	8.918	y= -1.923+2.023x	0.974
	C. m	3.200	y= -1.203+2.380x	0.937
	F. g	44.394	y= -1.802+1.094x	0.844
	B. c	18.434	y= -3.994+3.156x	0.989
	C. s	14.332	y= -1.828+1.581x	0.975
azoxystrobin	*R. s*	<0.01	-	-
	P. o	12.211	y= -0.761+0.700x	0.902
	C. m	5.093	y= -0.520+0.726x	0.990
	F. g	16.541	y= -0.402+0.330x	0.981
	C. s	31.205	y= -1.644+1.100x	0.989

Note: [a] *R. s, Rhizoctonia solani*; *P. o, Pyricularia oryae*; *C. m*, *Colletotrichum musae*; *F. g*, *Fusarium graminearum*; *B. c Botrytis cinerea*; and *C. s*, *Colletotrichum siamense.*

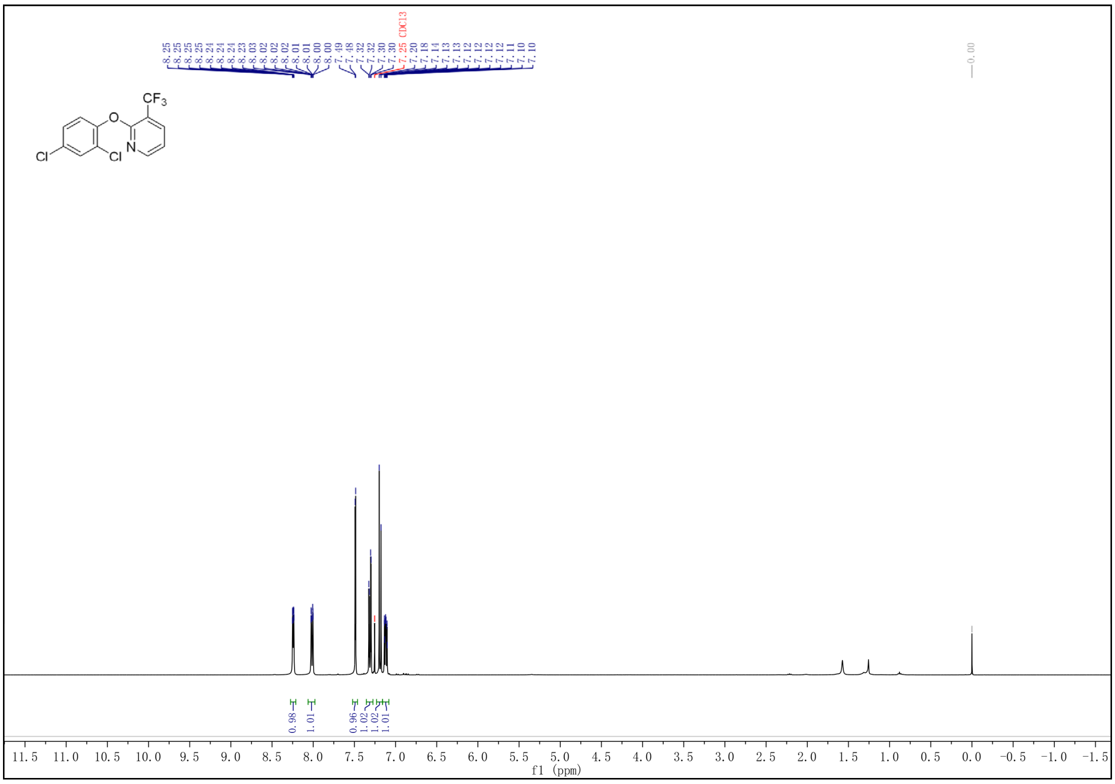

Fig. S1 ^{1}H NMR spectrum of compound **1**

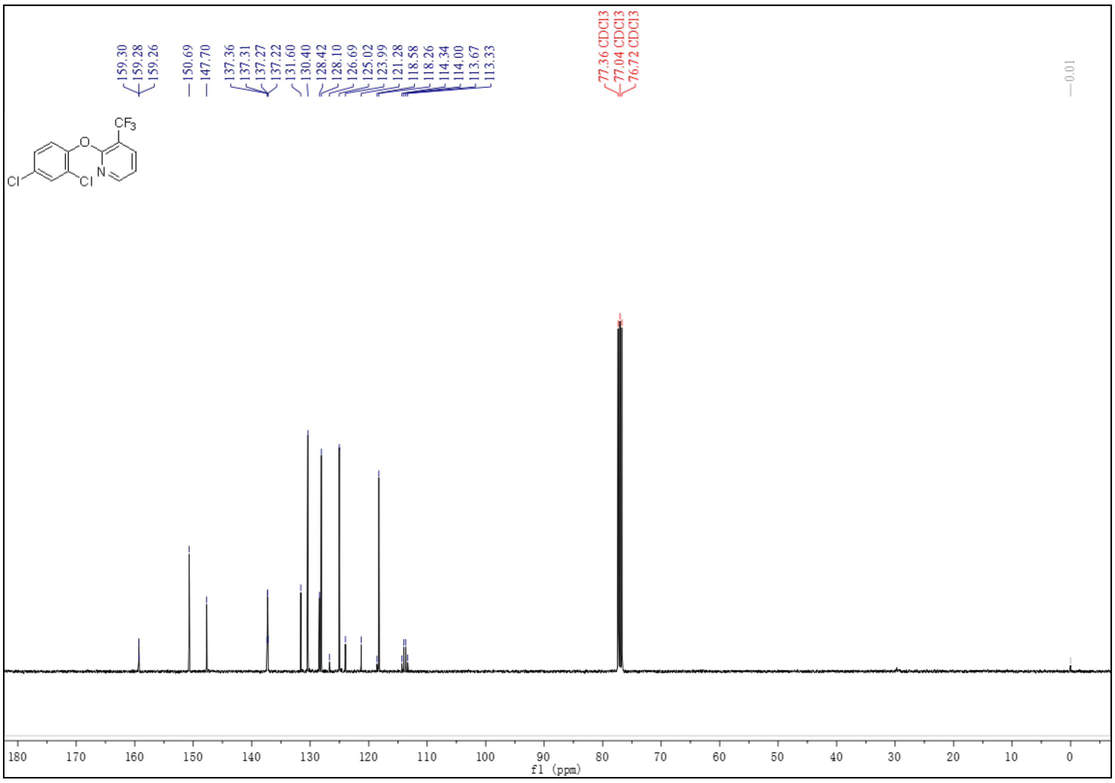

Fig. S2 ^{13}C NMR spectrum of compound **1**

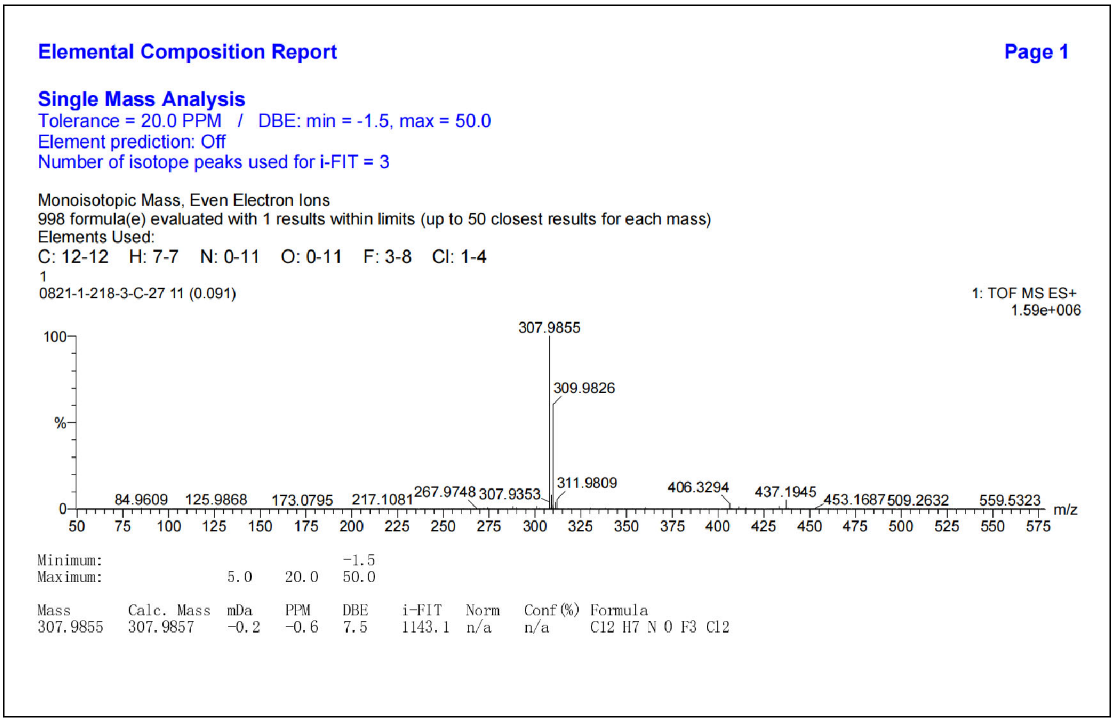

Minimum:				-1.5				
Maximum:		5.0	20.0	50.0				
Mass	Calc. Mass	mDa	PPM	DBE	i-FIT	Norm	Conf(%)	Formula
307.9855	307.9857	-0.2	-0.6	7.5	1143.1	n/a	n/a	C12 H7 N O F3 Cl2

Fig. S3 HRMS spectrum of compound **1**

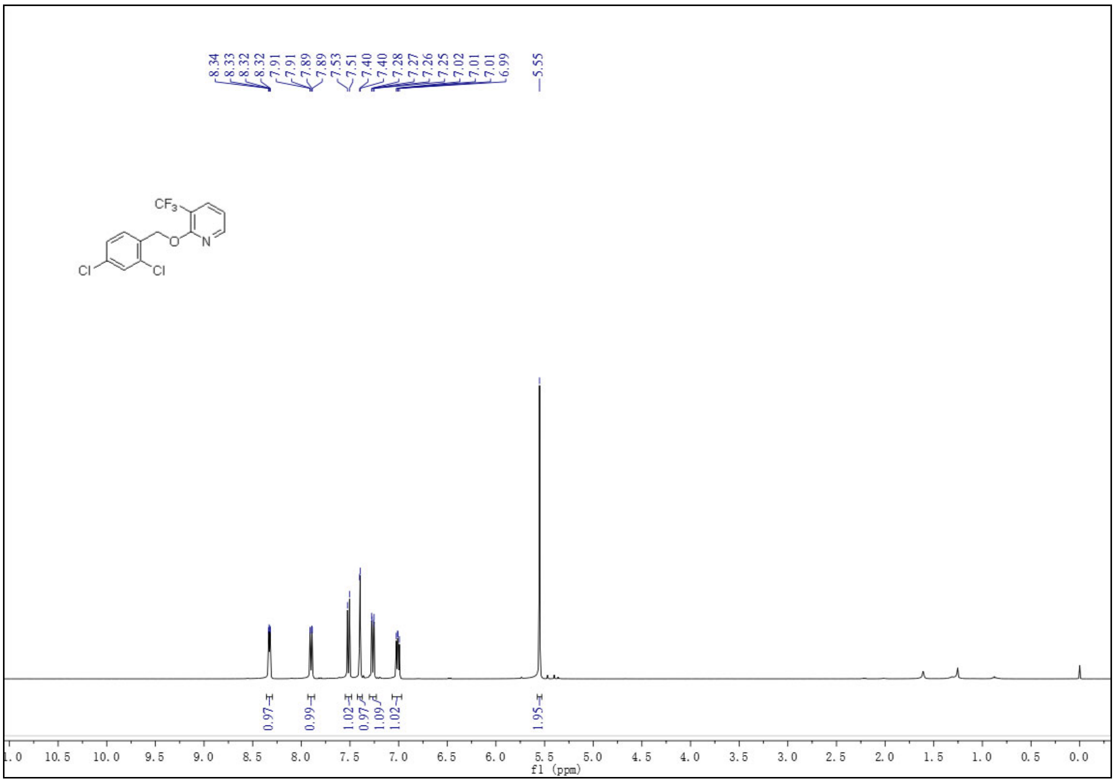

Fig. S4 ^{1}H NMR spectrum of compound **2**

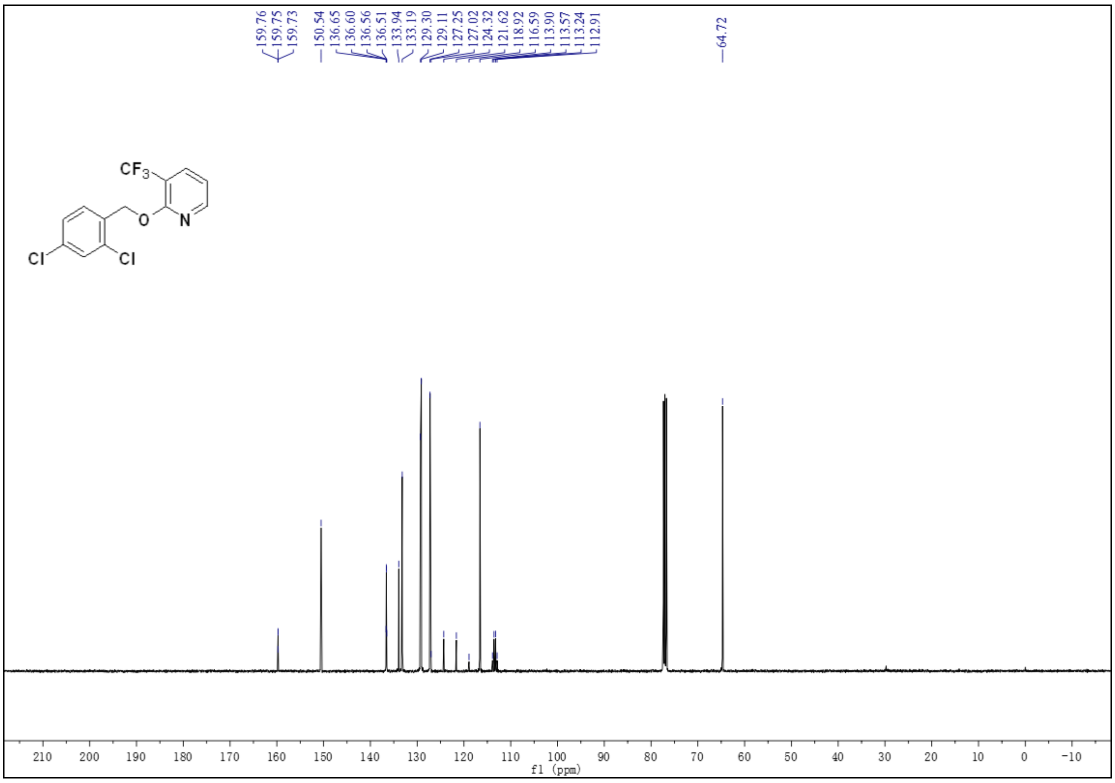

Fig. S5 ^{13}C NMR spectrum of compound **2**

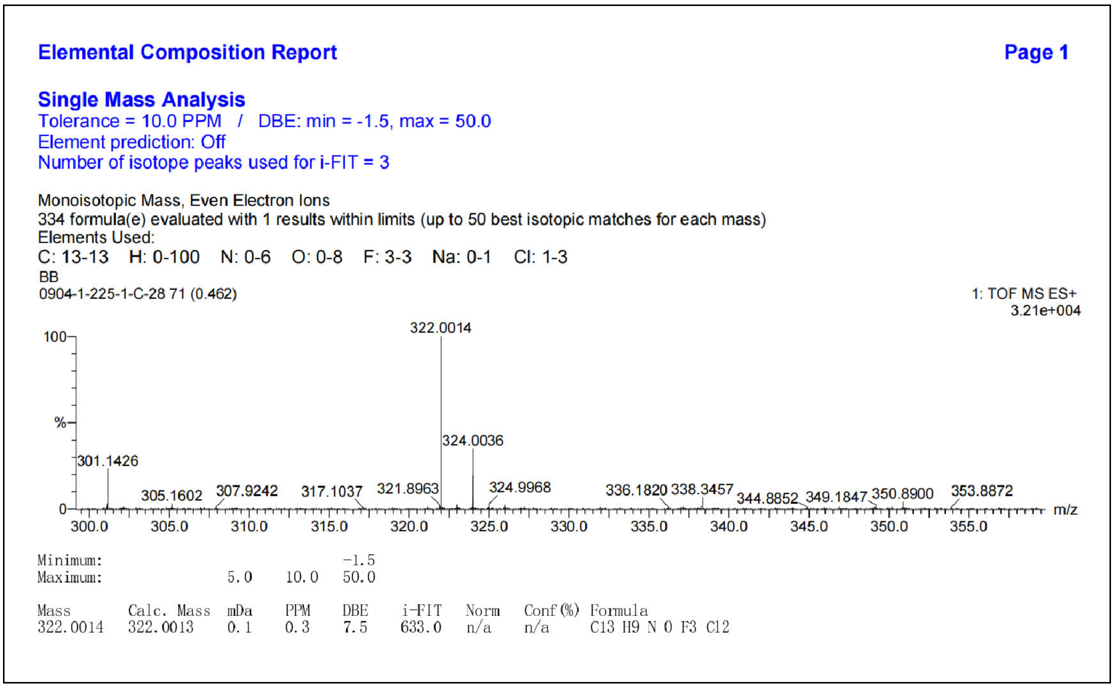

Fig. S6 HRMS spectrum of compound **2**

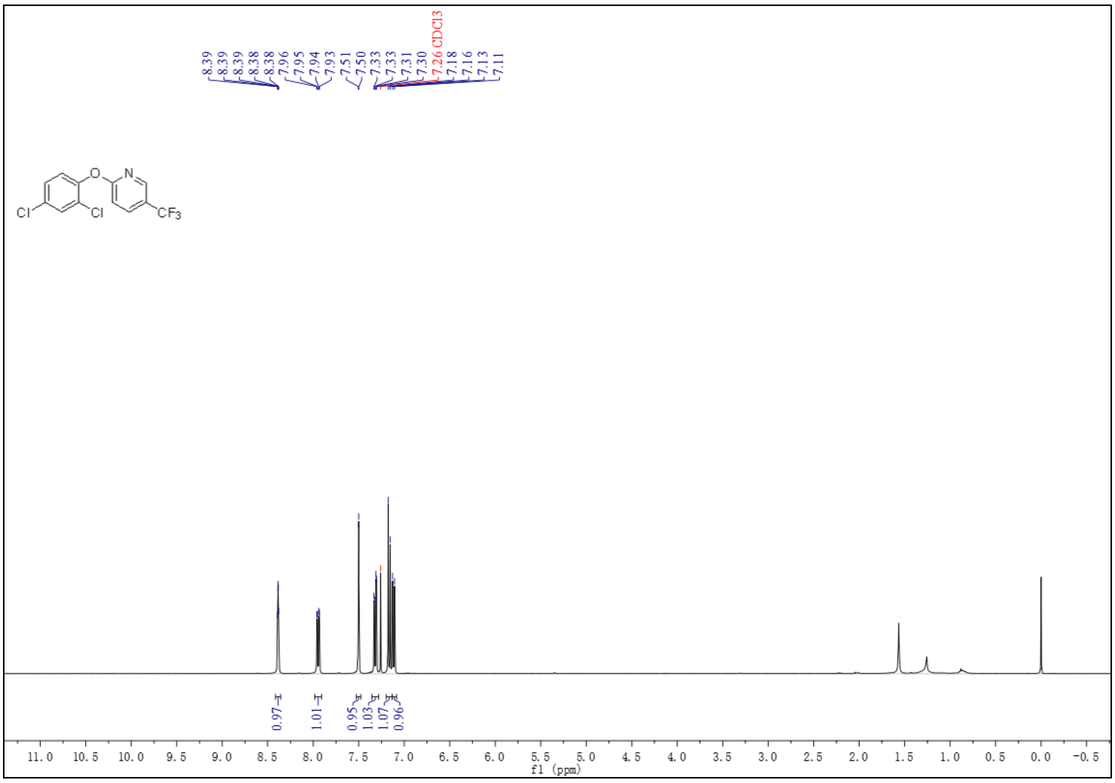

Fig. S7 ^{1}H NMR spectrum of compound **3**

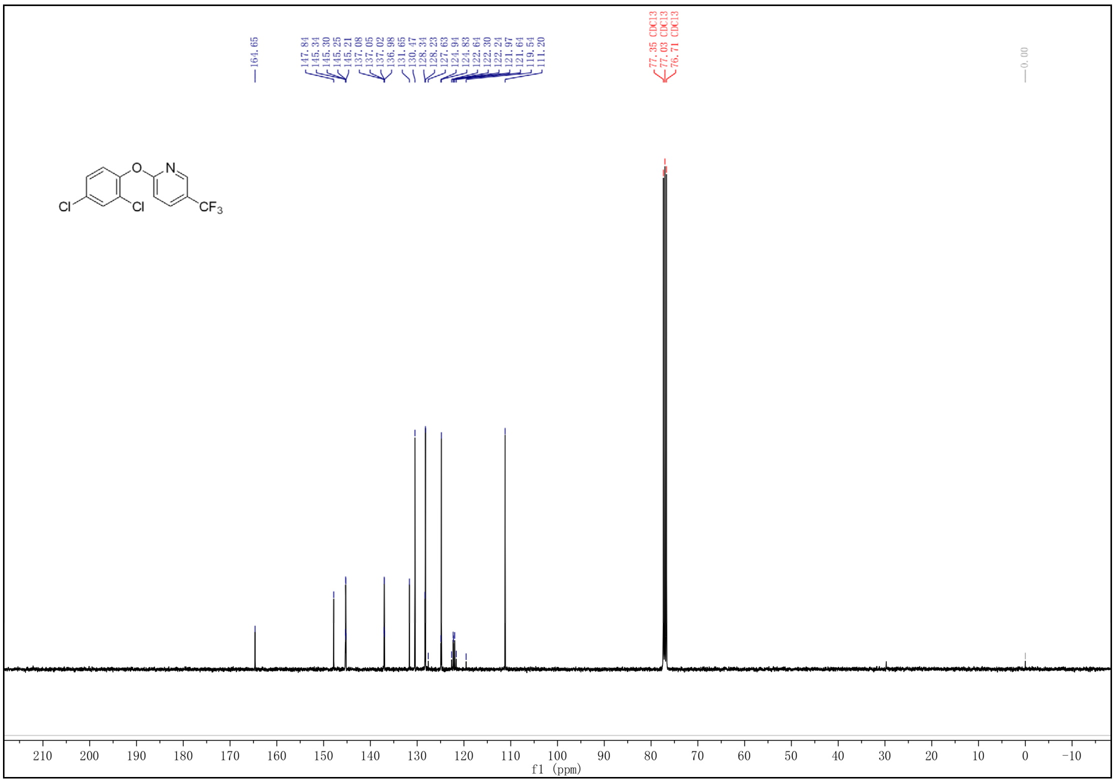

Fig. S8 ^{13}C NMR spectrum of compound **3**

Elemental Composition Report **Page 1**

Single Mass Analysis
Tolerance = 10.0 PPM / DBE: min = -1.5, max = 50.0
Element prediction: Off
Number of isotope peaks used for i-FIT = 3

Monoisotopic Mass, Even Electron Ions
293 formula(e) evaluated with 1 results within limits (up to 50 best isotopic matches for each mass)
Elements Used:
C: 12-12 H: 0-100 N: 0-4 O: 0-8 F: 3-4 Na: 0-1 Cl: 2-3
BB
0904-1-225-1-C-40 65 (0.430) 1: TOF MS ES+ 2.83e+006

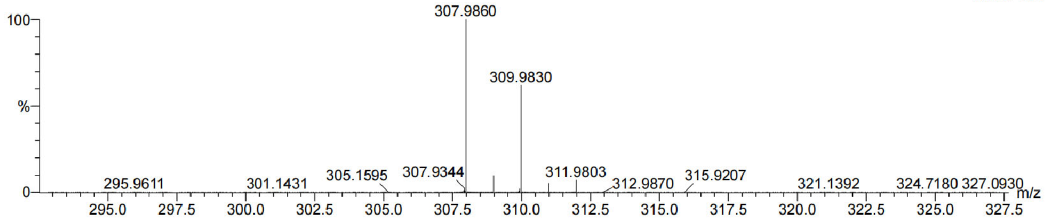

Minimum:				-1.5				
Maximum:		5.0	10.0	50.0				
Mass	Calc. Mass	mDa	PPM	DBE	i-FIT	Norm	Conf(%)	Formula
307.9860	307.9857	0.3	1.0	7.5	1331.3	n/a	n/a	C12 H7 N O F3 Cl2

Fig. S9 HRMS spectrum of compound **3**

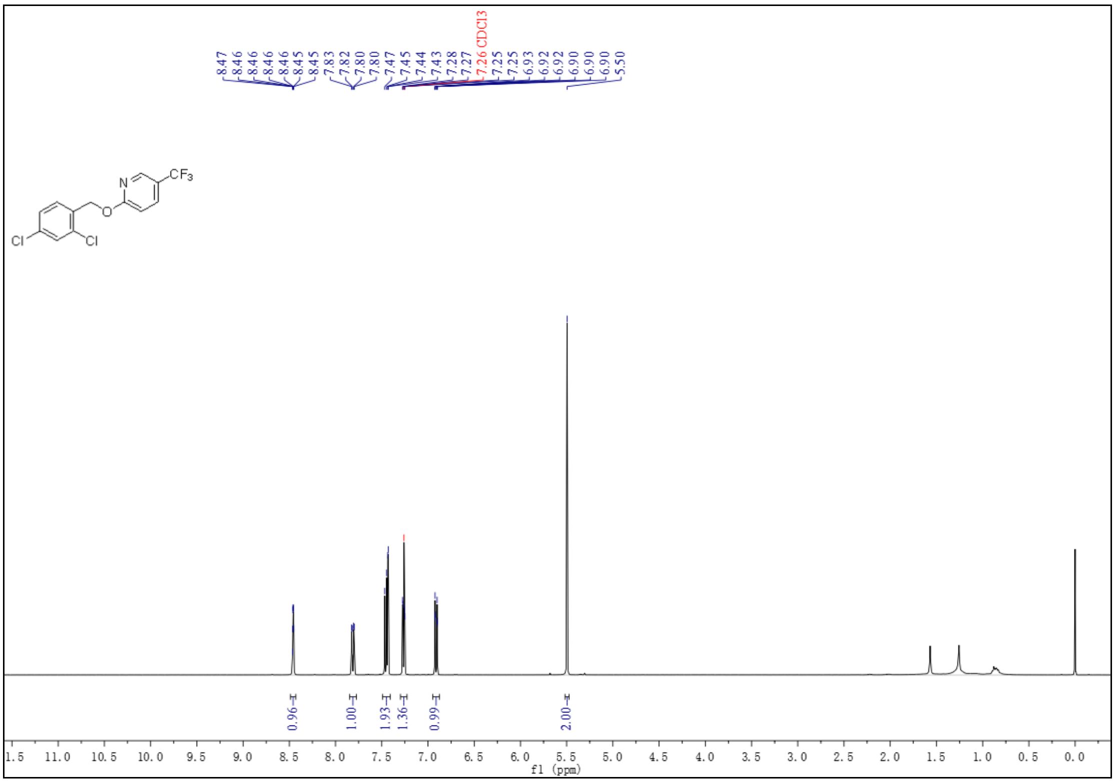

Fig. S10 ^{1}H NMR spectrum of compound **4**

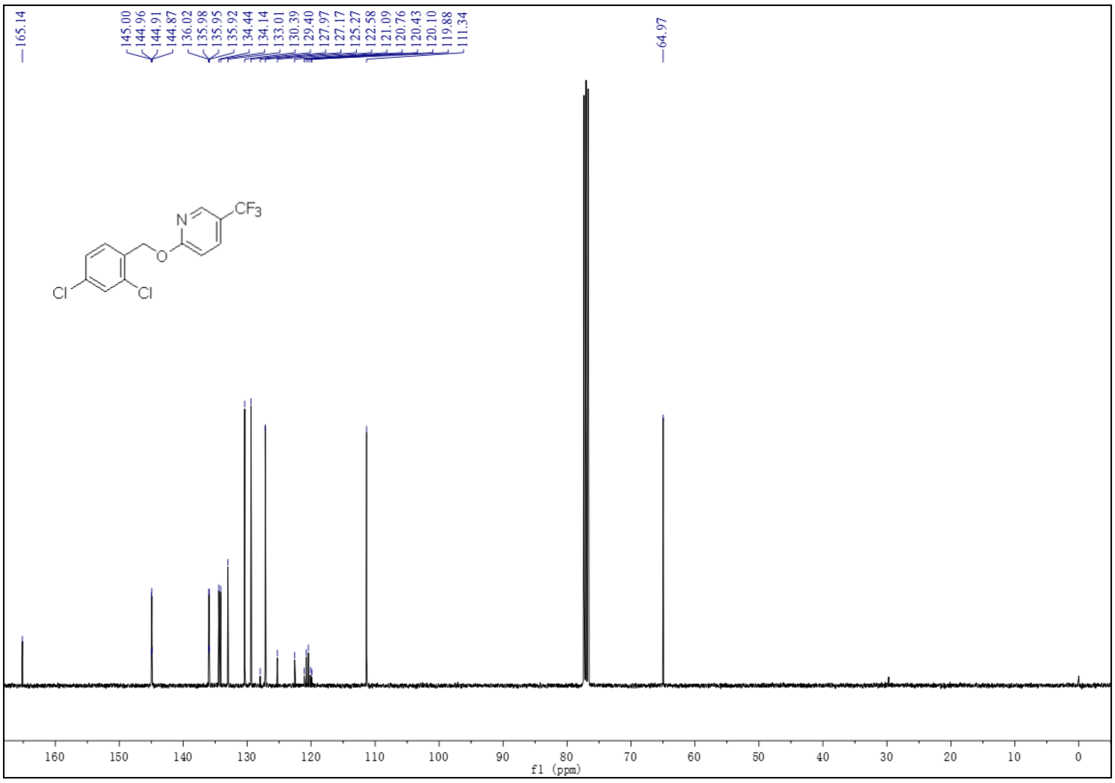

Fig. S11 ^{13}C NMR spectrum of compound **4**

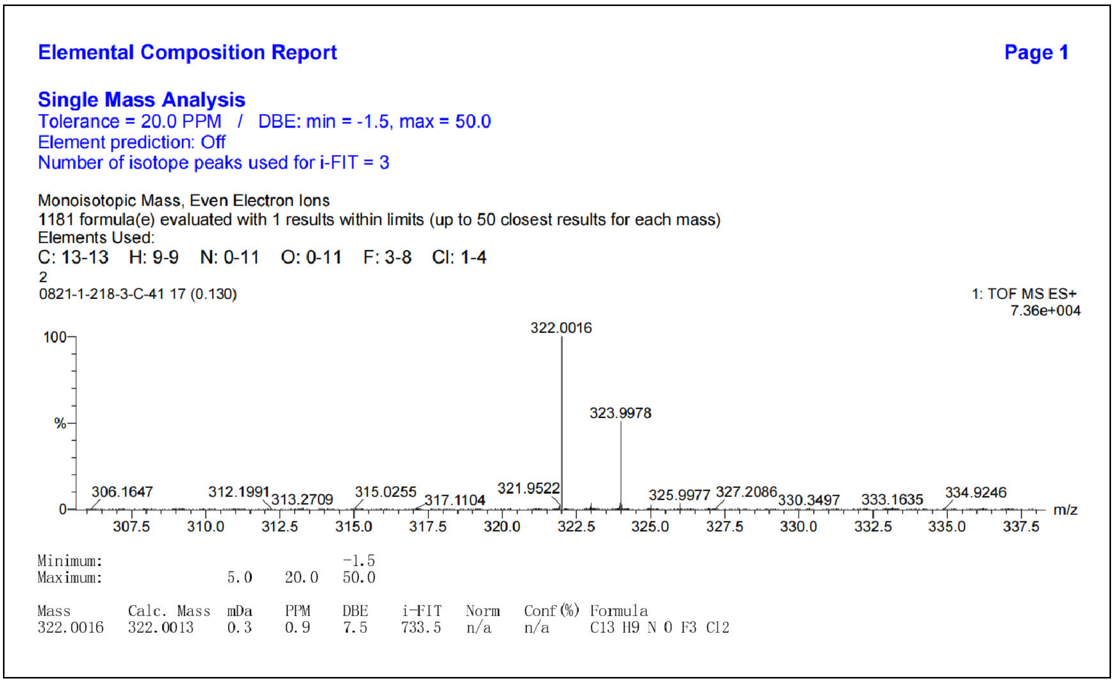

Fig. S12 HRMS spectrum of compound **4**

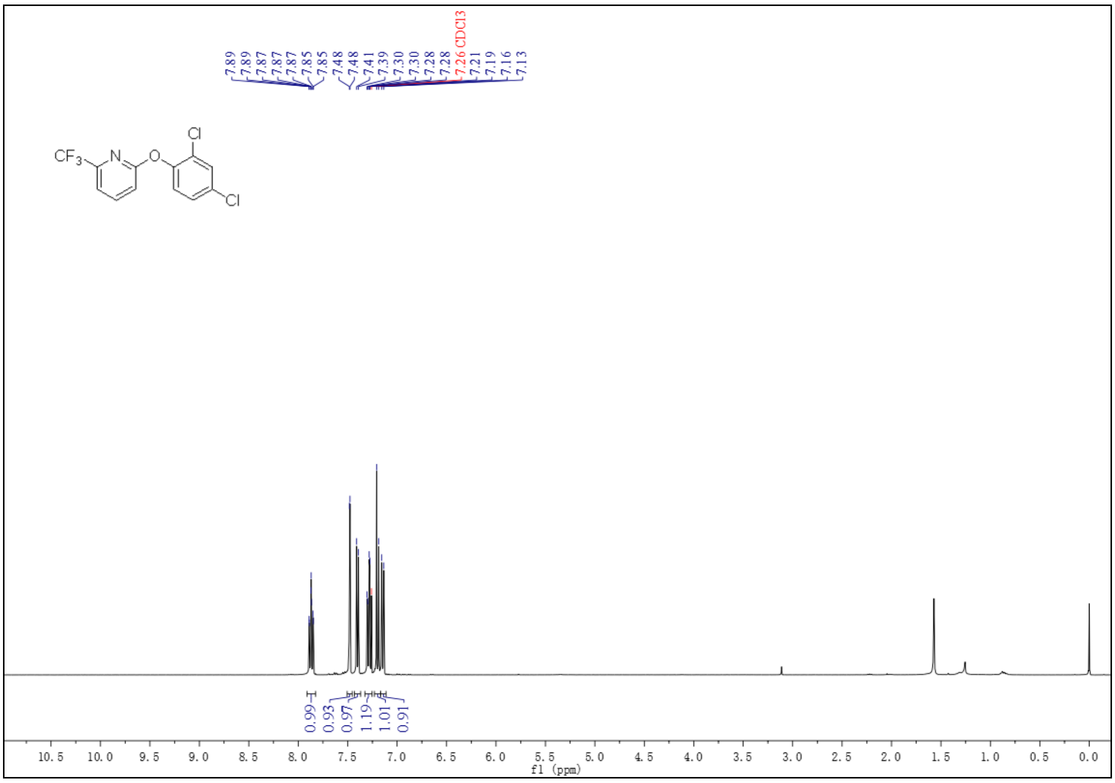

Fig. S13 ^{1}H NMR spectrum of compound **5**

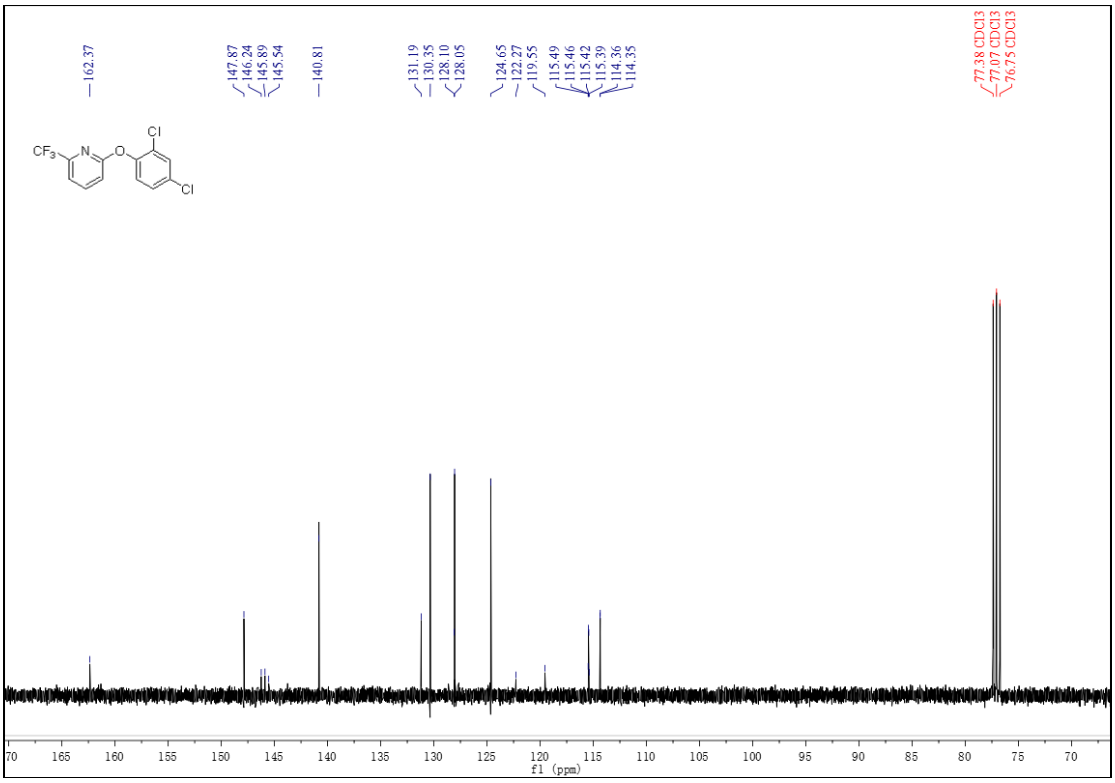

Fig. S14 ^{13}C NMR spectrum of compound **5**

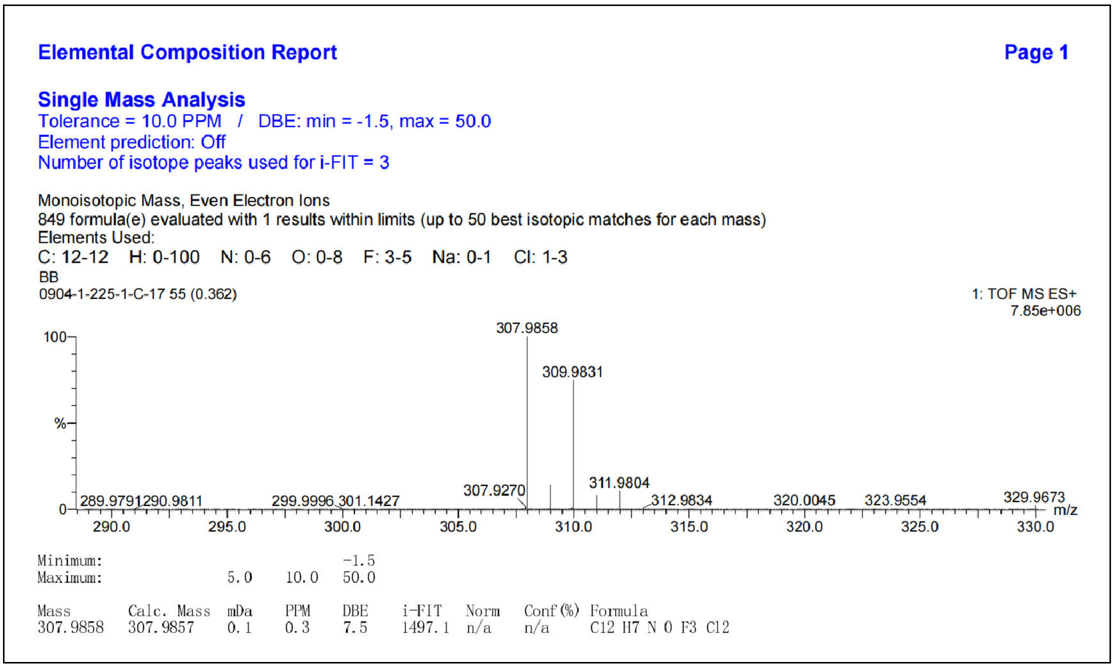

Fig. S15 HRMS spectrum of compound **5**

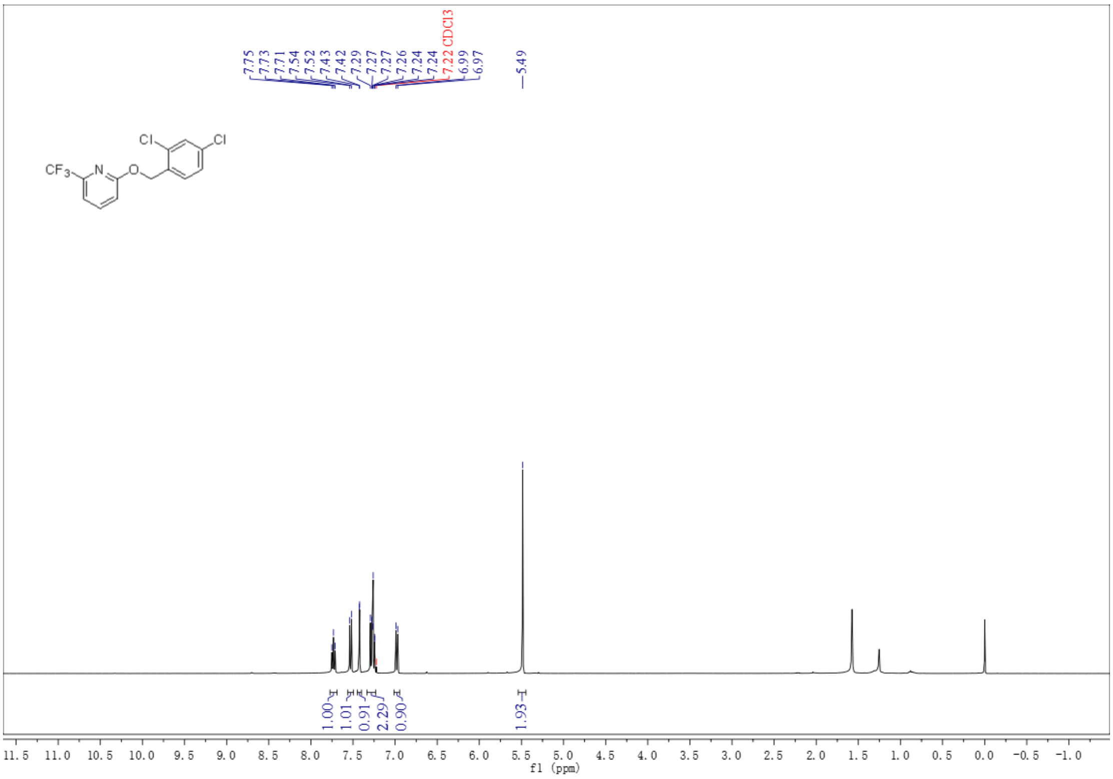

Fig. S16 ^{1}H NMR spectrum of compound **6**

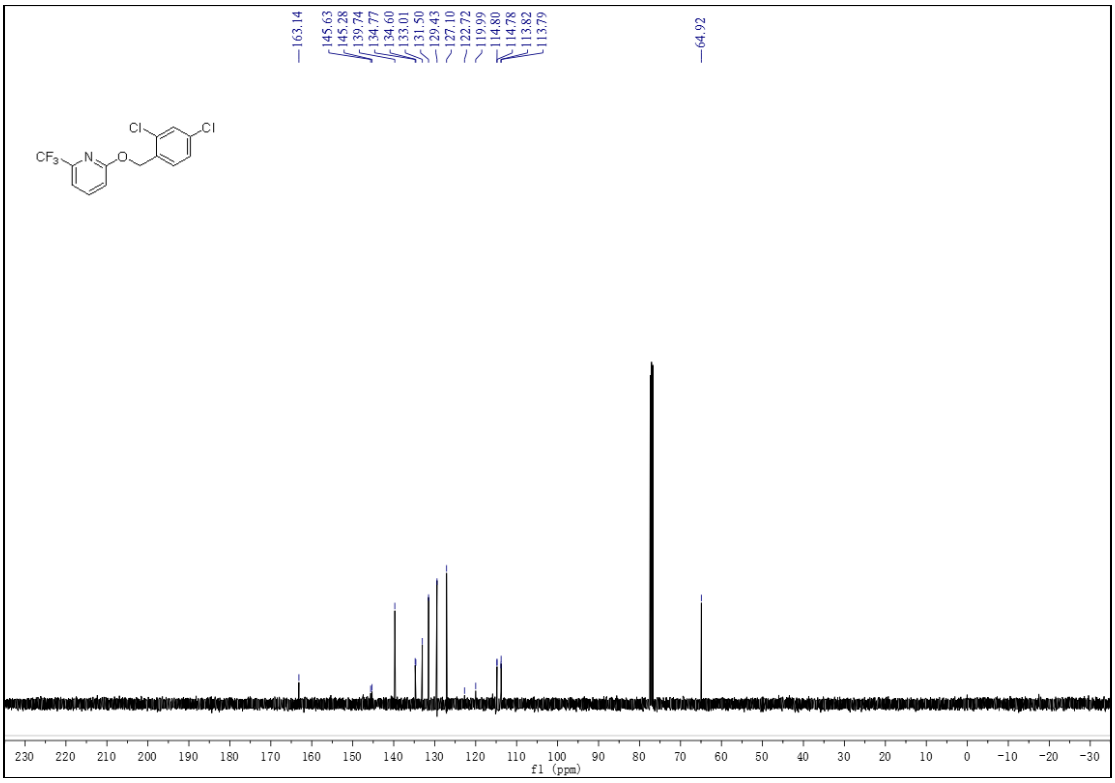

Fig. S17 ^{13}C NMR spectrum of compound **6**

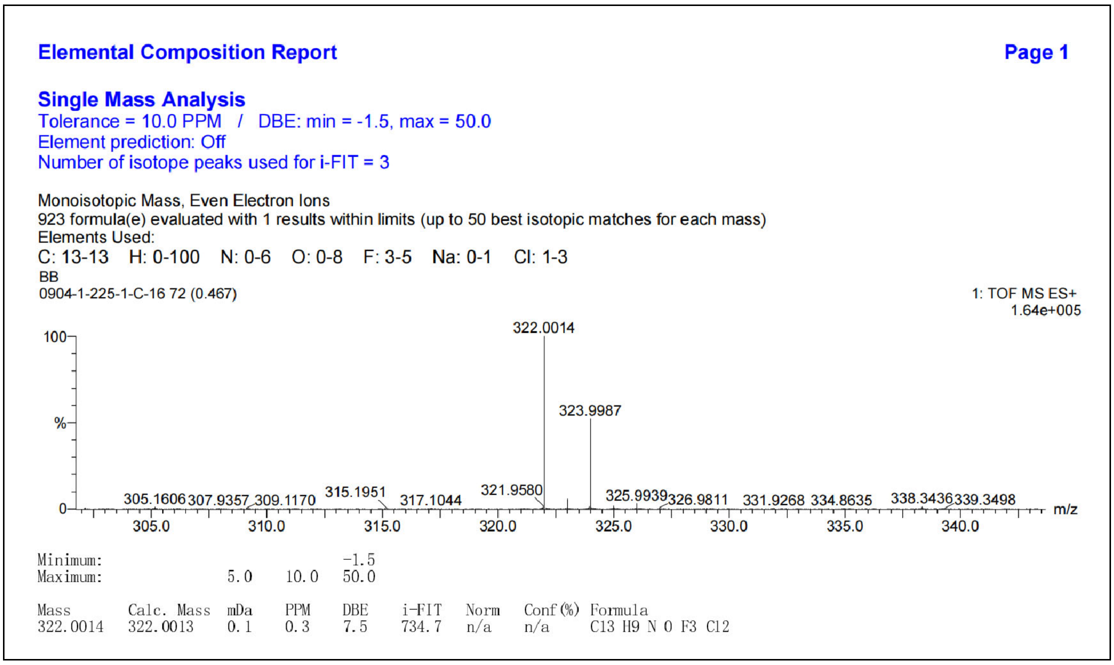

Fig. S18 HRMS spectrum of compound **6**

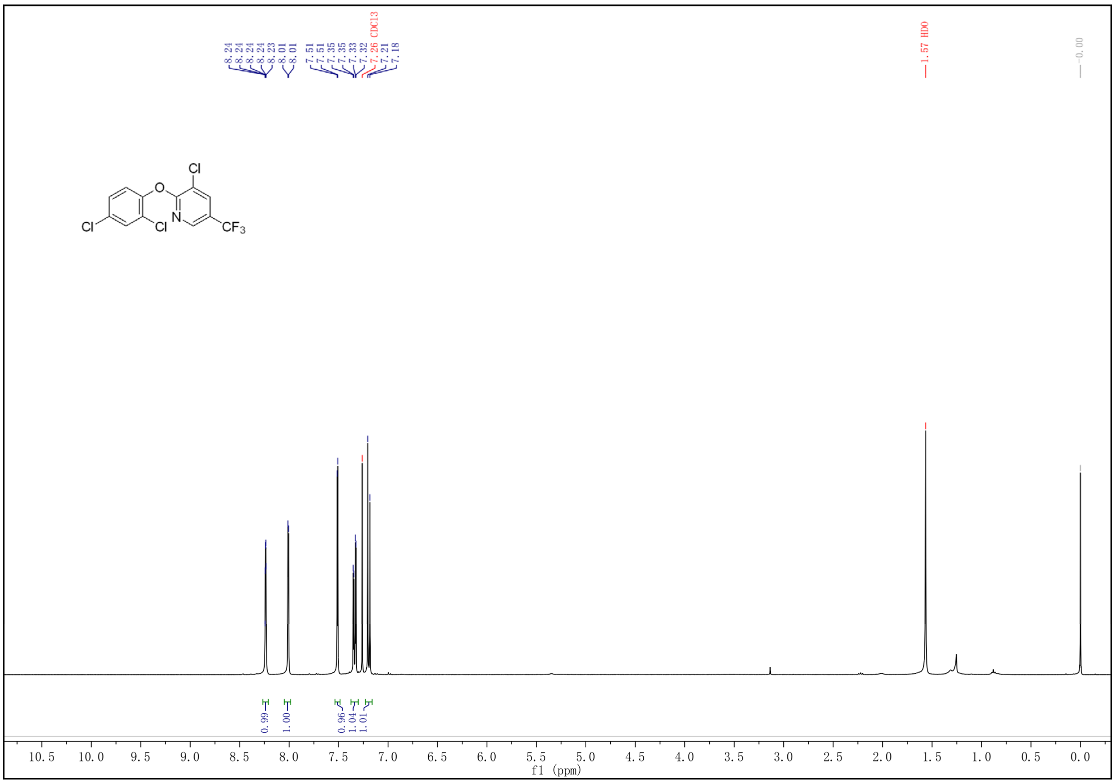

Fig. S19 ^{1}H NMR spectrum of compound **7**

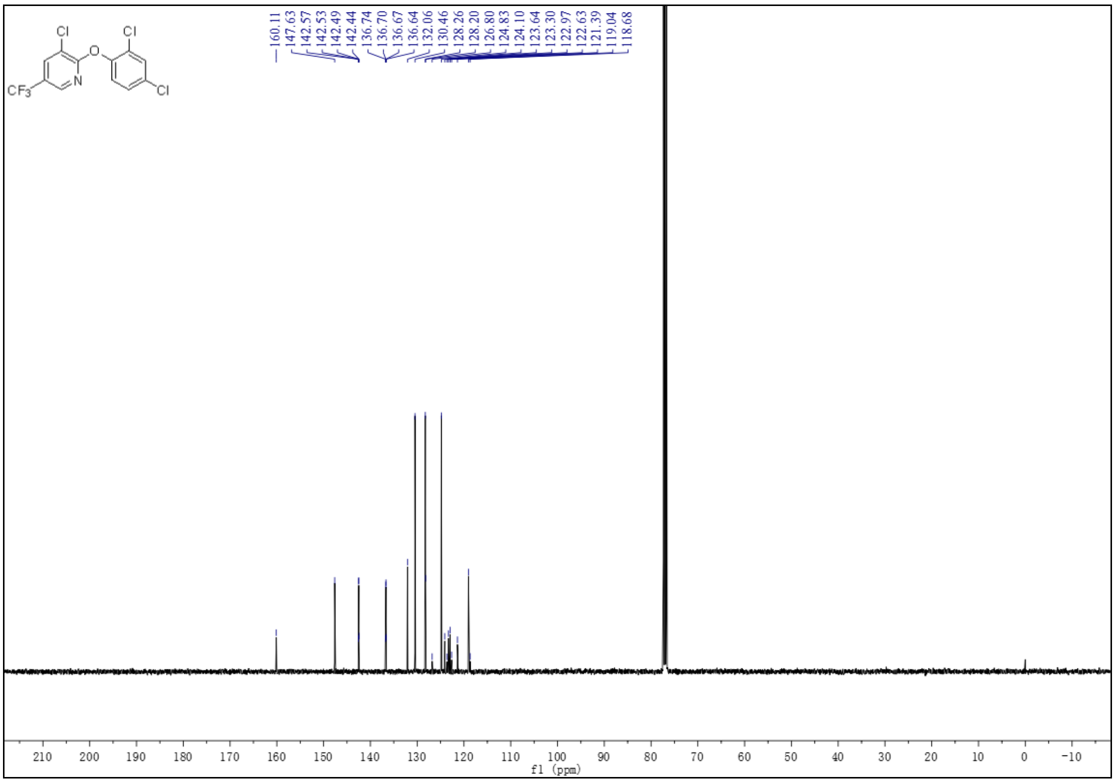

Fig. S20 ^{13}C NMR spectrum of compound **7**

Elemental Composition Report **Page 1**

Single Mass Analysis
Tolerance = 10.0 PPM / DBE: min = -1.5, max = 50.0
Element prediction: Off
Number of isotope peaks used for i-FIT = 3

Monoisotopic Mass, Even Electron Ions
510 formula(e) evaluated with 1 results within limits (up to 50 best isotopic matches for each mass)
Elements Used:
C: 12-12 H: 0-100 N: 0-4 O: 0-8 Na: 0-1 Cl: 1-3 F: 3-4

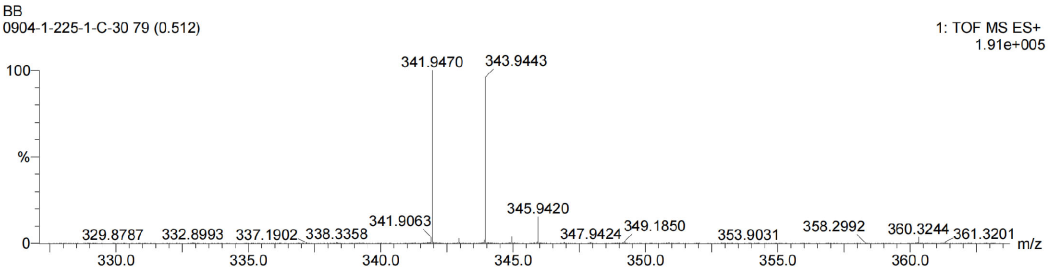

Minimum:				-1.5				
Maximum:		5.0	10.0	50.0				
Mass	Calc. Mass	mDa	PPM	DBE	i-FIT	Norm	Conf(%)	Formula
341.9470	341.9467	0.3	0.9	7.5	757.6	n/a	n/a	C12 H6 N O Cl3 F3

Fig. S21 HRMS spectrum of compound **7**

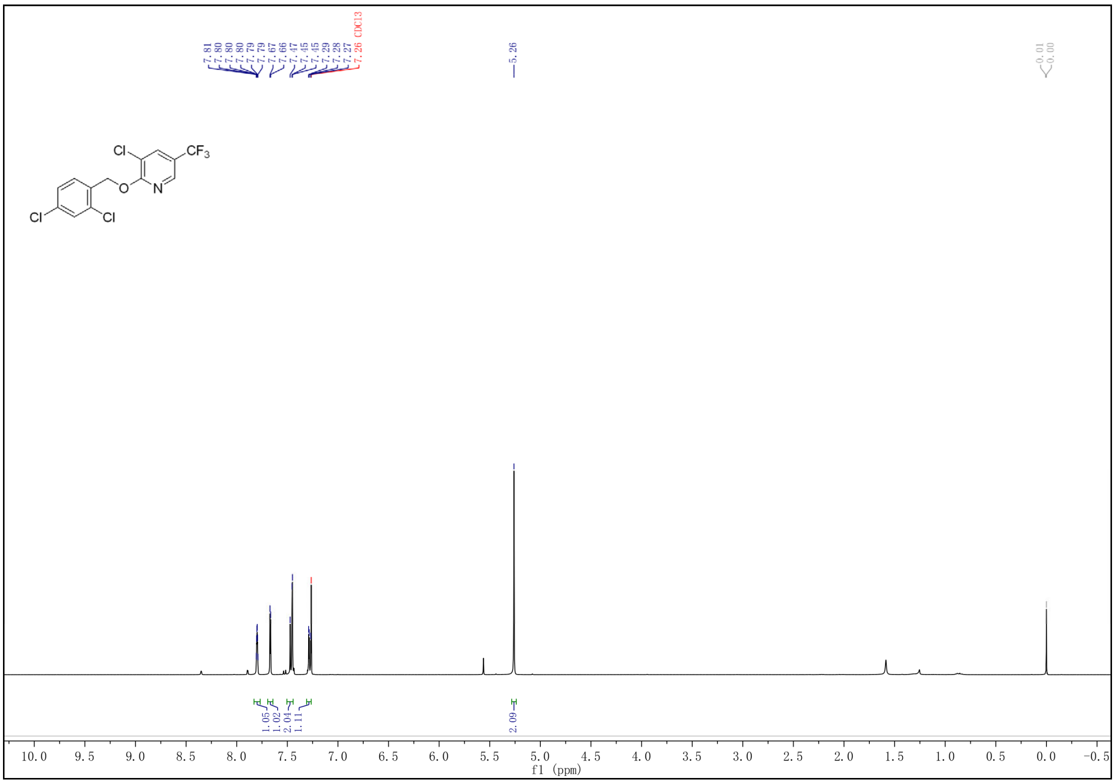

Fig. S22 ^{1}H NMR spectrum of compound **8**

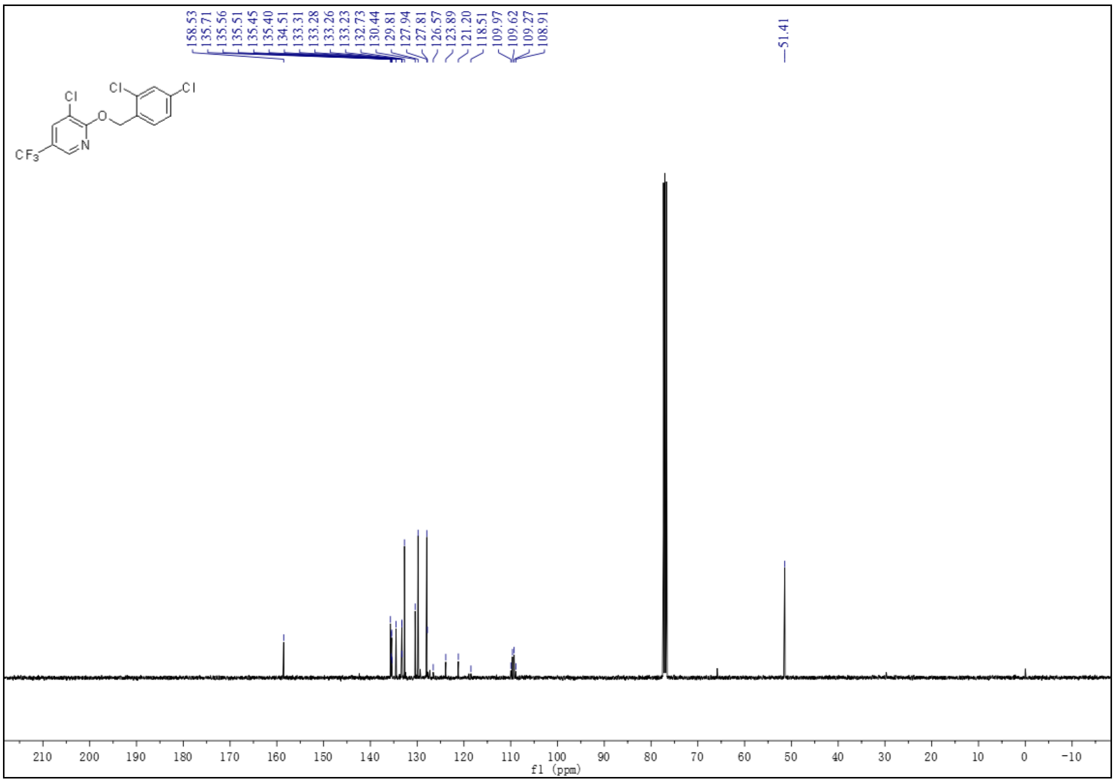

Fig. S23 ^{13}C NMR spectrum of compound **8**

Elemental Composition Report **Page 1**

Single Mass Analysis
Tolerance = 10.0 PPM / DBE: min = -1.5, max = 50.0
Element prediction: Off
Number of isotope peaks used for i-FIT = 3

Monoisotopic Mass, Even Electron Ions
174 formula(e) evaluated with 1 results within limits (up to 50 best isotopic matches for each mass)
Elements Used:
C: 13-13 H: 0-100 N: 0-4 O: 0-8 F: 3-4 Na: 0-1 Cl: 3-3
BB
0904-1-225-1-C-31 53 (0.351) 1: TOF MS ES+
2.72e+006

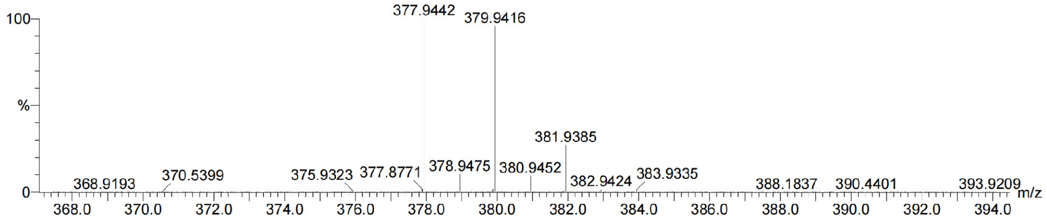

Minimum:				-1.5				
Maximum:		5.0	10.0	50.0				
Mass	Calc. Mass	mDa	PPM	DBE	i-FIT	Norm	Conf(%)	Formula
377.9442	377.9443	-0.1	-0.3	7.5	1217.9	n/a	n/a	C13 H7 N O F3 Na Cl3

Fig. S24 HRMS spectrum of compound **8**

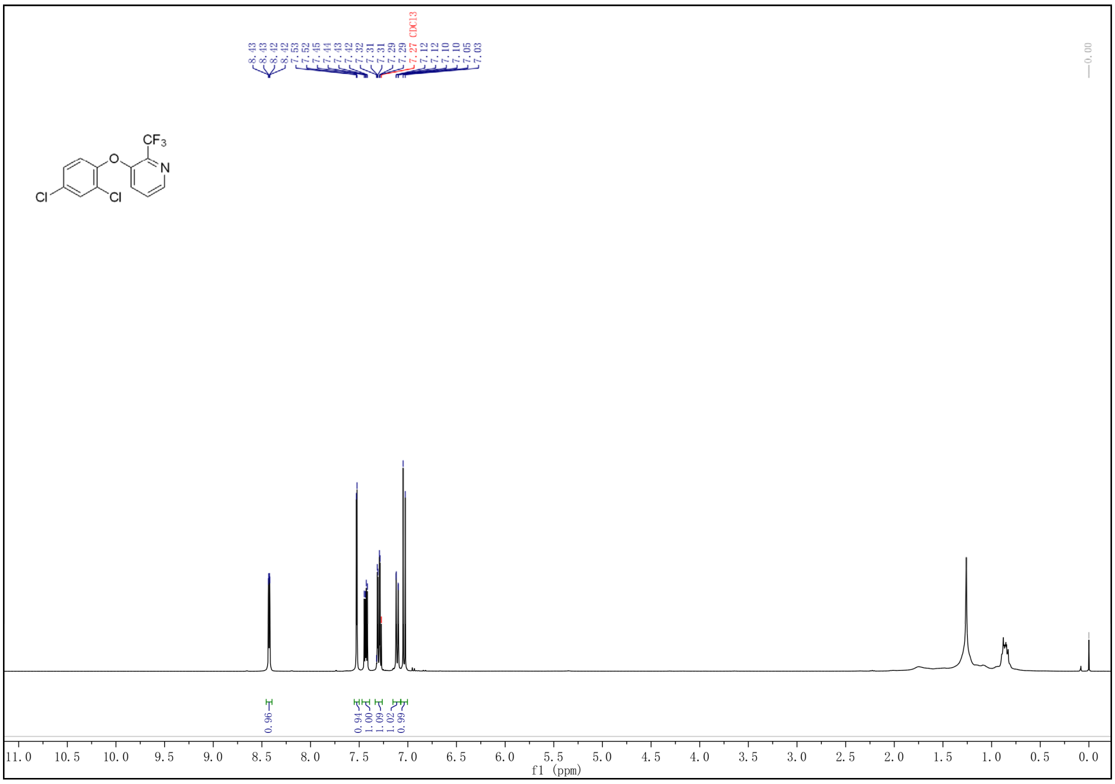

Fig. S25 ^{1}H NMR spectrum of compound **9**

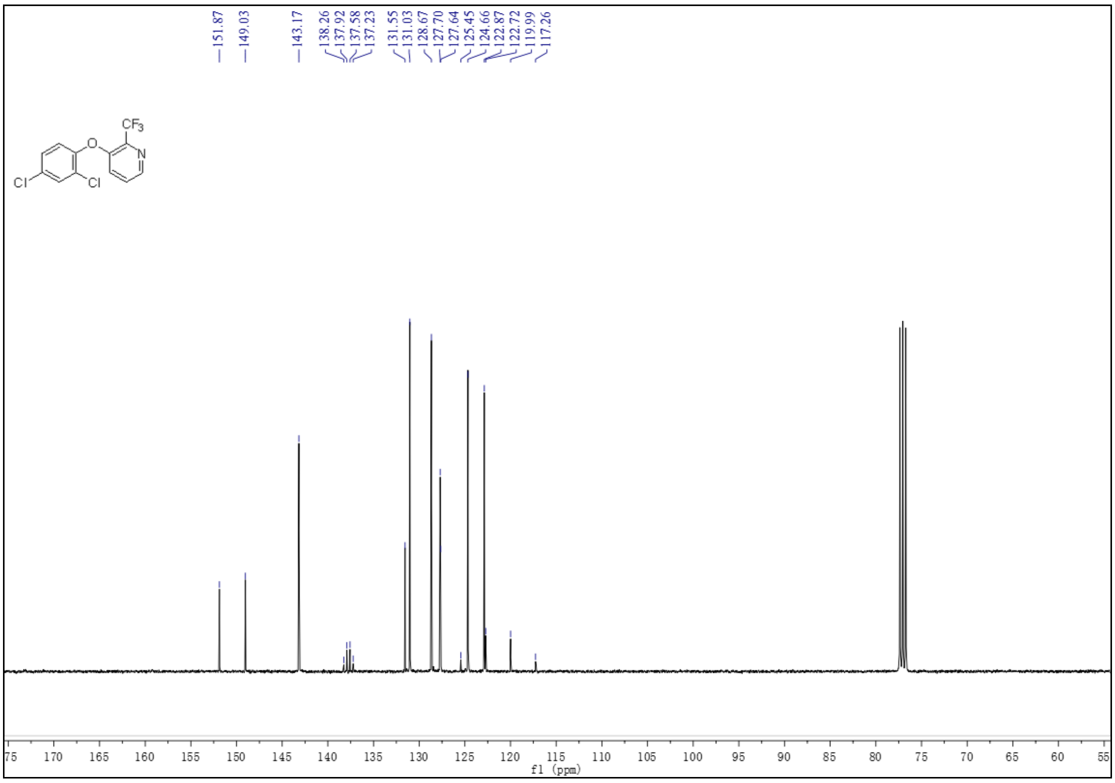

Fig. S26 ^{13}C NMR spectrum of compound **9**

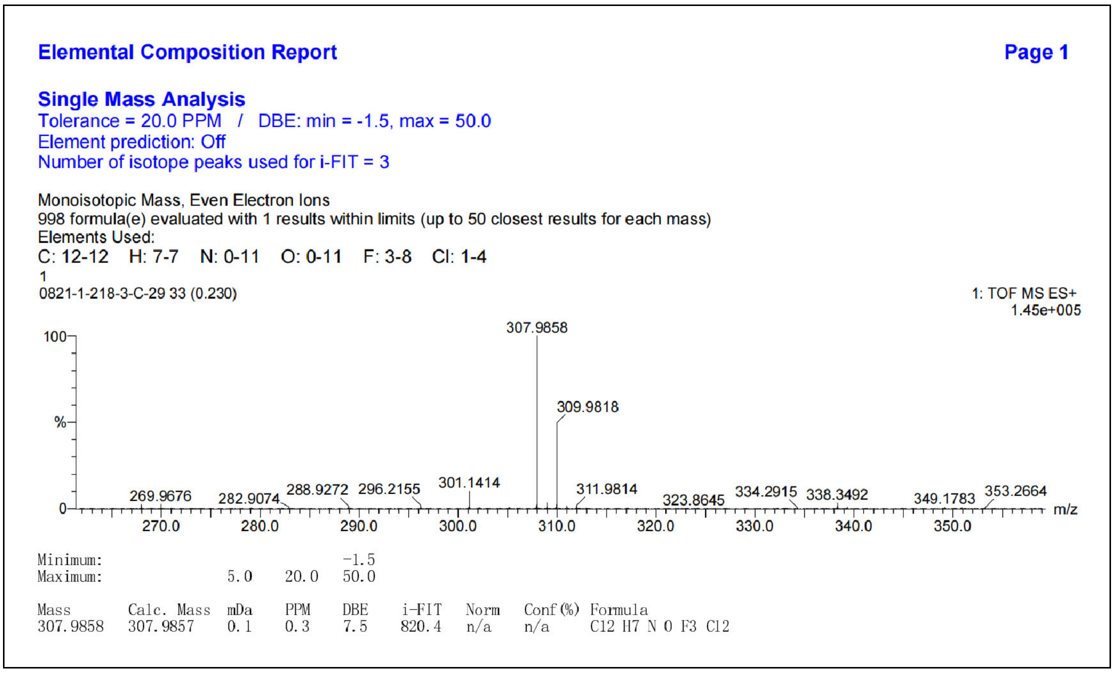

Fig. S27 HRMS spectrum of compound **9**

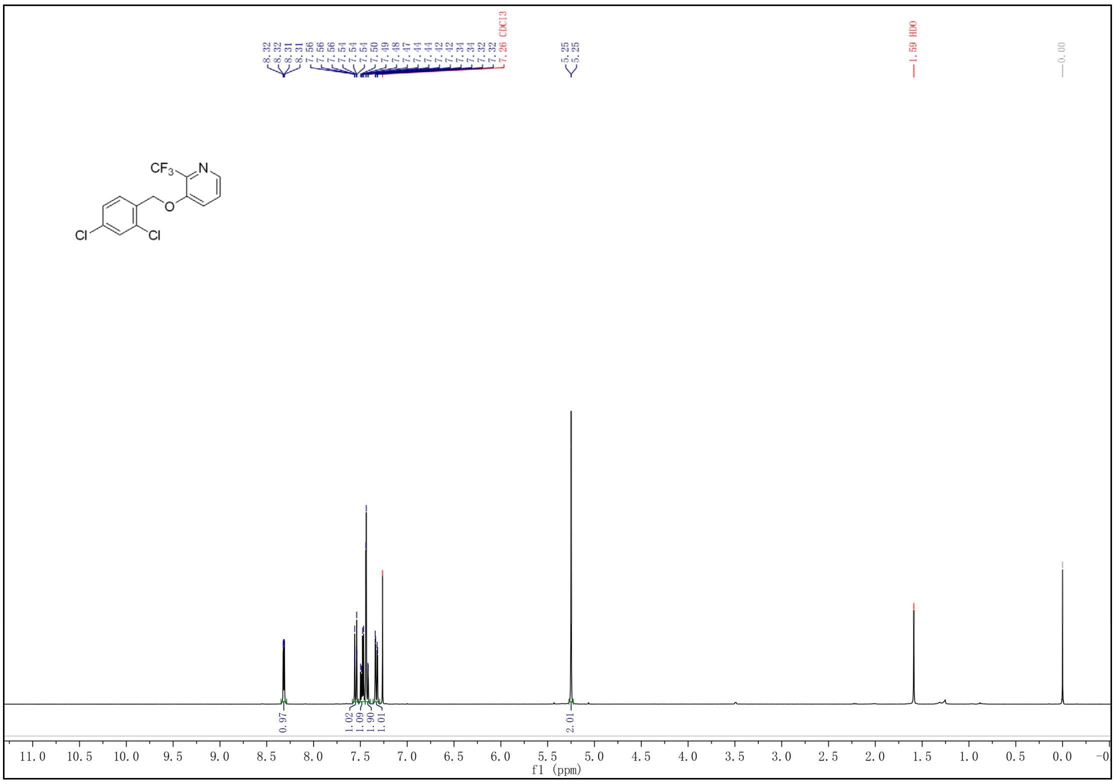

Fig. S28 ^{1}H NMR spectrum of compound **10**

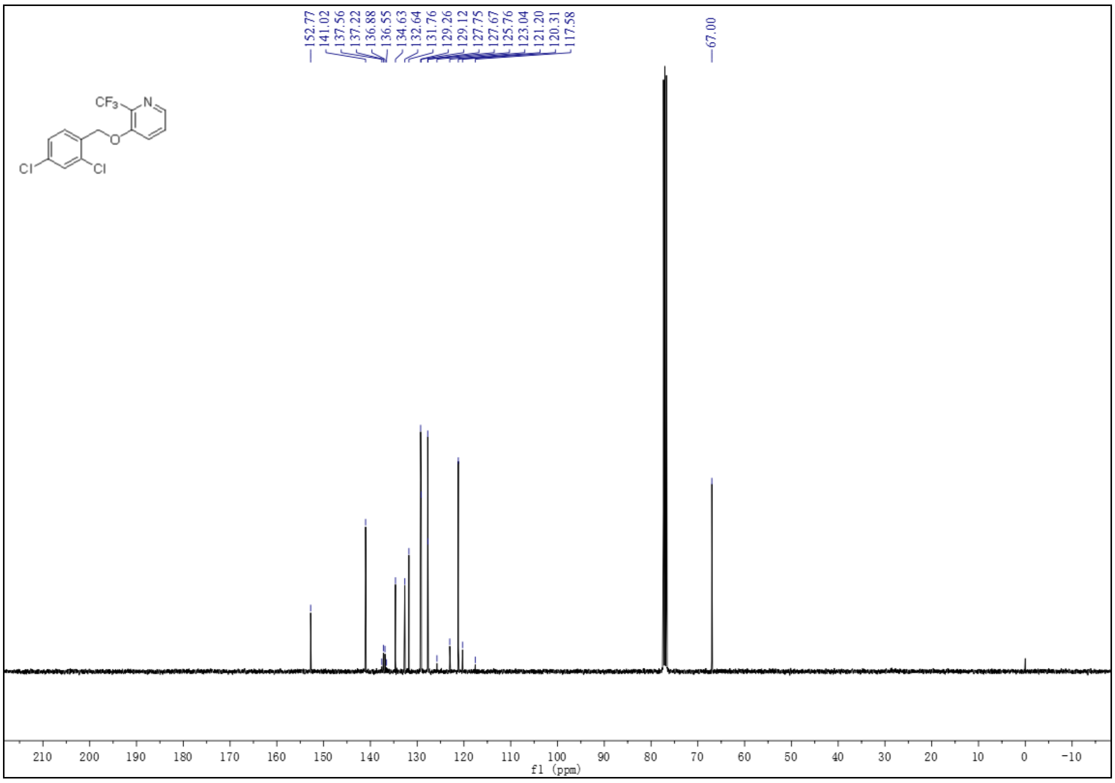

Fig. S29 ^{13}C NMR spectrum of compound **10**

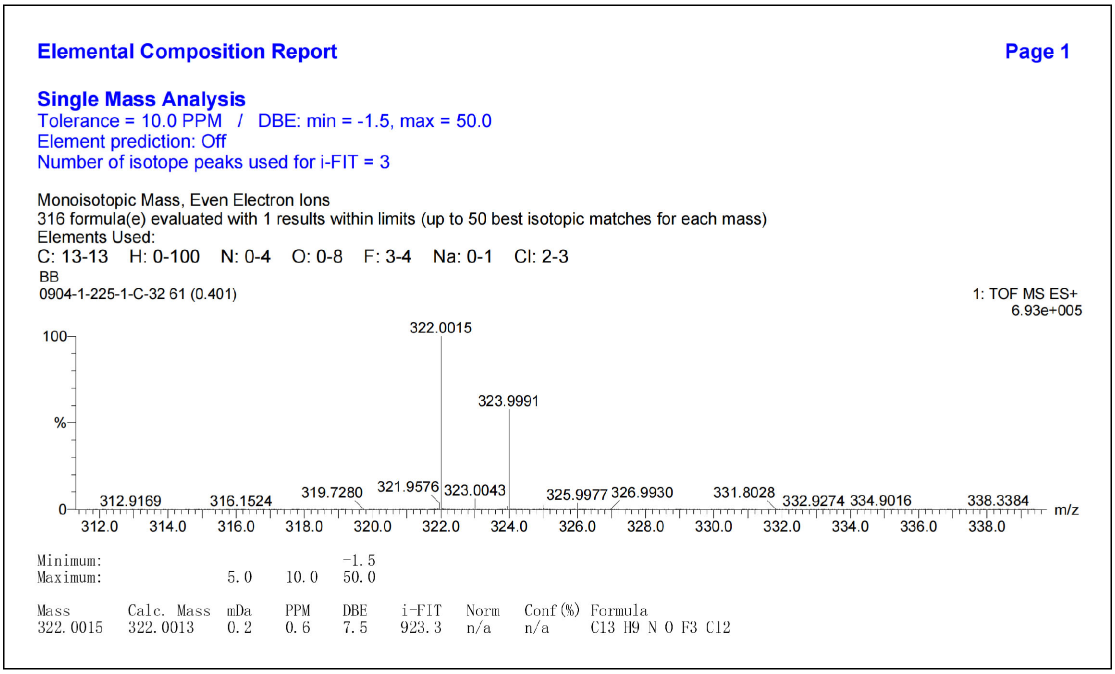

Fig. S30 HRMS spectrum of compound **10**

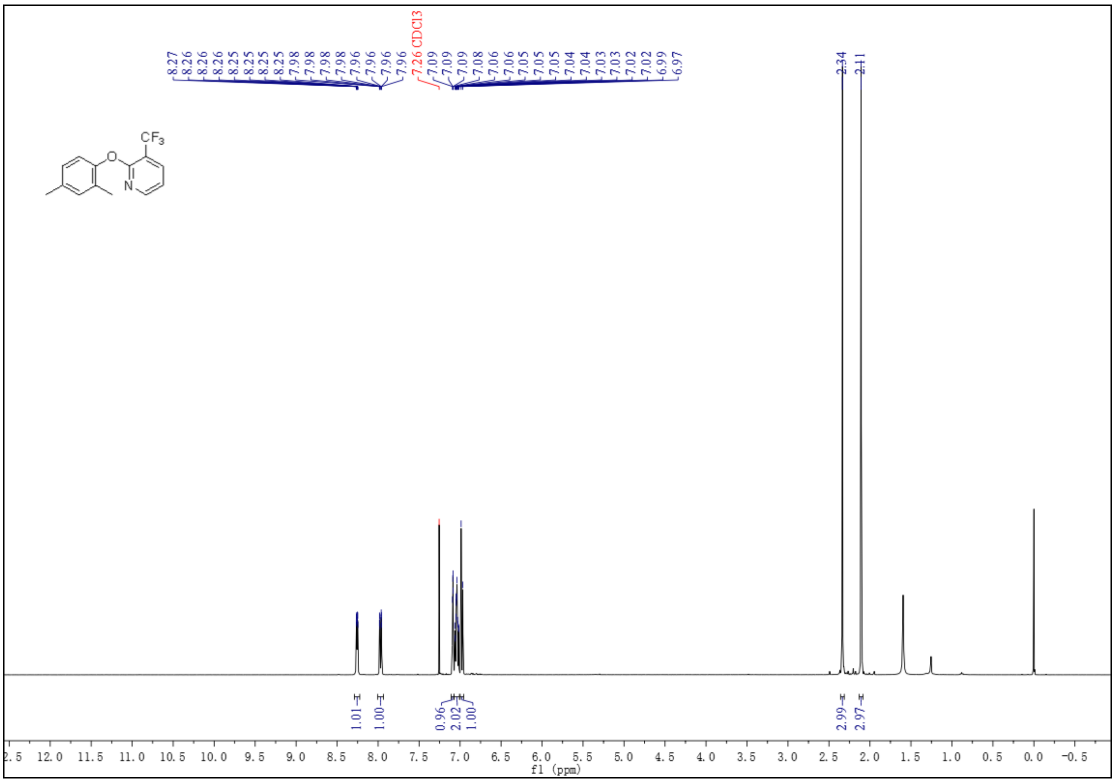

Fig. S31 ^{1}H NMR spectrum of compound **11**

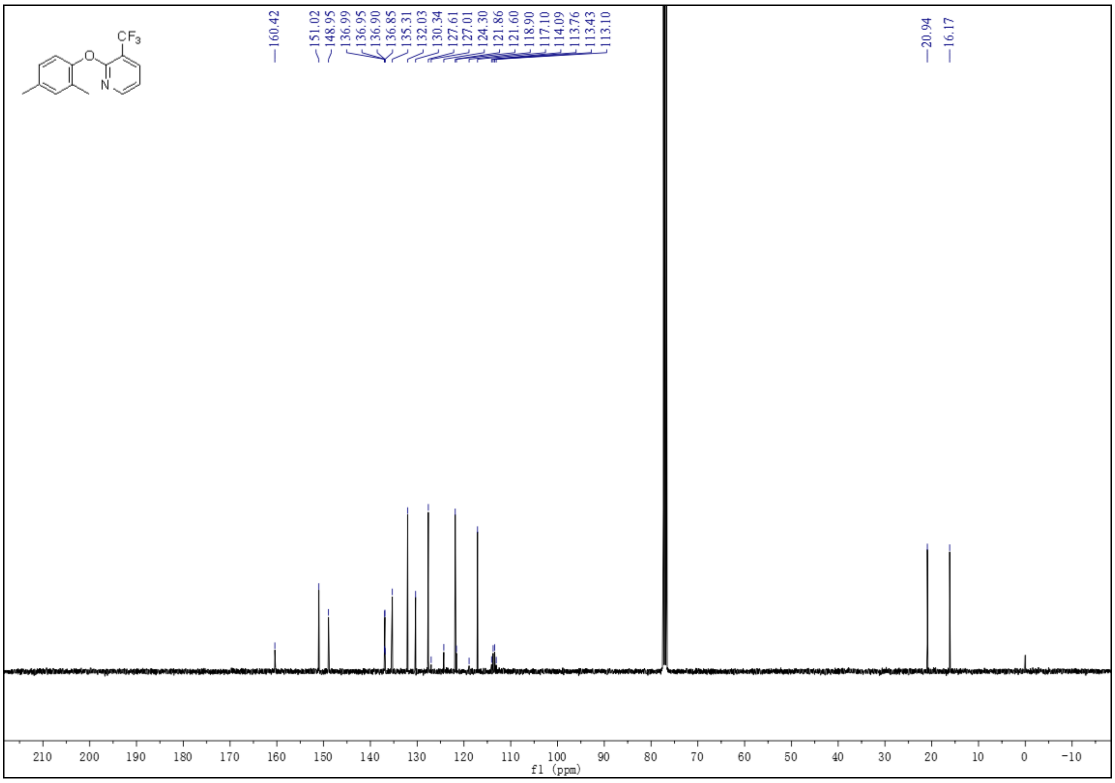

Fig. S32 ^{13}C NMR spectrum of compound **11**

Elemental Composition Report **Page 1**

Single Mass Analysis
Tolerance = 20.0 PPM / DBE: min = -1.5, max = 50.0
Element prediction: Off
Number of isotope peaks used for i-FIT = 3

Monoisotopic Mass, Even Electron Ions
413 formula(e) evaluated with 1 results within limits (up to 50 closest results for each mass)
Elements Used:
C: 14-14 H: 13-13 N: 0-11 O: 0-11 F: 3-8
1
0821-1-218-3-C-33 11 (0.091) 1: TOF MS ES+ 1.09e+007

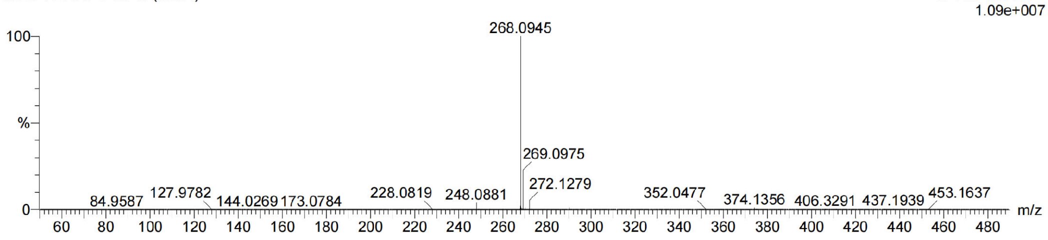

Minimum:				-1.5				
Maximum:		5.0	20.0	50.0				
Mass	Calc. Mass	mDa	PPM	DBE	i-FIT	Norm	Conf(%)	Formula
268.0945	268.0949	-0.4	-1.5	7.5	1382.3	n/a	n/a	C14 H13 N O F3

Fig. S33 HRMS spectrum of compound **11**

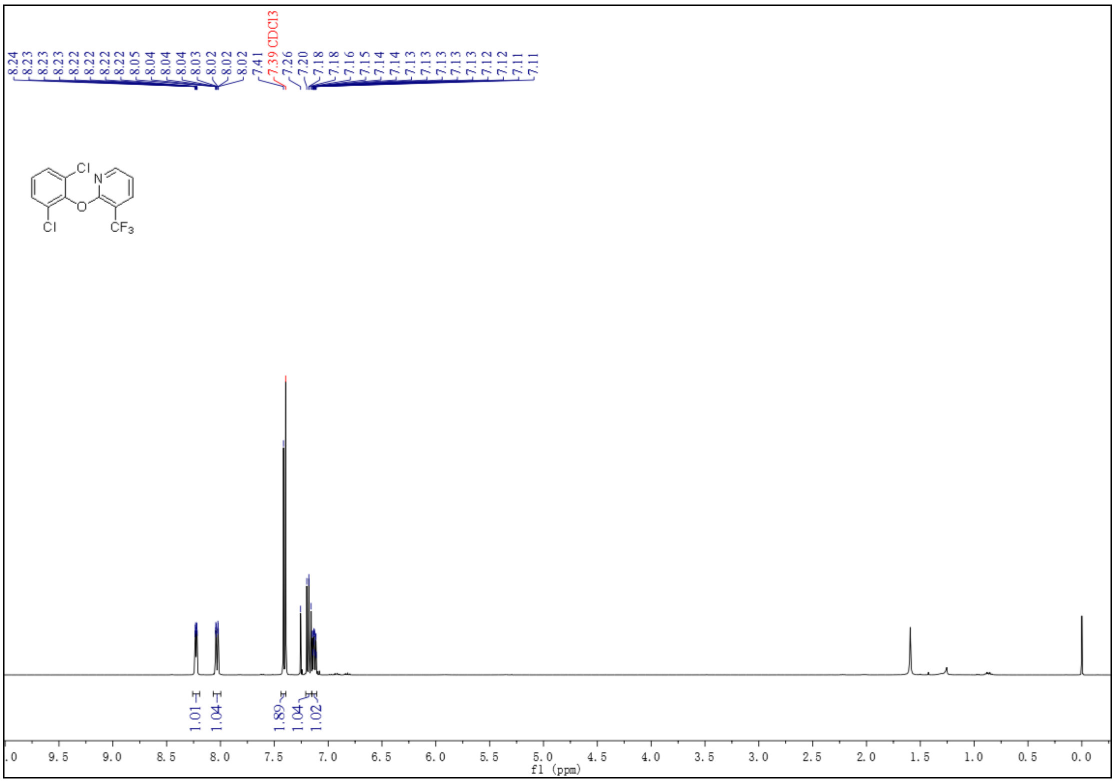

Fig. S34 ^{1}H NMR spectrum of compound **12**

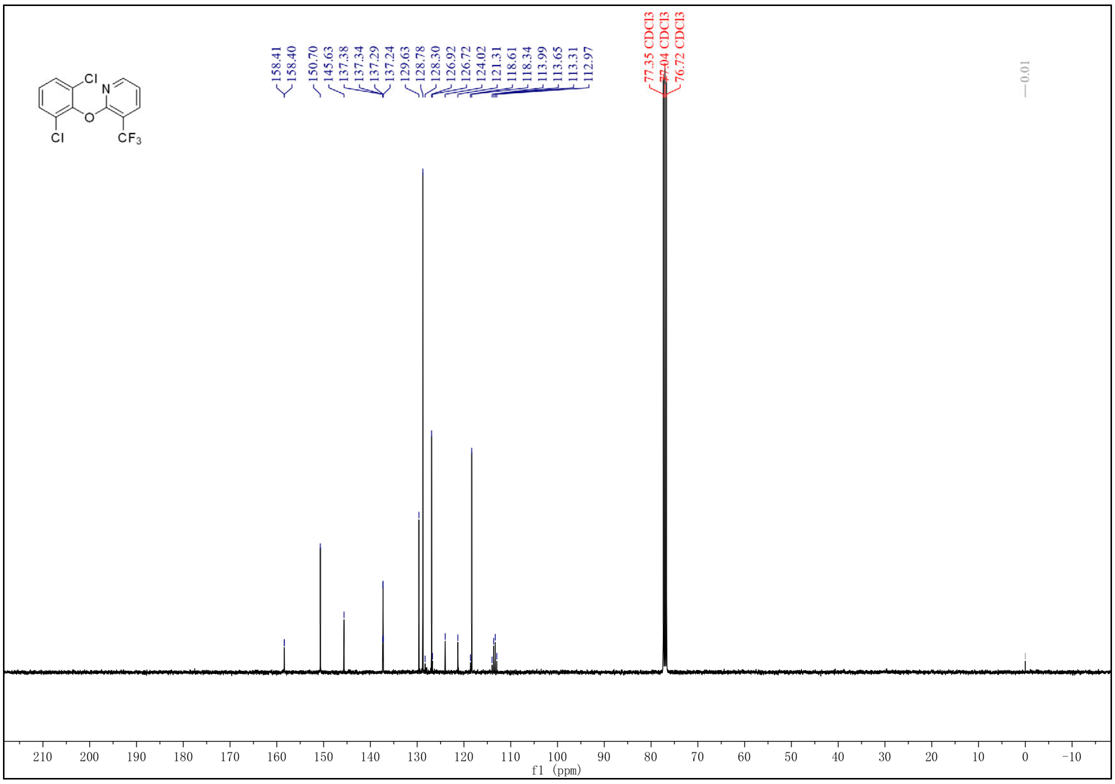

Fig. S35 ^{13}C NMR spectrum of compound **12**

Elemental Composition Report **Page 1**

Single Mass Analysis
Tolerance = 20.0 PPM / DBE: min = -1.5, max = 50.0
Element prediction: Off
Number of isotope peaks used for i-FIT = 3

Monoisotopic Mass, Even Electron Ions
998 formula(e) evaluated with 1 results within limits (up to 50 closest results for each mass)
Elements Used:
C: 12-12 H: 7-7 N: 0-11 O: 0-11 F: 3-8 Cl: 1-4
1
0821-1-218-3-C-36 11 (0.091) 1: TOF MS ES+
4.82e+006

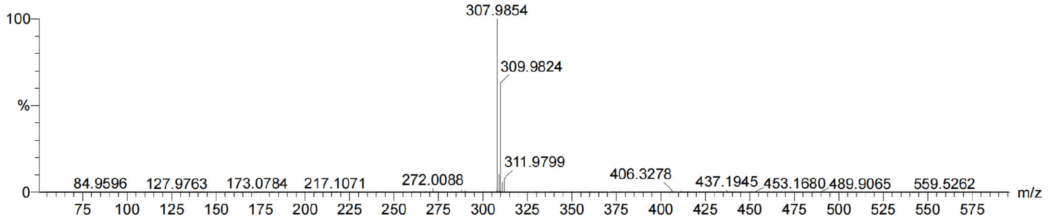

Minimum:				-1.5				
Maximum:		5.0	20.0	50.0				
Mass	Calc. Mass	mDa	PPM	DBE	i-FIT	Norm	Conf(%)	Formula
307.9854	307.9857	-0.3	-1.0	7.5	1366.7	n/a	n/a	C12 H7 N O F3 Cl2

Fig. S36 HRMS spectrum of compound **12**

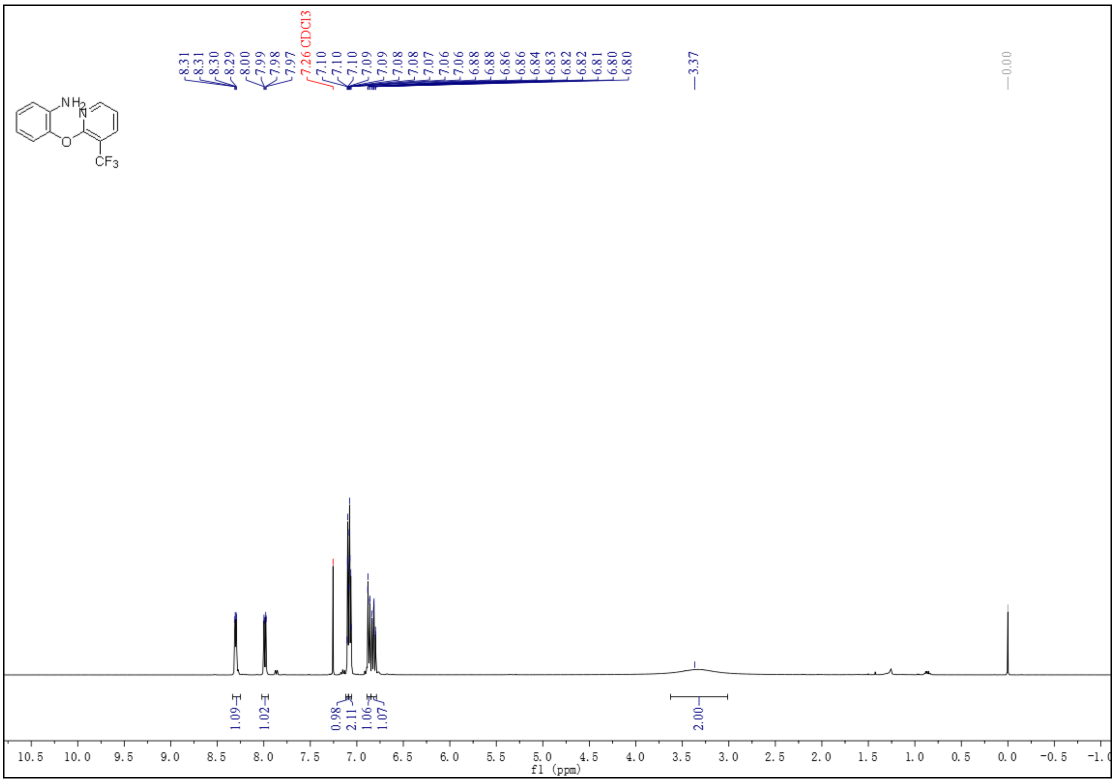

Fig. S37 ^{1}H NMR spectrum of compound **13**

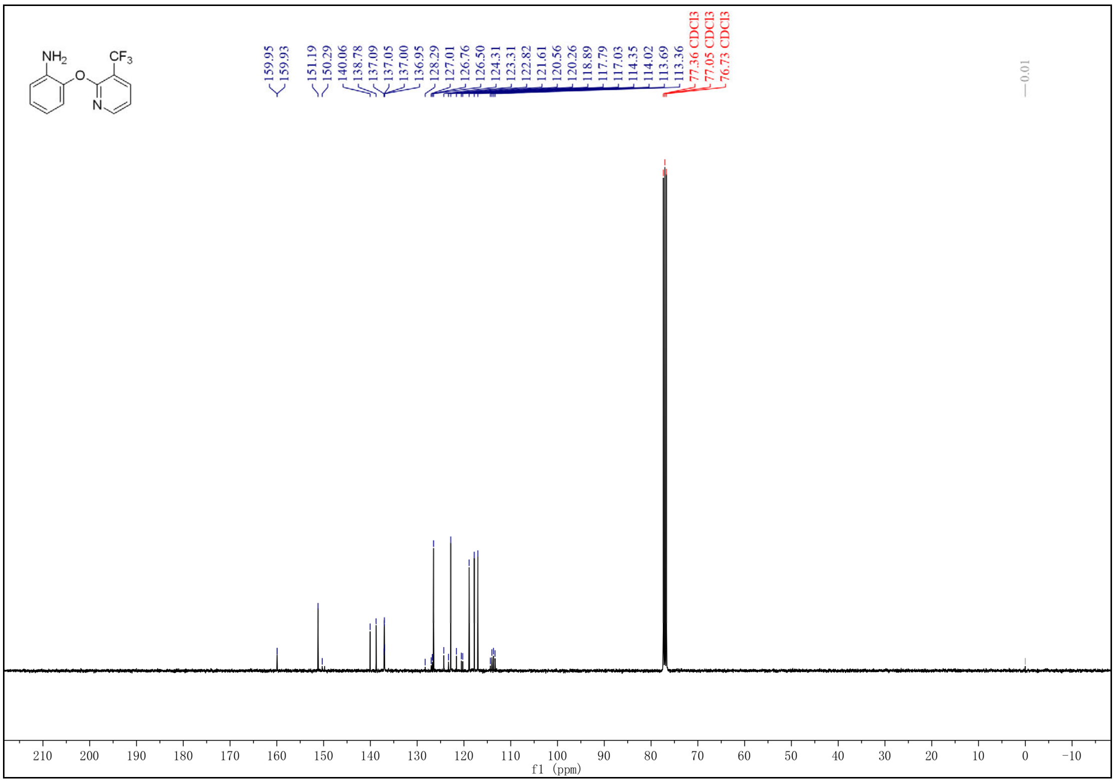

Fig. S38 ^{13}C NMR spectrum of compound **13**

Elemental Composition Report **Page 1**

Single Mass Analysis
Tolerance = 10.0 PPM / DBE: min = -1.5, max = 50.0
Element prediction: Off
Number of isotope peaks used for i-FIT = 3

Monoisotopic Mass, Even Electron Ions
86 formula(e) evaluated with 1 results within limits (up to 50 best isotopic matches for each mass)
Elements Used:
C: 12-12 H: 0-100 N: 0-4 O: 0-8 F: 3-3 Na: 0-1
BB
0904-1-225-1-C-67 82 (0.536) 1: TOF MS ES+ 1.64e+004

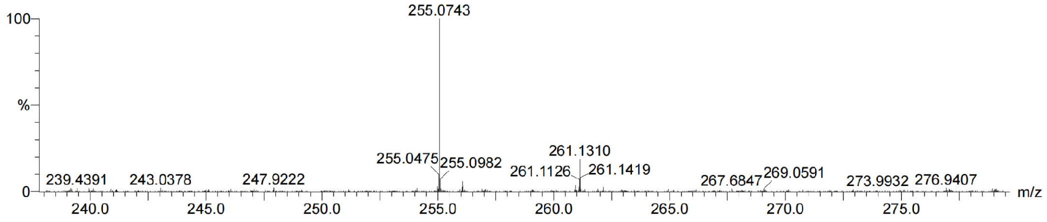

Minimum:				-1.5
Maximum:		5.0	10.0	50.0

Mass	Calc. Mass	mDa	PPM	DBE	i-FIT	Norm	Conf(%)	Formula
255.0743	255.0745	-0.2	-0.8	7.5	484.6	n/a	n/a	C12 H10 N2 O F3

Fig. S39 HRMS spectrum of compound **13**

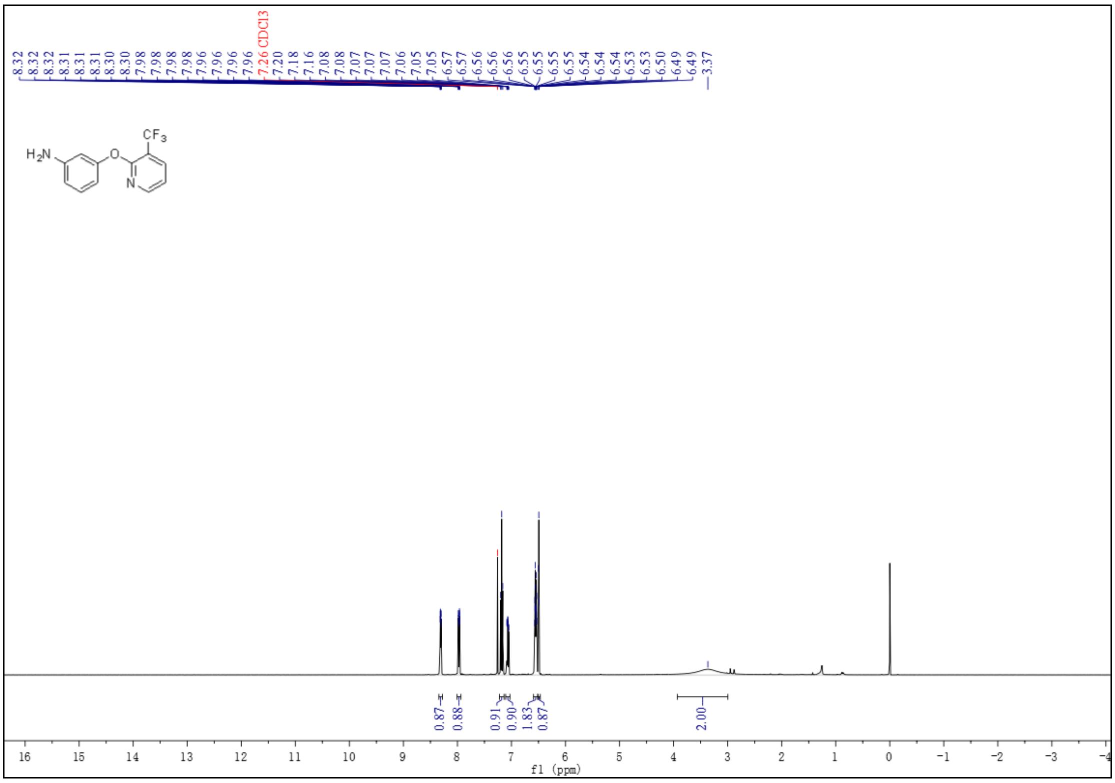

Fig. S40 ^{1}H NMR spectrum of compound **14**

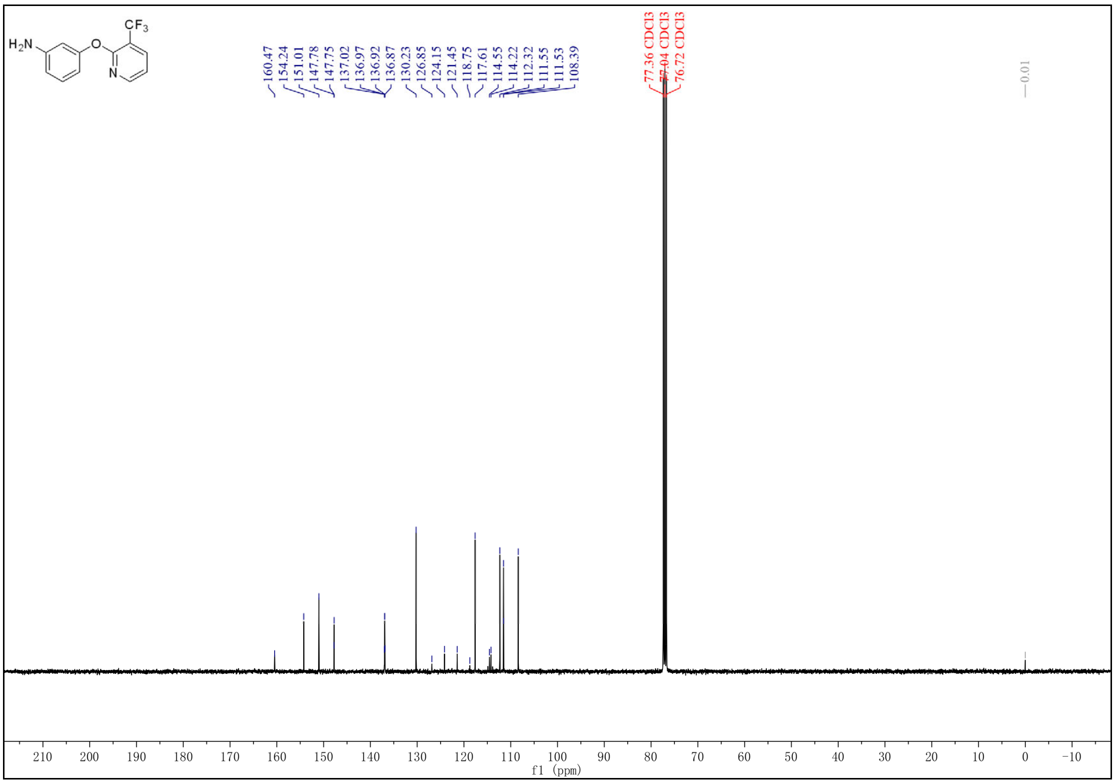

Fig. S41 ^{13}C NMR spectrum of compound **14**

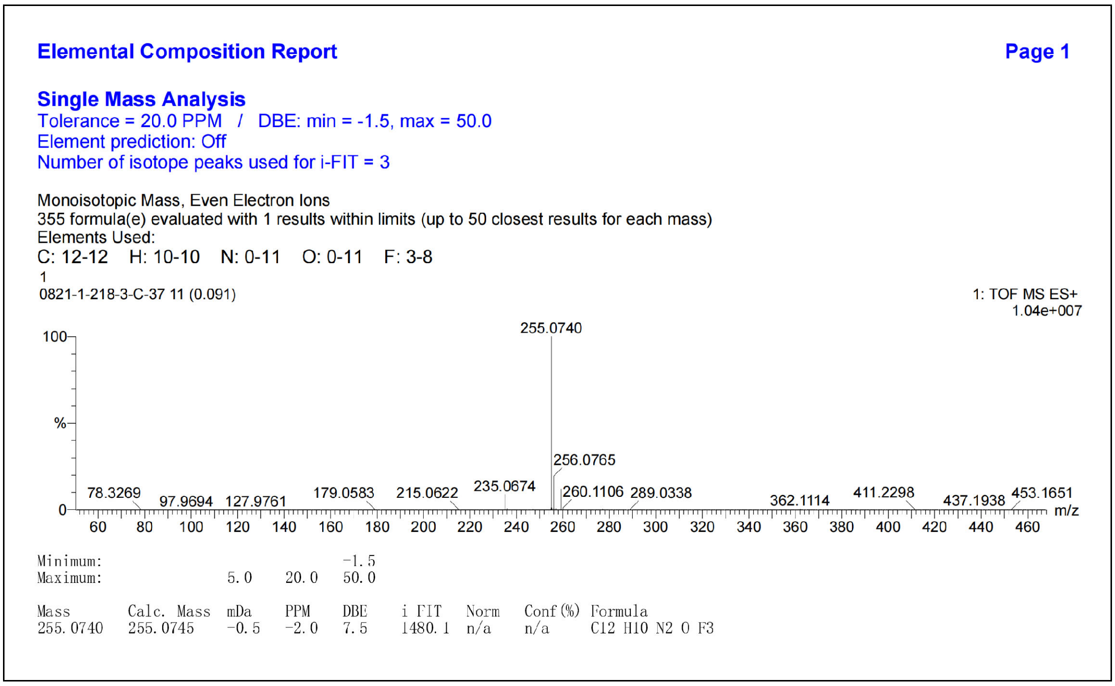

Fig. S42 HRMS spectrumof compound **14**

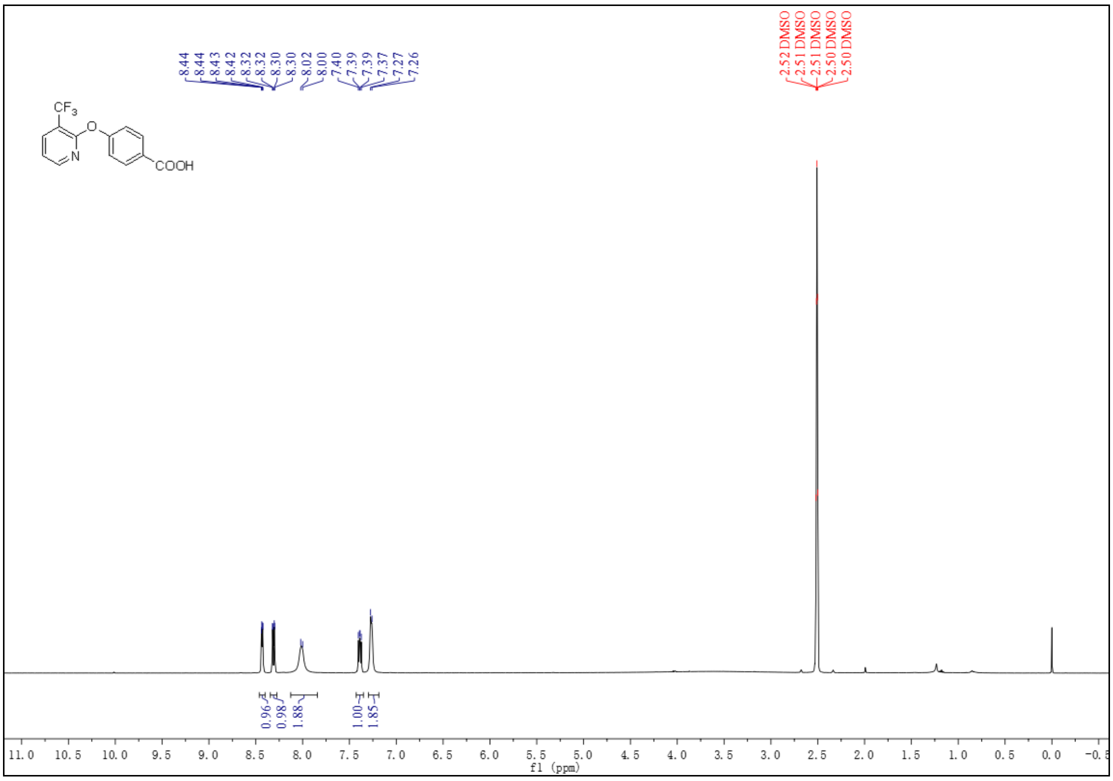

Fig. S43 ^{1}H NMR spectrum of compound **15**

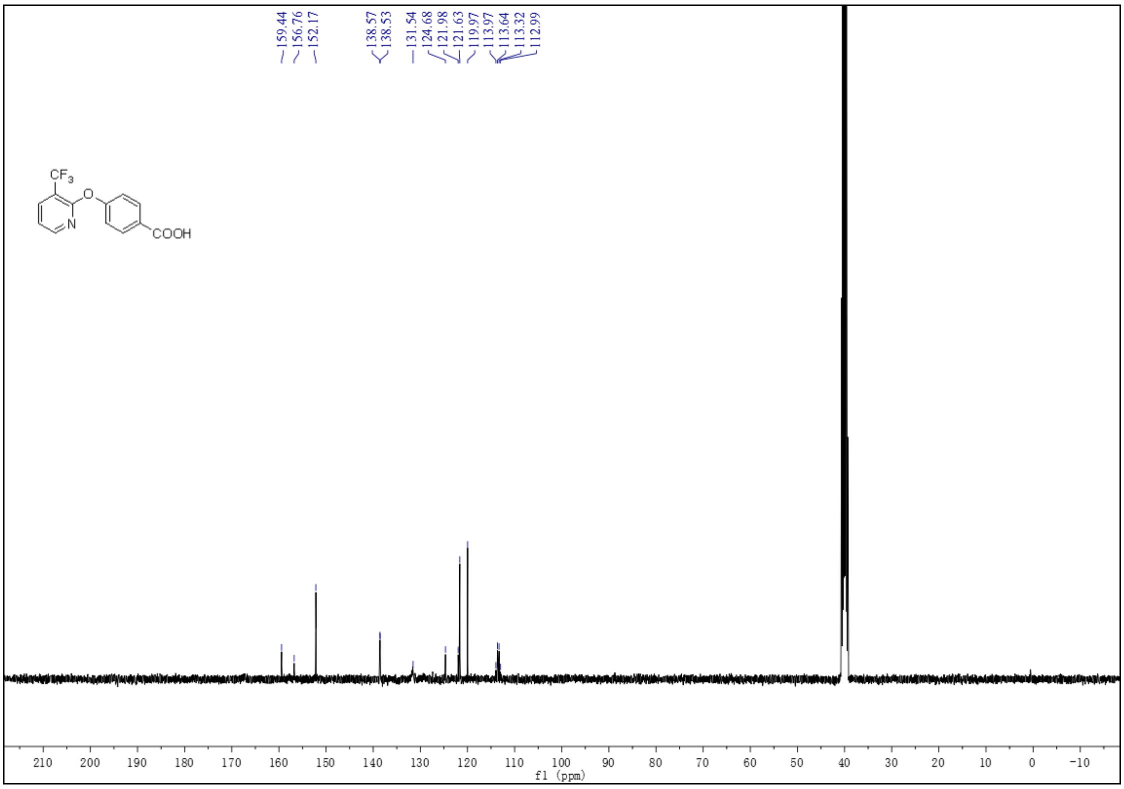

Fig. S44 ^{13}C NMR spectrum of compound **15**

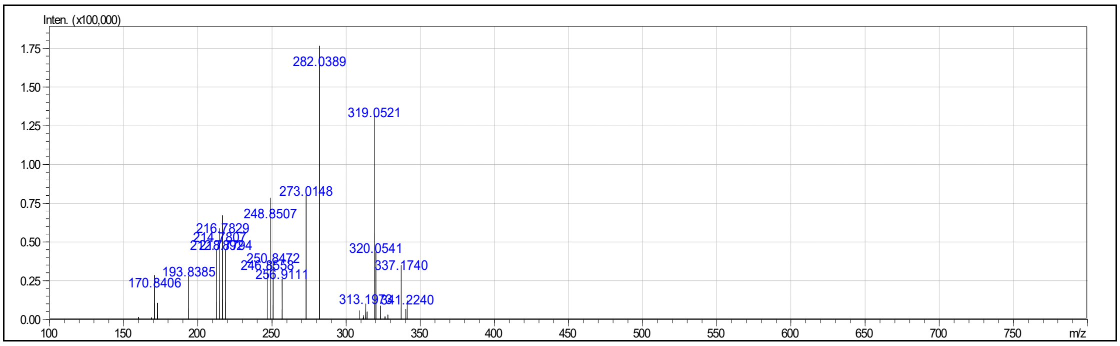

Fig. S45 HRMS spectrum of compound **15**

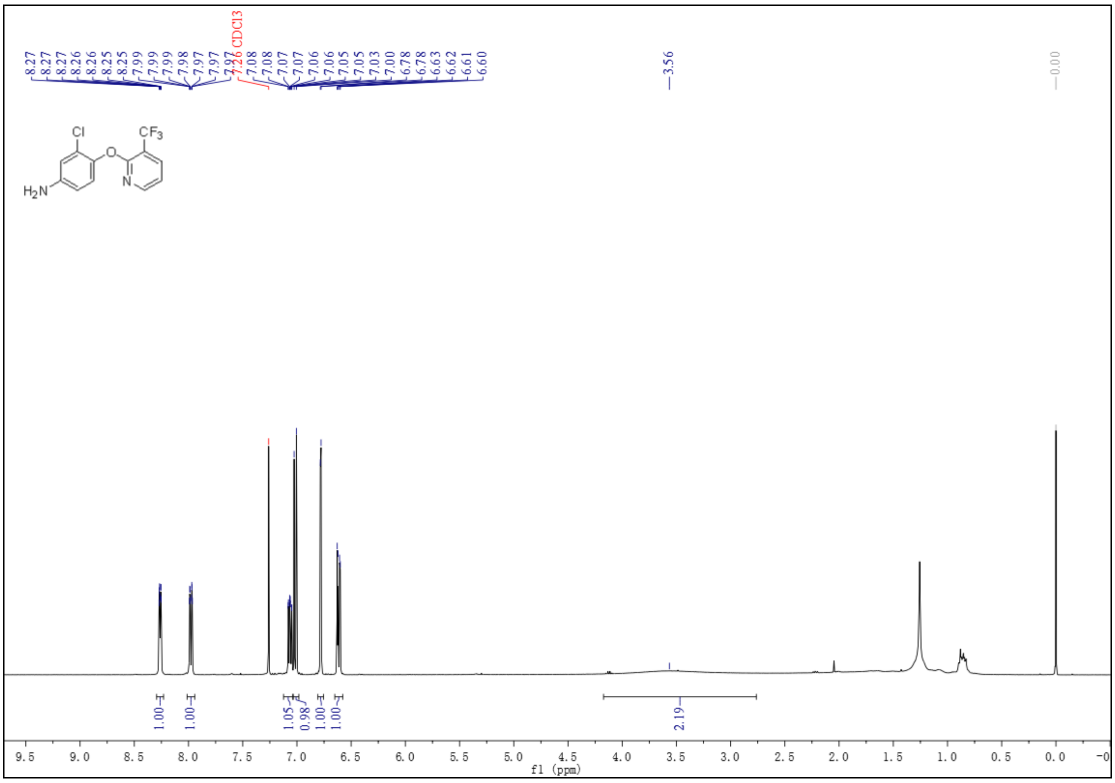

Fig. S46 ^{13}C NMR spectrum of compound **16**

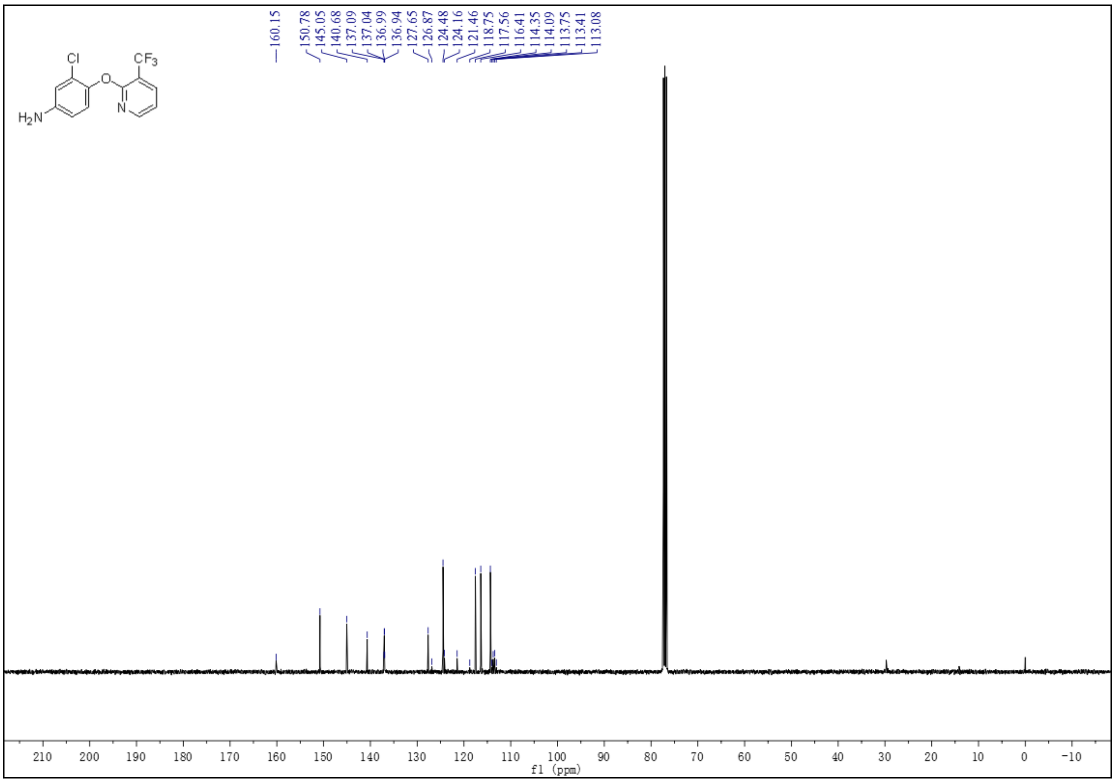

Fig. S47 ^{13}C NMR spectrum of compound **16**

Elemental Composition Report **Page 1**

Single Mass Analysis
Tolerance = 20.0 PPM / DBE: min = -1.5, max = 50.0
Element prediction: Off
Number of isotope peaks used for i-FIT = 3

Monoisotopic Mass, Even Electron Ions
1349 formula(e) evaluated with 1 results within limits (up to 50 closest results for each mass)
Elements Used:
C: 12-12 H: 9-9 N: 0-11 O: 0-11 F: 1-8 Cl: 1-4
2
0821-1-218-3-C-54 13 (0.101) 1: TOF MS ES+
9.73e+006

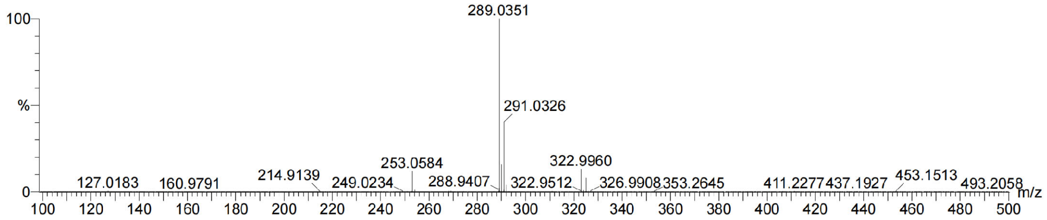

Minimum:				-1.5				
Maximum:		5.0	20.0	50.0				
Mass	Calc. Mass	mDa	PPM	DBE	i-FIT	Norm	Conf(%)	Formula
289.0351	289.0356	-0.5	-1.7	7.5	1525.0	n/a	n/a	C12 H9 N2 O F3 Cl

Fig. S48 HRMS spectrum of compound **16**

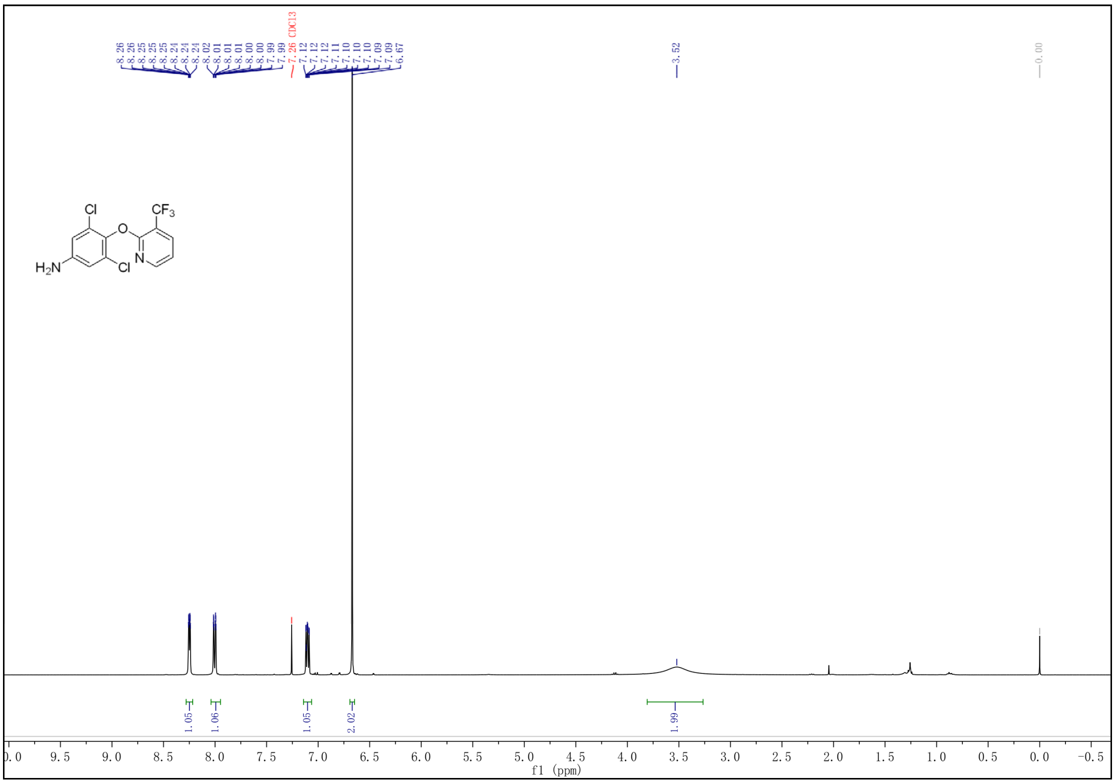

Fig. S49 ^{1}H NMR spectrum of compound **17**

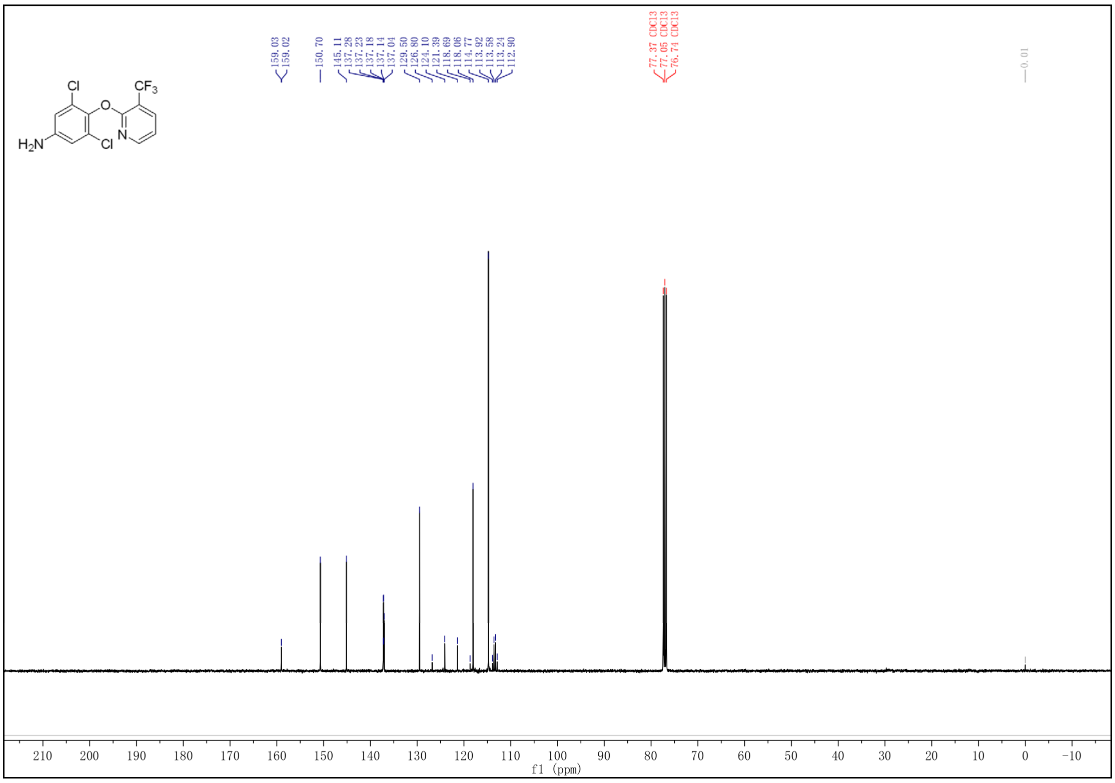

Fig. S50 ^{13}C NMR spectrum of compound **17**

Elemental Composition Report **Page 1**

Single Mass Analysis
Tolerance = 20.0 PPM / DBE: min = -1.5, max = 50.0
Element prediction: Off
Number of isotope peaks used for i-FIT = 3

Monoisotopic Mass, Even Electron Ions
1861 formula(e) evaluated with 1 results within limits (up to 50 closest results for each mass)
Elements Used:
C: 12-12 H: 8-8 N: 0-11 O: 0-11 F: 1-8 Cl: 1-4
2
0821-1-218-3-C-53 8 (0.067) 1: TOF MS ES+ 7.80e+006

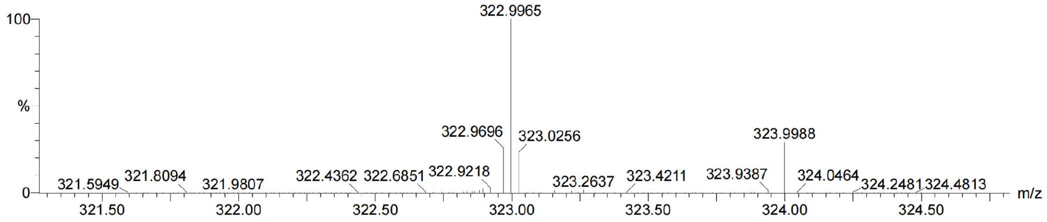

	Mass	Calc. Mass	mDa	PPM	DBE	i-FIT	Norm	Conf(%)	Formula
Minimum:					-1.5				
Maximum:			5.0	20.0	50.0				
	322.9965	322.9966	-0.1	-0.3	7.5	836.1	n/a	n/a	C12 H8 N2 O F3 Cl2

Fig. S51 HRMS spectrum of compound **17**

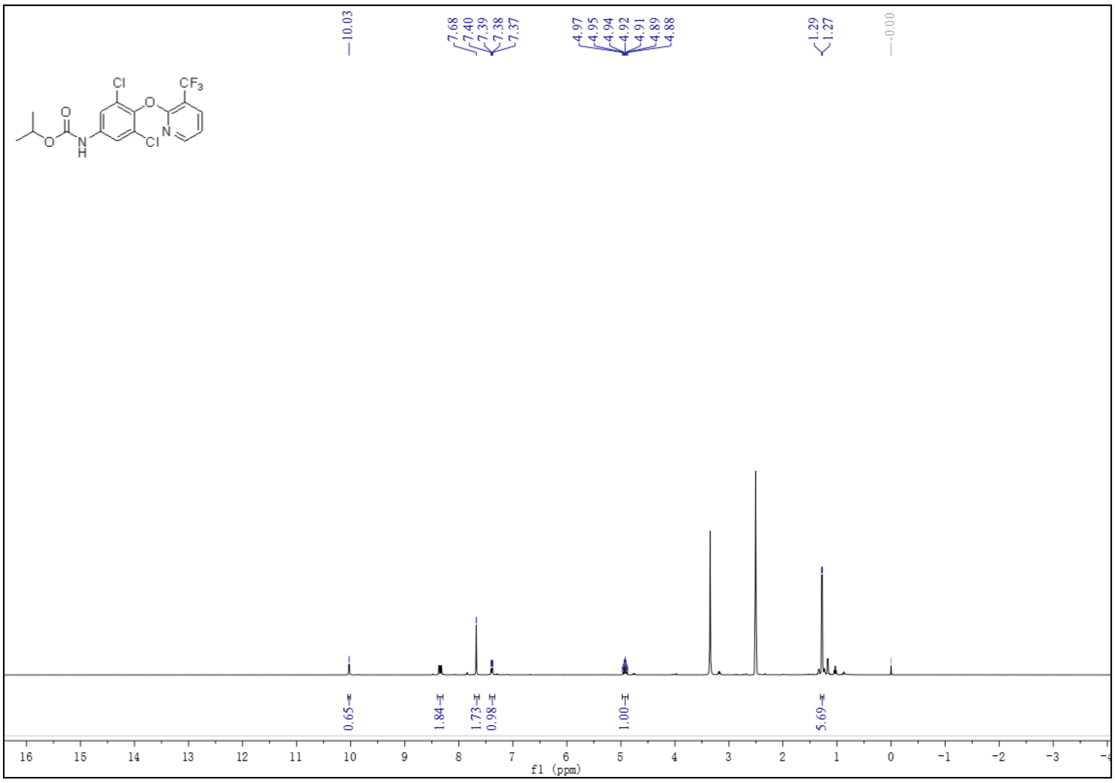

Fig. S52 ^{1}H NMR spectrum of compound **18**

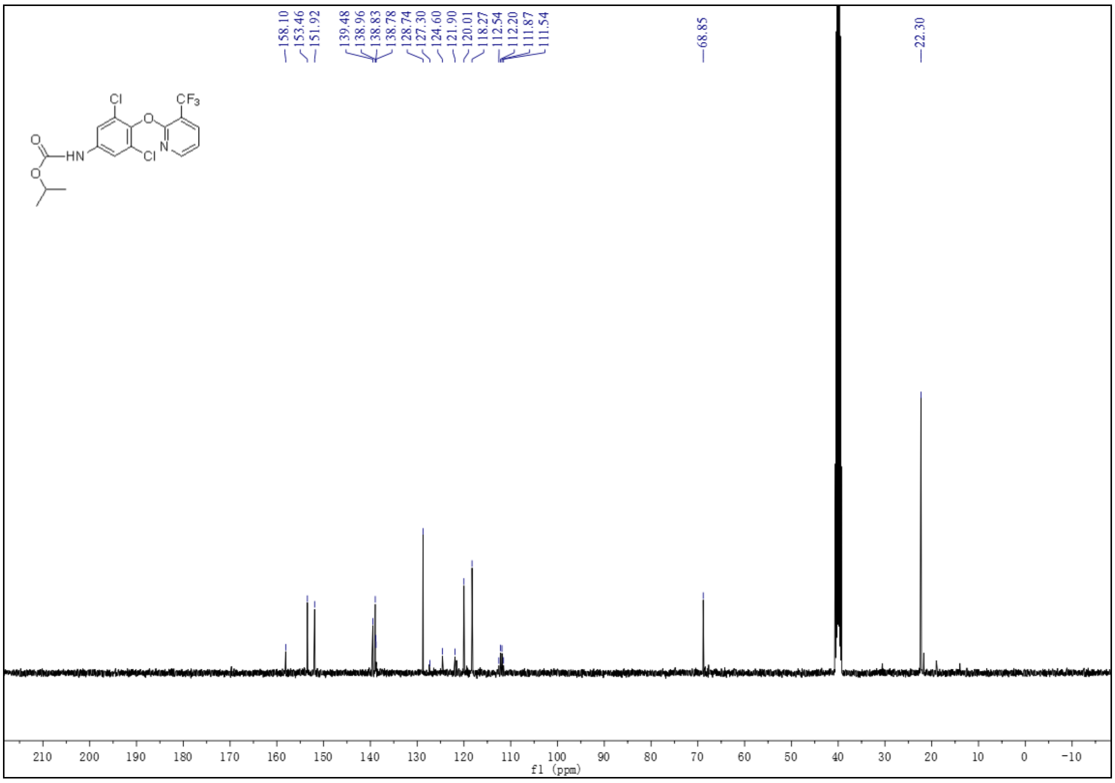

Fig. S53 ^{13}C NMR spectrum of compound **18**

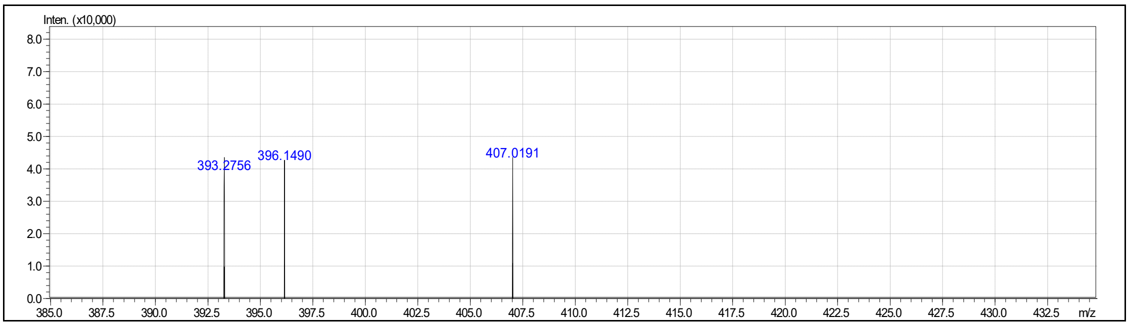

Fig. S54 HRMS spectrum of compound **18**

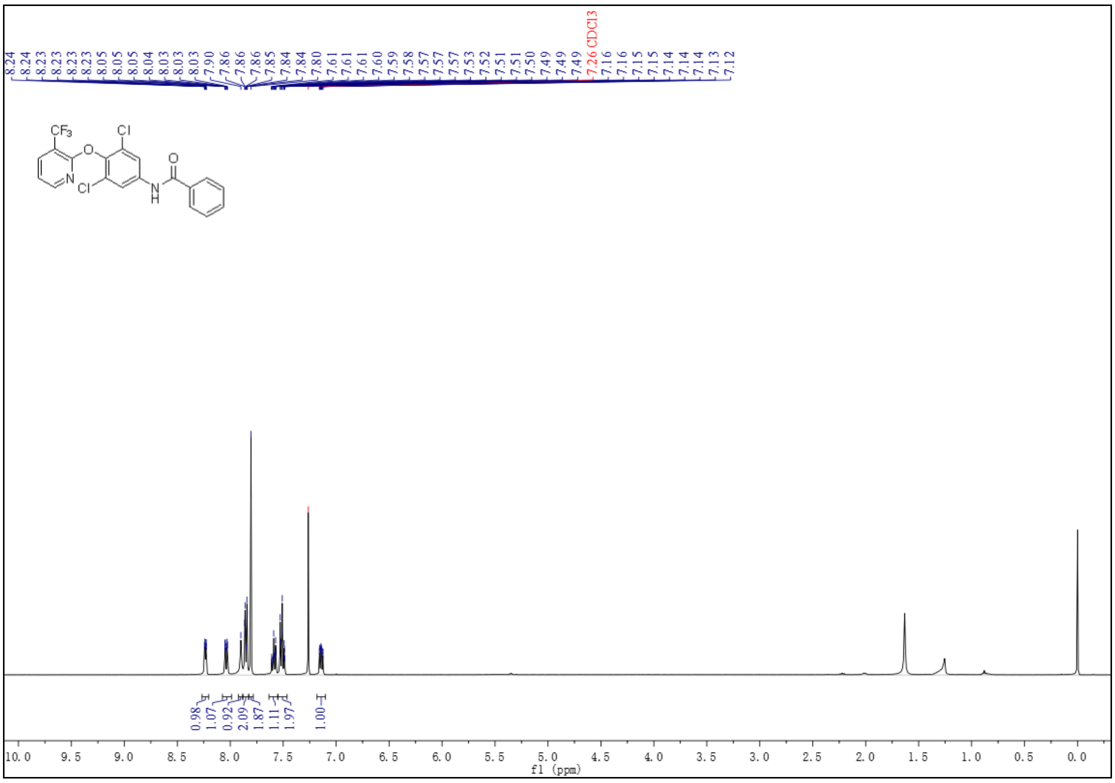

Fig. S55 ^{1}H NMR spectrum of compound **19**

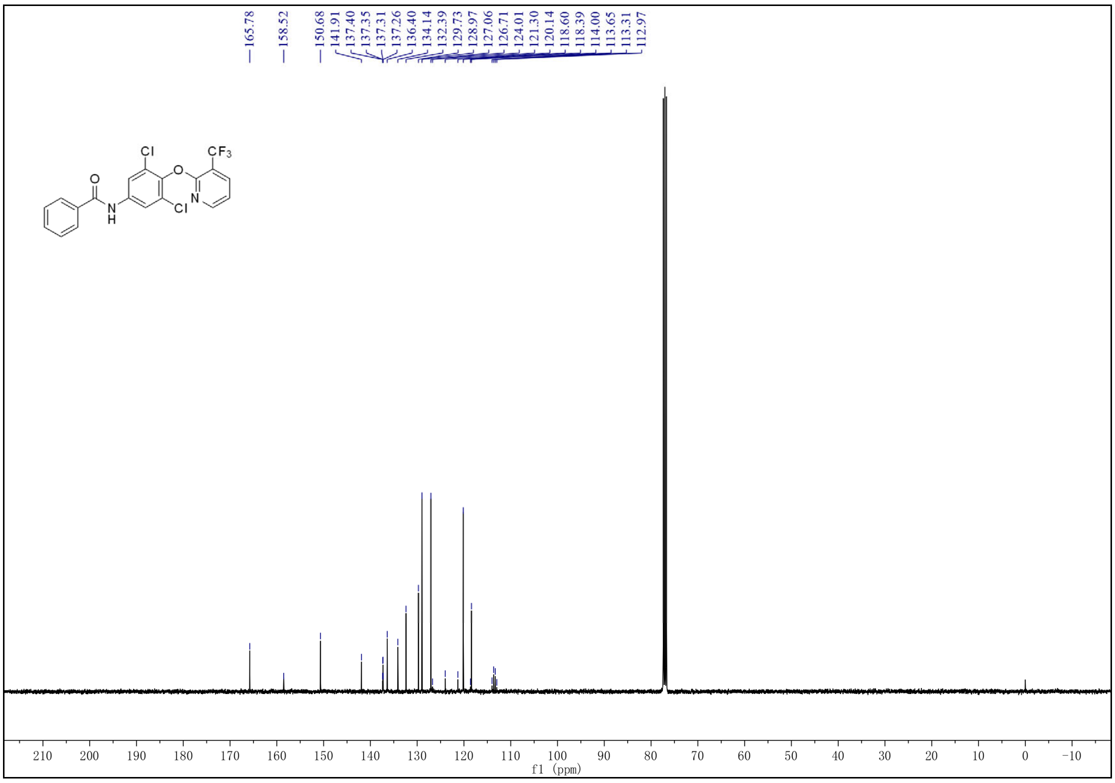

Fig. S56 ^{13}C NMR spectrum of compound **19**

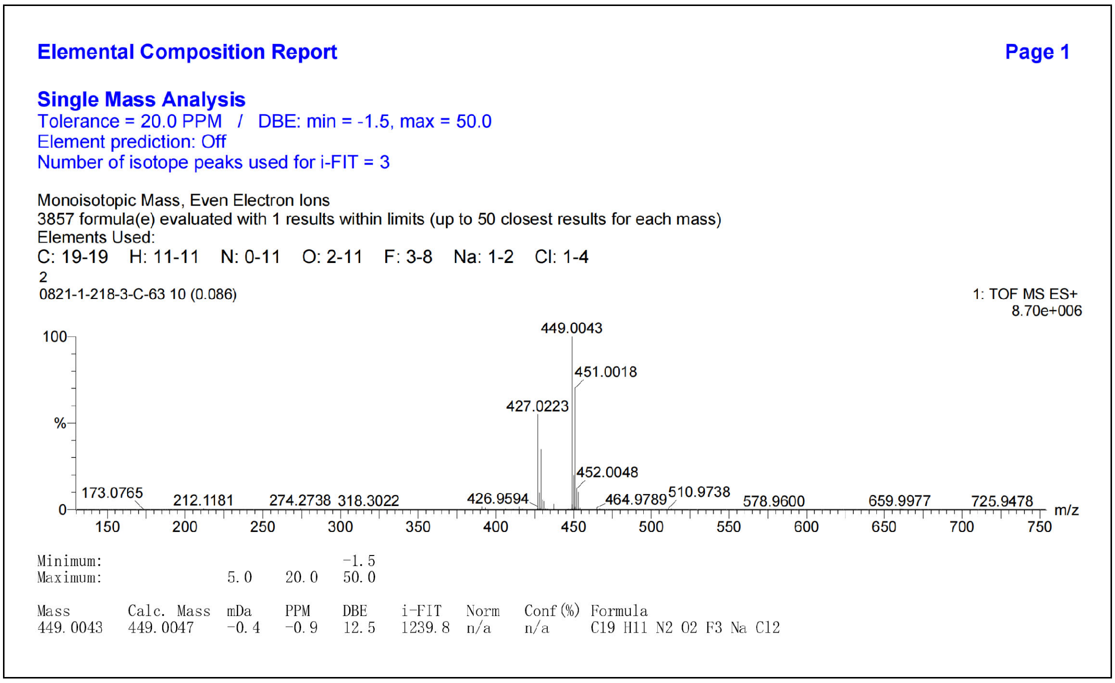

Fig. S57 HRMS spectrum of compound **19**

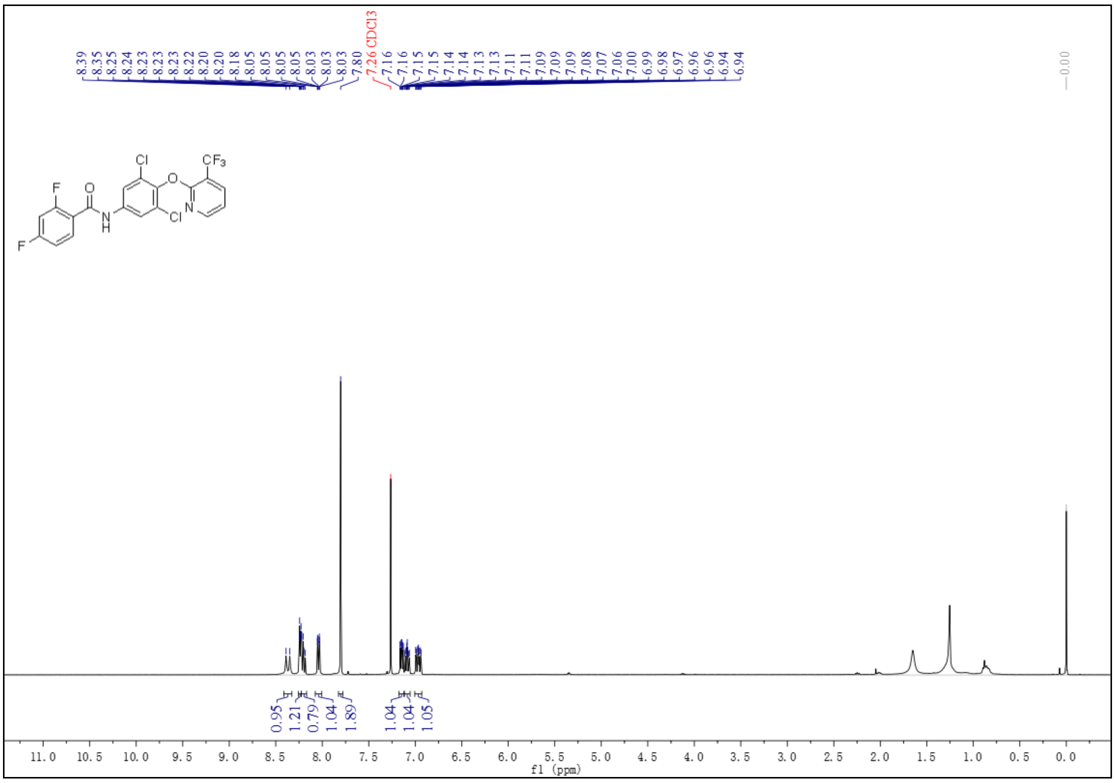

Fig. S58 ^{1}H NMR spectrum of compound **20**

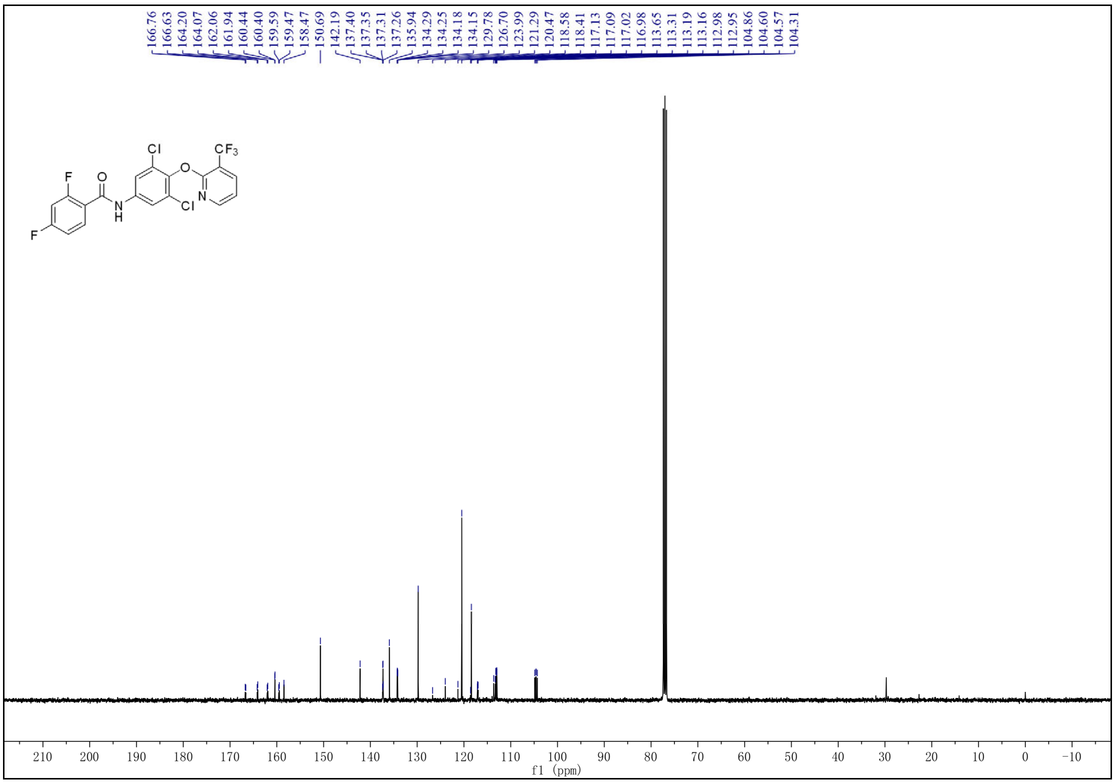

Fig. S59 ^{13}C NMR spectrum of compound **20**

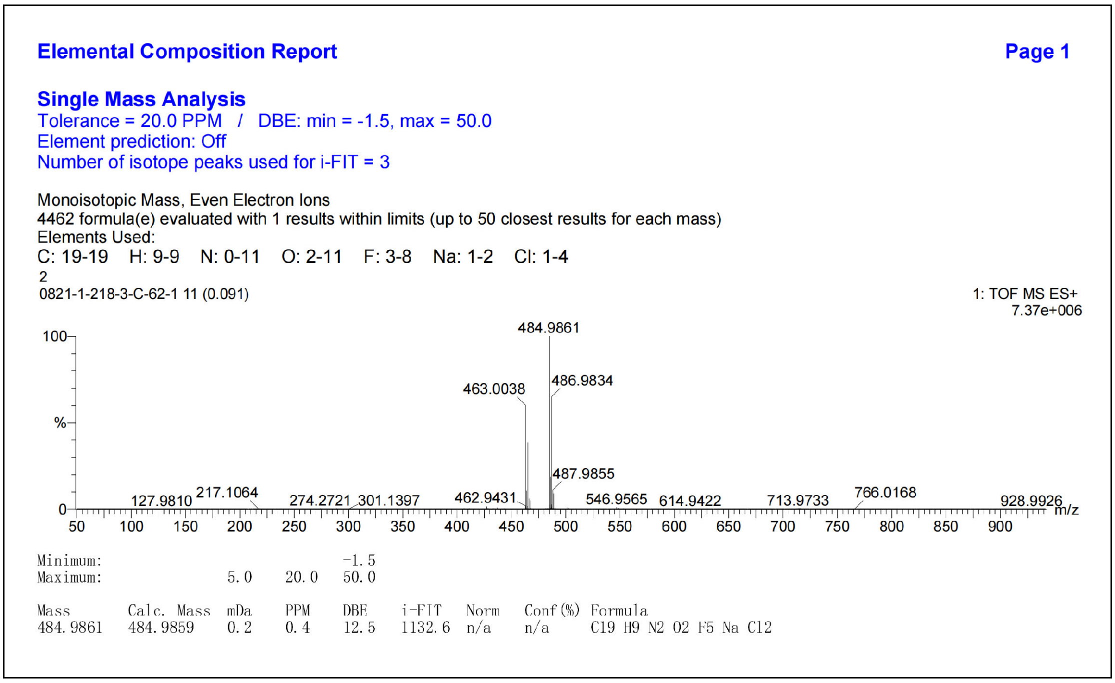

Fig. S60 HRMS spectrum of compound **20**

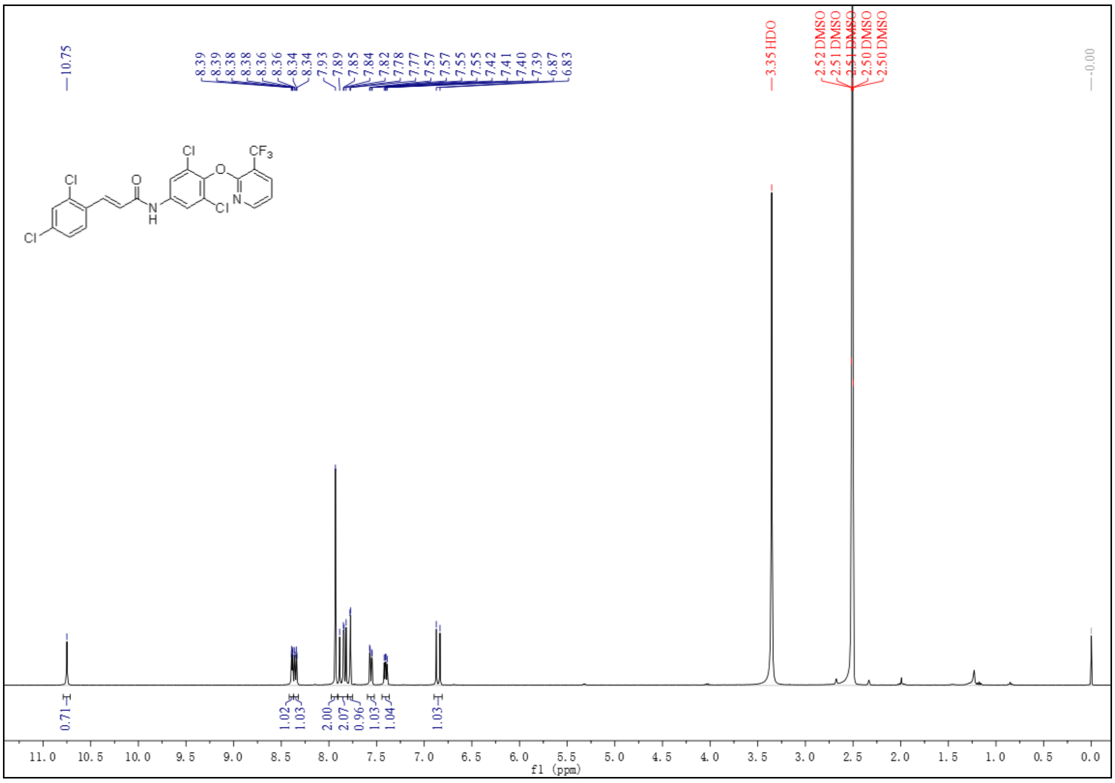

Fig. S61 ^{1}H NMR spectrum of compound **21**

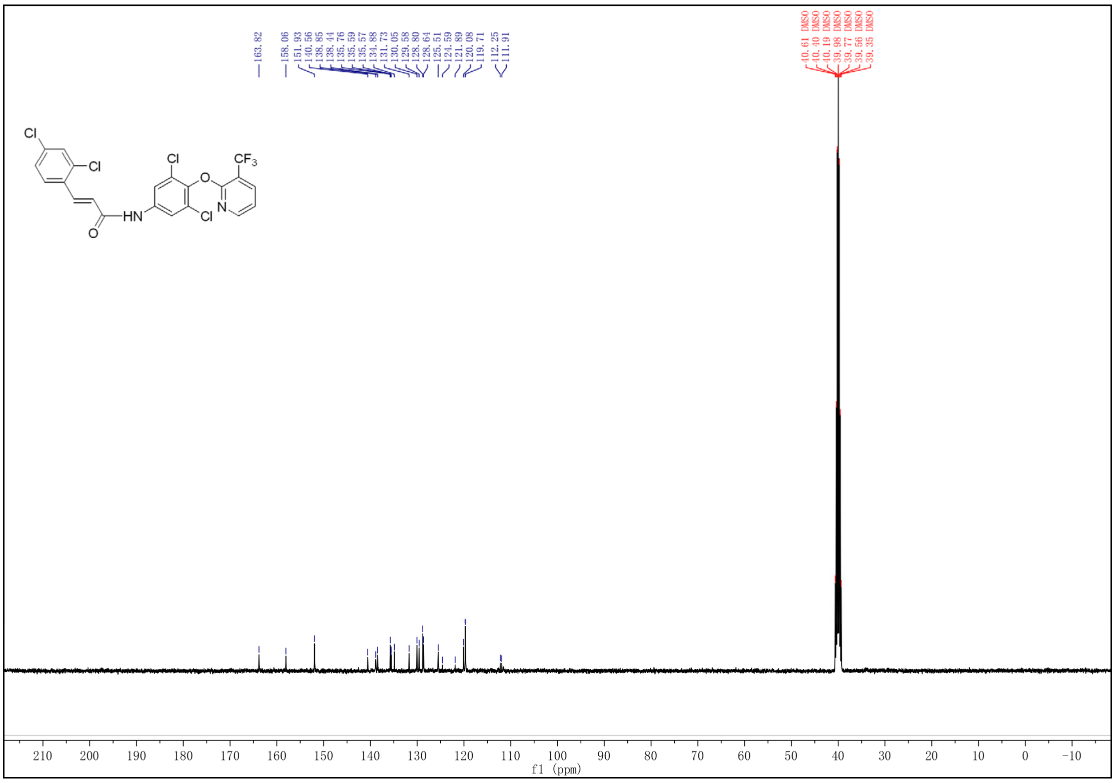

Fig. S62 ^{13}C NMR spectrum of compound **21**

Elemental Composition Report **Page 1**

Single Mass Analysis
Tolerance = 20.0 PPM / DBE: min = -1.5, max = 50.0
Element prediction: Off
Number of isotope peaks used for i-FIT = 3

Monoisotopic Mass, Even Electron Ions
4958 formula(e) evaluated with 1 results within limits (up to 50 closest results for each mass)
Elements Used:
C: 21-21 H: 11-11 N: 0-11 O: 2-11 F: 3-8 Cl: 1-4 Na: 1-2

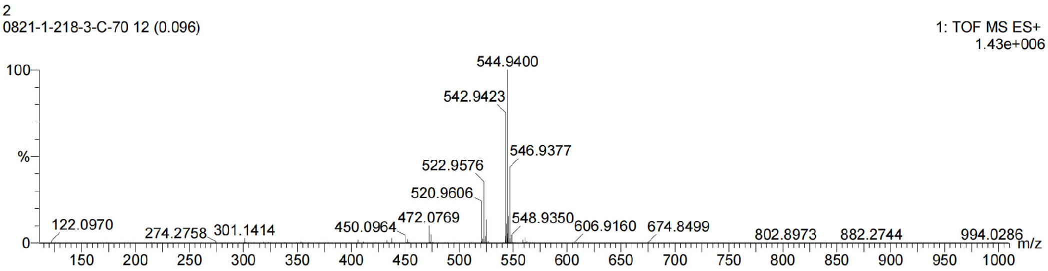

	Mass	Calc. Mass	mDa	PPM	DBE	i-FIT	Norm	Conf(%)	Formula
Minimum:					-1.5				
Maximum:			5.0	20.0	50.0				
	542.9423	542.9424	-0.1	-0.2	13.5	866.4	n/a	n/a	C21 H11 N2 O2 F3 Cl4 Na

Fig. S63 HRMS spectrum of compound **21**

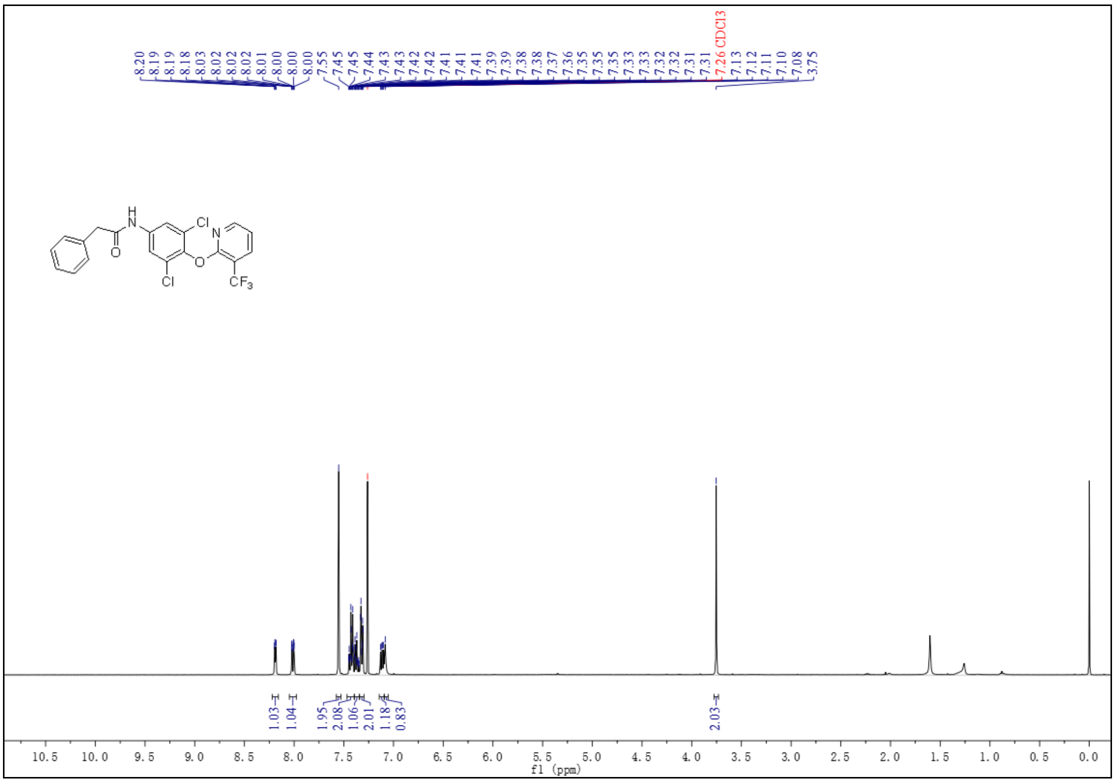

Fig. S64 ^{1}H NMR spectrum of compound **22**

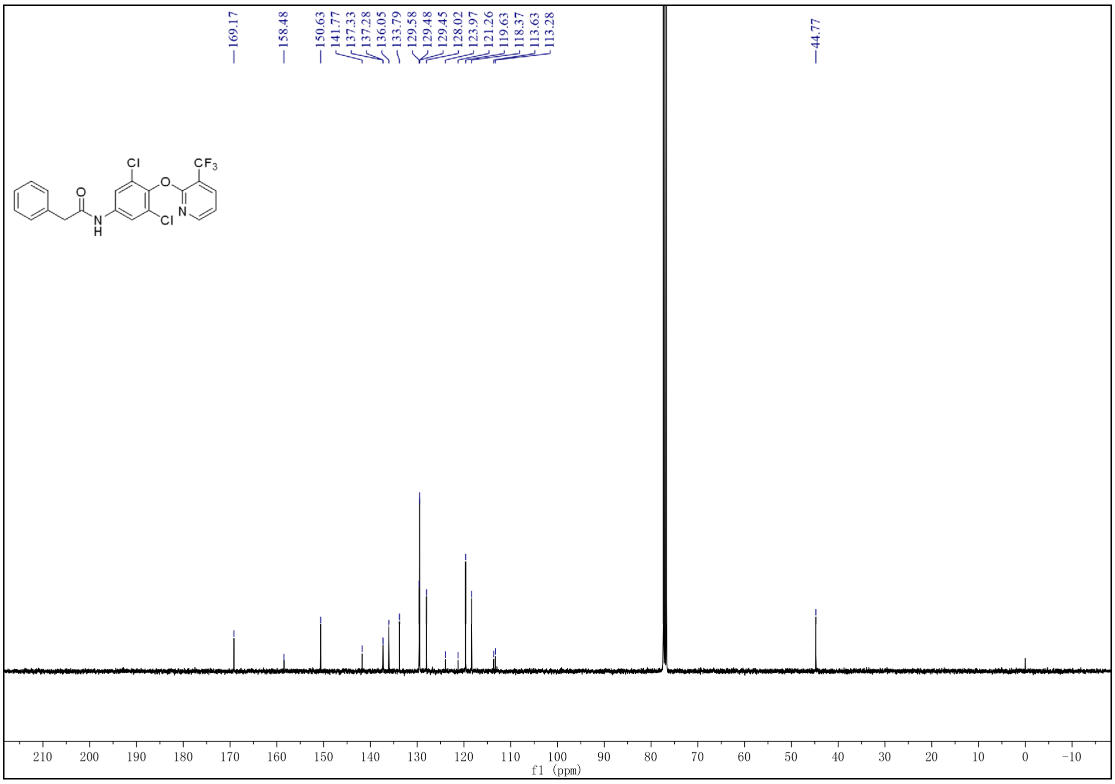

Fig. S65 ^{13}C NMR spectrum of compound **22**

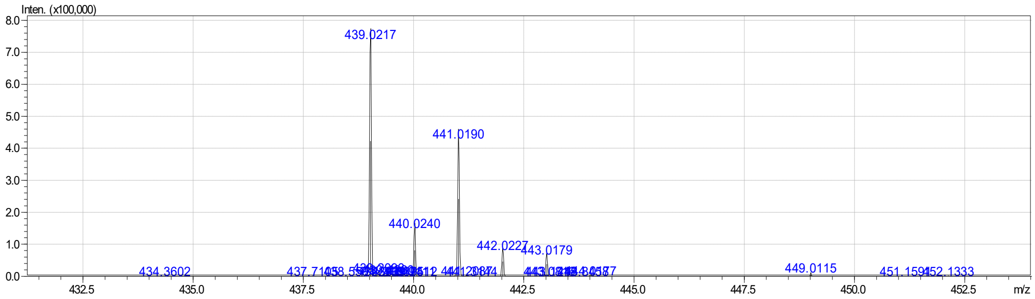

Fig. S66 HRMS spectrum of compound **22**

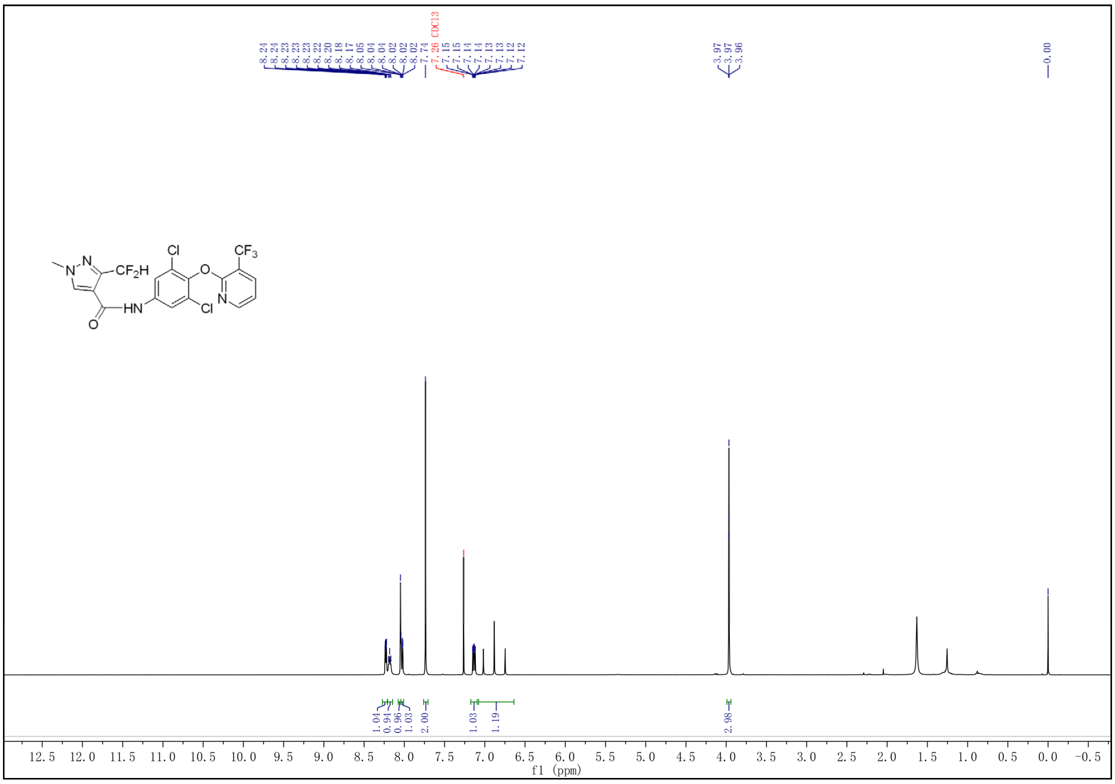

Fig. S67 ^{1}H NMR spectrum of compound **23**

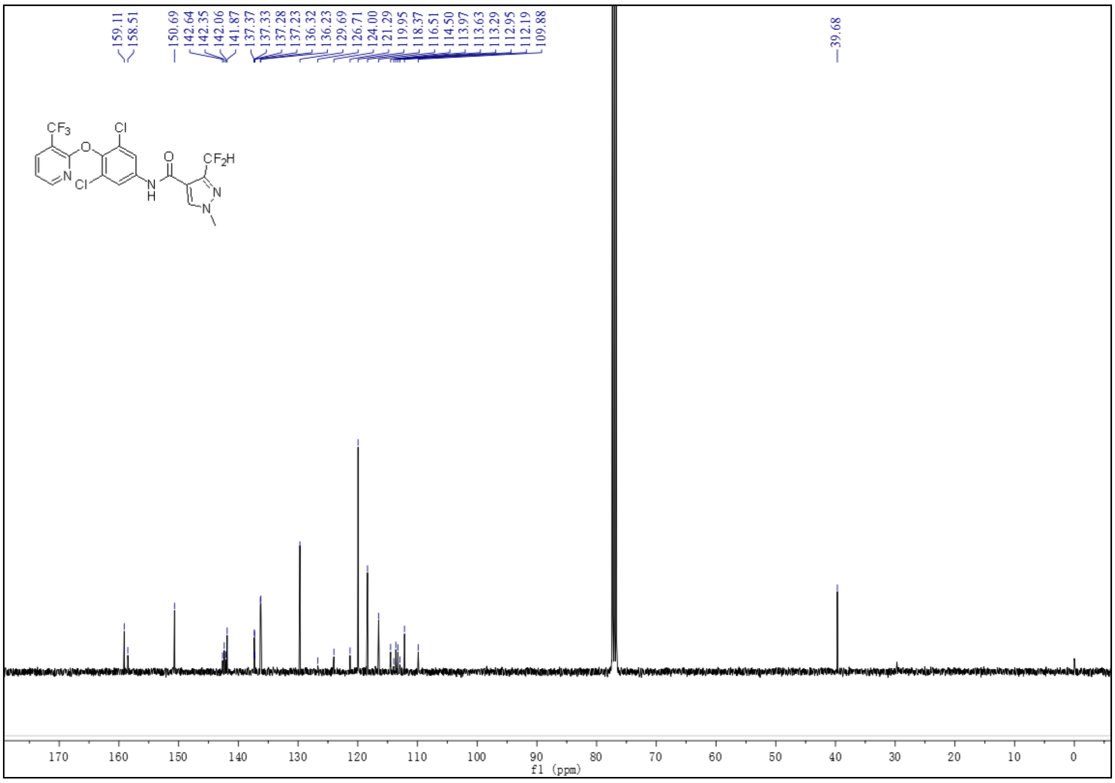

Fig. S68 ^{13}C NMR spectrum of compound **23**

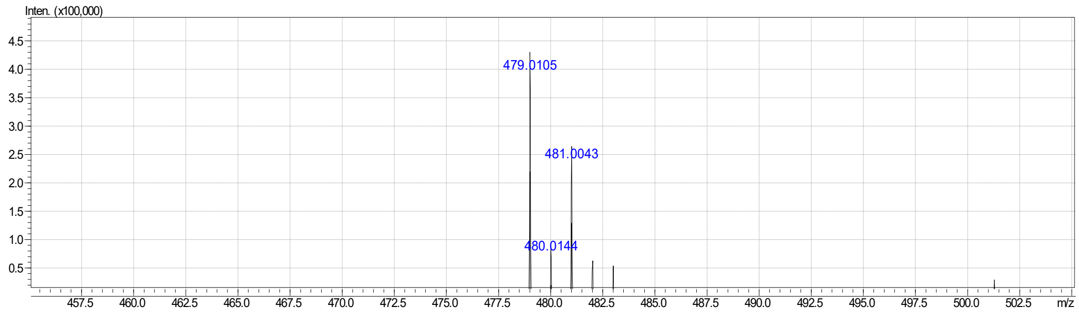

Fig. S69 HRMS spectrum of compound **23**

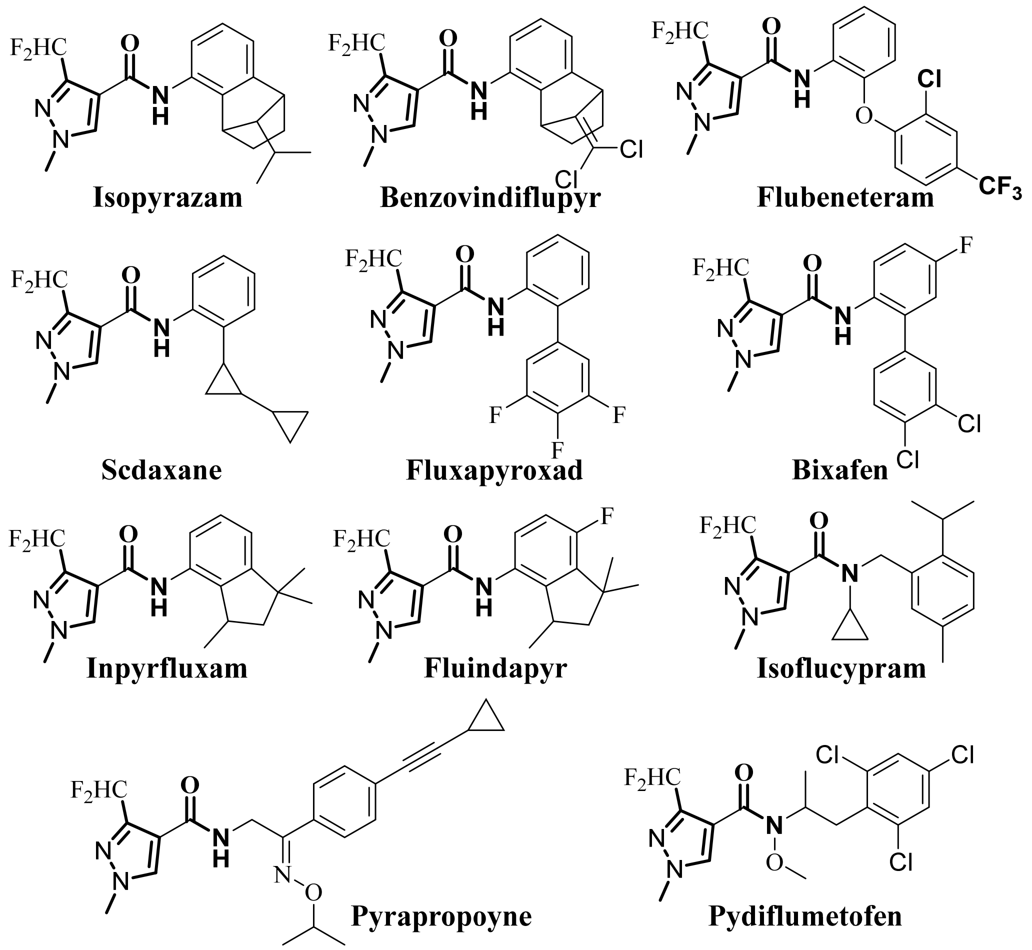

Fig. S70 Commercialized SDHIs bearing a pyrazole-4-carboxamide fragment as agricultural fungicides

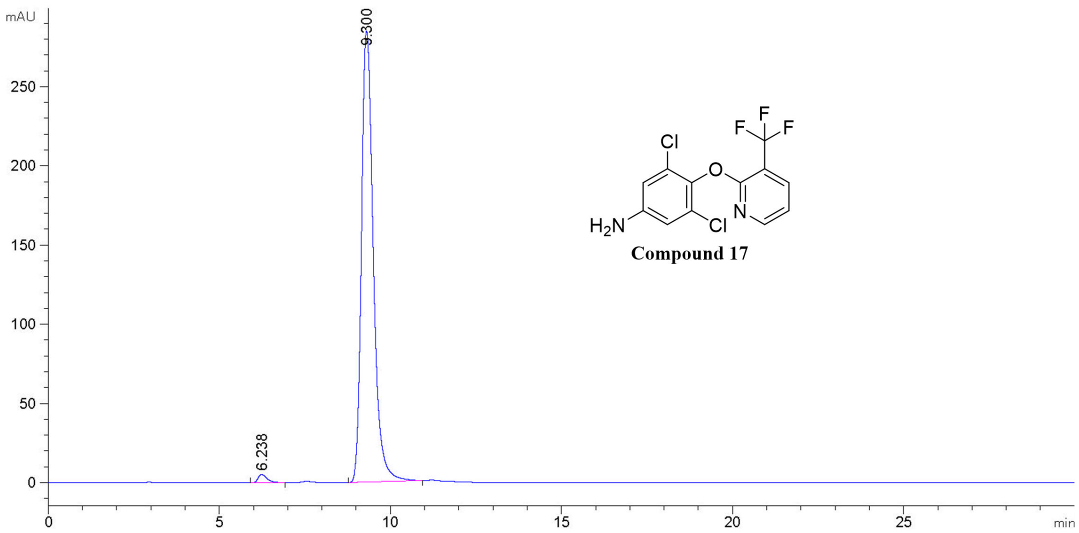

Peak	Retention Time (min)	Peak Shape	Peak Width (min)	Peak Area (mAU*s)	Peak Height (mAU)	Peak Area Percentage (%)
1	6.238	BB	0.2842	93.09061	5.06879	1.343
2	9.300	BB	0.3704	6838.35889	284.94409	98.657

Fig. S71 The purity test results of compound **17**

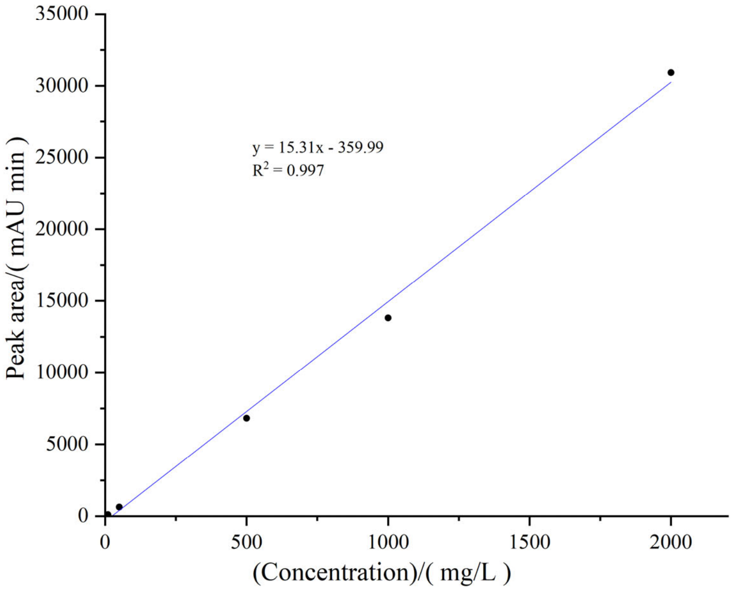

Fig. S72 The linear equation for compound **17**

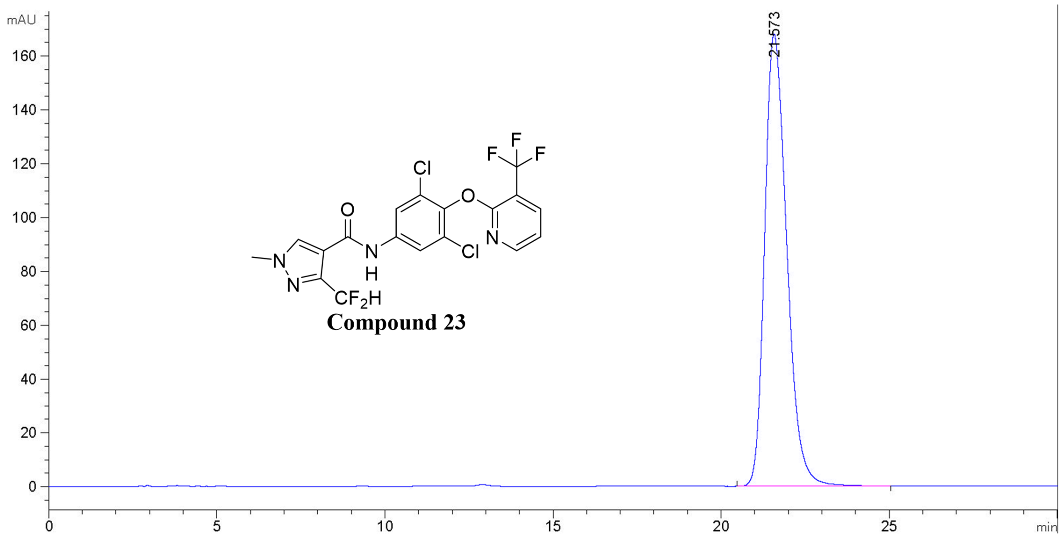

Peak	Retention Time (min)	Peak Shape	Peak Width (min)	Peak Area (mAU*s)	Peak Height (mAU)	Peak Area Percentage (%)
1	21.573	BB	0.7169	7657.68555	167.9937	100

Fig. S73 The purity test results of compound **23**

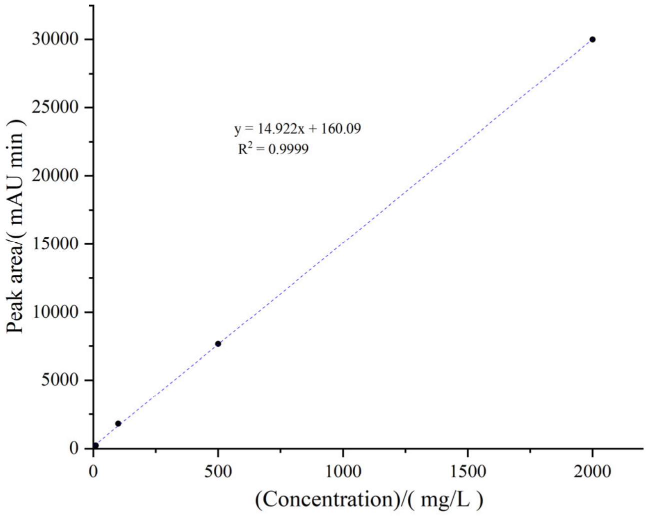

Fig. S74 The linear equation for compound **23**

Table S2. Degradation dynamics of compound **17** and **23** in banana peels (n = 3)

Time / h	**17** (mg/kg)	RSD / %	Time / h	**23** (mg/kg)	RSD / %
2	6.70±0.28	4.20	2	7.05±0.42	5.85
4	6.50±0.34	5.29	4	6.35±0.20	3.10
24	5.74±0.18	3.20	24	5.75±0.25	4.27
72	4.90±0.40	8.17	72	3.77±0.21	5.45
120	4.22±0.42	10.10	120	3.16±0.29	9.03

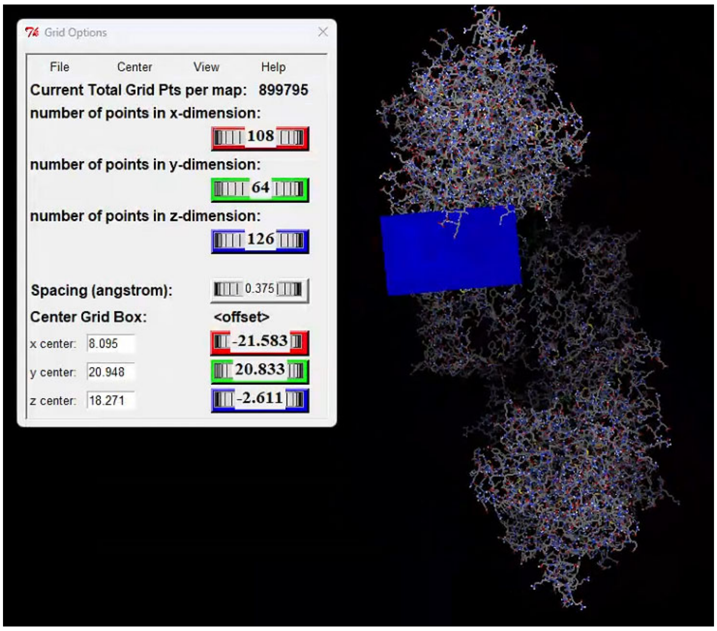

Fig. S75 Details on the molecular docking grid setup
